# Unveiling Termination Preferences and Screening of Structural Space in Multi-Metal MXenes

**DOI:** 10.1021/acsomega.5c04416

**Published:** 2025-07-15

**Authors:** Mauricio Mocelim, Henrique A. B. Fonseca, Pedro Ivo R. Moraes, Juarez L. F. Da Silva

**Affiliations:** São Carlos Institute of Chemistry, 153988 University of São Paulo, Av. Trabalhador São-Carlense 400, 13560-970 São Carlos, SP, Brazil

## Abstract

MXenes are promising two-dimensional materials for energy storage, catalysis, and electronics; however, our atomistic understanding of the stability mechanisms that rule the stability and physicochemical properties of multiple-metal MXenes is far from satisfactory. In this study, we investigated the configurational space, structural parameters, energetic stability, and the electronic properties of the MXenes (*M′M* *″*)_*n*+1_(*X′X* *″*)_*n*_O_2_ family, where M = Mo, Cr, Mn, Nb, V, Ti, Y, and X = C, N, B. We used density functional theory calculations within the Perdew–Burke–Ernzerhof functional including Hubbard corrections for the Cr, Mn, and V *d*-states. We identified a correlation between the magnitude of the occupation of the *M d*_*z* ^2^_-states and the preferential occupation of the O-sites on the MXene surface. Specifically, a reduced occupation of the *d*_*z* ^2^_-states leads to an energetic preference for the face-centered cubic sites, which is observed in most systems, except for Mo_2_CO_2_, CrMoNO_2_, MoVCO_2_ (Mo side), and MoNbNO_2_, where a higher occupancy of the *d*_*z* ^2^_-states promotes a preference for hexagonal close-packed sites. The in-plane configuration, that is, with metals or *X* mixed in the same layer, is more stable for MnNbCO_2_, MoNbNO_2_, NbYBO_2_, Ti_3_CNO_2_, and Nb_3_CNO_2_ MXenes, while the out-of-plane configuration, that is, with *M* or *X* separated in different layers, minimized the total energy for MoVCO_2_, CrMoNO_2_, and Ti_2_NbCNO_2_. Furthermore, we found a clear correlation between the work function and surface area and the chemical composition. As expected, the majority of our compositions are metallic, which is advantageous for applications of these materials as electrodes, e.g., in electrochemistry applications.

## Graphical abstract

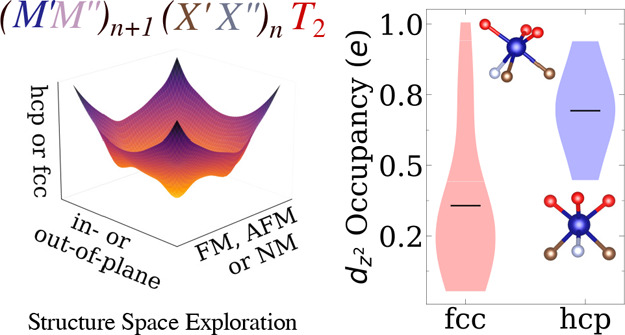

## 1 Introduction

MXenes are a promising family of two-dimensional (2D) materials obtained from layered MAX phases (*M*_*n*+1_ *AX*_*n*_) by etching the *A* element (group 8–16 element in the periodic table), where *M* is an early transition metal, and *X* are species with smaller atomic radius such as carbon (C), nitrogen (N), or boron (B).^1,2^ Therefore, given the potential variations in the selection of the *M*, *A*, and *X* species,^1^ a vast array of chemical compositions can be obtained, and hence, it can result in a diverse spectrum of physicochemical properties.^3,4^ For example, MXenes have been the subject of extensive research for a variety of applications, such as gas sensors,^5^ cathode for sodium-ion batteries,^6^ additives for perovskite solar cells,^7^ catalysts in the water–gas shift reaction,^8^ electrolyzers for hydrogen evolution reaction (HER),^9,10^ etc. Thus, the unique versatility and properties of MXenes make them crucial for the advancement of next-generation energy transition technologies.

The MXenes can be represented by the chemical formula (*M′M* *″*)_*n*+1_(*X′X* *″*)_*n*_ *T*_*x*_, where *n* ranges from 1 to 4, *M′M* *″* are commonly early transition metals elements, while *X* is C, N, B or a combination of them. Finally, *T* is the surface termination with *x* ranging from 0 up to 2. In MXene structures, the metal atoms are stacked in hexagonal closed-packed (hcp) structures, which have a space group of *P*63/*mmc*.^11^ If *M′* = *M* *″*, the MXene is called a single-metal compound; otherwise, if *M′*≠*M* *″*, the metal atoms can be separated in different layers or mixed in the same layer, i.e., several structural combinations are possible. The *X′X* *″* atoms are located in the octahedral interstitial sites, while the *T* sites can be occupied by O, OH, NH, F, Cl, Br, S, Se, Te or a combination of them located in the hcp or face-centered cubic (fcc) sites.^11^ For both *M* and *X* species, the formation of out-of-plane double *M*, *X* (o^*M*^-MXene and o^*X*^-MXene) or in-plane double *M*, *X* (i^*M*^-MXene and i^*X*^-MXene) structures is feasible, i.e., species can be spatially segregated into distinct layers or integrated within the same layer, respectively.

The termination of MXenes, indicated here by *T*, has a strong dependence on the synthesis method. For example, (i) when concentrated hydrogen fluoride (HF) is used, the H^+^ oxidizes the *A* element and F^–^ binds to it, resulting in its removal and F, OH and O terminations; (ii) the use of molten salts has a similar acid–base mechanism but leads to Cl terminations; (iii) when water-free ammonium hydrogen fluoride (NH_4_HF_2_) is used, the termination is F-rich.^12^ However, postsynthesis heating combined with sonication can be used to obtain desirable terminations and single-layer sheets.^2^ In addition to these structural possibilities, more recently these materials were predicted to form random solid metal solutions, oxycarbides, or even high-entropy MXenes,^13^ i.e., a wide range of possibilities are possible for the structures.

About 40 MXenes have been synthesized experimentally and more than 100 have been predicted by theoretical calculations.^12^ Most of the MXenes reported experimentally are carbides; once the layers of nitride are dispersed during treatment with HF. With this discussion, we intend to emphasize that, although MXenes can have diverse compositions and structures, more than 70% of all research studies have focused only on the Ti_3_C_2_ *T*_*x*_ compounds with different terminations due to easy top-down synthesis from stable titanium aluminum carbide (Ti_3_AlC_2_) precursors.^1^ Given the extensive uncharted regions in terms of composition and structural diversity, it is imperative for forthcoming research to extend beyond Ti-based MXenes. Notable examples of successful ventures into nitride exploration include the synthesis of Mo_2_N*T*_*x*_, V_2_N*T*_*x*_, and nitrogen-doped Ti_2_C*T*_*x*_.^14,15^

Theoretical calculations based on density functional theory (DFT) calculations usually assume the fcc sites for *T* terminations and only out-of-plane MXenes configurations. Several studies have modeled a large number of double-metal MXenes, but only o^*M*^-MXene were considered in the majority of investigations.^16,17^ This assumption oversimplifies the exploration of the structure space, e.g., both o^*M*^-MXene and i^*M*^-MXene can be experimentally synthesized.^18^ The same problem occurs for carbonitride structures, e.g., only random distribution has been experimentally observed,^19^ however, theoretical studies considered only o^*X*^-MXene.^20,21^

In many theoretical studies, especially when considering double-metal MXenes, several assumptions are made, which are not justifiable. In this work, we aim to explore the structure space of MXenes to obtain insights into the atomic structures and their relations to the electronic structure. We selected the following compositions based on recent literature reports as relevant materials for HER, which is a promising application of MXenes: Mo_2_CO_2_,^22^ Cr_2_CO_2_,^23^ MnNbCO_2_,^17^ MoVCO_2_,^17^ CrMoNO_2_,^17^ MoNbNO_2_,^17^ NbYBO_2_,^17^ Ti_3_C_2_O_2_,^24,25^ Ti_3_CNO_2_,^20^ Nb_3_CNO_2_,^20^ Ti_2_NbCNO_2_,^21^ V_4_C_3_O_2_,^26^ Nb_4_C_3_O_2_,^27,28^ Cr_4_C_3_O_2_.^29^ In our calculations, we considered only O terminations because of its great stability and suitability for HER.^13,17,30,31^ Furthermore, by considering only a single termination, it will be possible to perform a deep exploration of the structural configuration space of the MXenes, and hence, a solid contribution to the field of 2D materials.

## 2 Theoretical Approach and Computational Details

### 2.1 Total Energy Calculations

Our calculations were based on the spin-polarized DFT^32,33^ framework, as implemented in the Vienna Ab initio simulation package (VASP), version 5.4.4.^34^ Local or semilocal approximations for the exchange-correlation (XC) energy functional face challenges in describing the long-range van der Waals (vdW) interactions. Thus, to improve the accuracy of our results, we used the semiempirical vdW D3 correction^35^ for all calculations, which incorporates an attractive component of the total energy, and adjusts the equilibrium lattice parameters (*a*_0_) closer to the experimental values.^36,37^ The semilocal formulation proposed by Perdew–Burke–Ernzerhof (PBE)^38^ for the XC energy functional has limitations in providing an accurate description of the localization of the *d*-states in several MXenes, particularly those compositions that include Cr, Mn, and V, e.g., Cr_2_CO_2_, MnNbCO_2_, MoVCO_2_, CrMoNO_2_, V_4_C_3_O_2_, and Cr_4_C_3_O_3_.^4,39,40^ In particular, it affects mainly the magnitude of magnetic moments, adsorption site of the *T* species, and the magnitude of the electronic band gap, however, it yields good equilibrium lattice parameters.^36,41,42^

Based on these observations, we used the following strategy: (i) All MXene structures selected for different compositions were initially optimized using the PBE XC approximation. (ii) All MXenes with Cr, Mn and V in their chemical composition were reoptimized using the PBE + *U* approximation, which can improve the description of the localization of the *d*-states, and hence provides an improvement in the description of the physical-chemical properties. Here, we used the rotationally invariant approach^43^ with an effective Hubbard parameter (*U*_eff_) of 4 eV applied to metals *d*-states, which yields magnetic properties similar to those obtained by hybrid functionals such as the Heyd–Scuseria–Ernzenhof (HSE).^39^

Electron–ion interactions were described using the full-potential projector augmented wave (PAW) method^44,45^ in combination with a set of plane wave bases to represent the Kohn–Sham (KS) states. The MXene structures were modeled using repeated slab geometry with a 2 × 2 unit cell and a vacuum thickness of 15 Å in the *z* direction, resulting in negligible interactions between the slab and its periodic images. To determine the equilibrium structures, we performed optimizations of the stress tensor components in the *xy* plane, concurrently with the optimization of forces in all directions.

A plane wave cutoff energy of 868.862 eV was employed, which is twice the largest recommended cutoff energy specified by the selected PAW projectors (Mo, Cr, Mn, Nb, Mo, V, Ti, Y, C, N, B and O) due to the slower convergence of the stress tensor with respect to the number of plane waves. For all optimizations, the equilibrium structure was reached once the atomic forces were smaller than 1 × 10^–2^ eV Å^–1^ on all atoms using the self-consistency criterion of 1 × 10^–6^ eV for the total energy. Furthermore, to improve the stability of the self-consistency process, we used a Gaussian smearing parameter of 0.01 eV. For the remaining properties, we used a plane wave cutoff energy of 488.735 eV, that is, 12.5% higher than the largest recommended value.

To obtain an optimum **k**-mesh for each MXene composition, which can yield good results at a lower computational cost, we performed several computational convergence calculations. We considered a maximum error of 1 and 0.75% for cohesive energies (*E*_coh_) and *a*_0_, respectively, compared with well-converged results. For example, the equilibrium structure was obtained for one configuration using a unit cell 1 × 1 for each MXene composition as a function of the **k**-meshes, i.e., 4 × 4 × 1, 8 × 8 × 1, 16 × 16 × 1 and 32 × 32 × 1. Thus, we find that a **k**-mesh of 2 × 2 × 1 is enough to produce accurate results for Cr_2_CO_2_, MoVCO_2_, CrMoNO_2_, MoNbNO_2_, Ti_3_C_2_O_2_, Ti_3_CNO_2_, Nb_3_CNO_2_, Ti_2_NbCNO_2_, V_4_C_3_O_2_, Nb_4_C_3_O_2_, and Cr_4_C_3_O_2_ in units 2 × 2, while it is increased for 4 × 4 × 1 **k**-mesh for Mo_2_CO_2_, MnNbCO_2_, and NbYBO_2_. Moreover, for the purpose of calculating the density of states (DOS) and assessing the band structure, the **k**-mesh was expanded to 32 × 32 × 1 and 16 × 16 × 1 for unit cells 1 × 1 and 2 × 2, respectively, to enhance the representation of the electronic states in proximity to the maximum valence band and the minimum conduction band.

### 2.2 MXenes and Atomic Structure Configurations

We selected the following compositions of MXenes, which are categorically divided into two groups: experimentally synthesized^23–28,46,47^ and theoretically predicted.^17,20,21,29^ The first group includes: Mo_2_CO_2_,^47^ Cr_2_CO_2_,^23^ Ti_3_C_2_O_2_,^24,25^ Ti_3_CNO_2_,^46^ V_4_C_3_O_2_,^26^ Nb_4_C_3_O_2_,^27,28^ and Ti_2_NbCNO_2_,^21,48^ while the second group includes: MnNbCO_2_,^17^ MoVCO_2_,^17^ CrMoNO_2_,^17^ MoNbNO_2_,^17^ NbYBO_2_,^17^ Nb_3_CNO_2_,^20^ and Cr_4_C_3_O_2_.^29^ MXenes with Mo and V have been synthesized, but only for a larger number of layers, e.g., MoV_3_C3*T*_*x*_.^49^ For Ti_2–*y*_Nb_*y*_C*T*_*x*_, there are reports of synthesis with various compositions with unlimited solubility and random distribution of metals.^48^

Figure 1 shows a schematic representation for MXenes with *ABC* stacking (*M* and *X* layers). This stacking was considered because the MAX phases, which are precursors of MXenes, are generally more stable for *ABC* stacking.^50^ For the considered MXenes compositions, to the best of our knowledge, there are no experimental reports of preference for *AB* stacking.^51^ The MXene structures can differ in the preference of the surface termination site and in the number of *M* layers. Furthermore, more than one species can be found in the metal and *X* layers, and hence larger unit cells are required to model these systems. In this work, we used a 2 × 2 unit cell, which yields additional configurations for the structure space. Thus, the selected MXenes are single or double-metals with the formula (*M′M* *″*)_*n*+1_(*X′X* *″*)_*n*_O_2_, where *M′* and *M* *″* represent metal species, while *X′* or *X* *″* is either C, N or B. The O species are located within the fcc or hcp sites. Moreover, a number of the selected MXenes exhibit magnetic properties, necessitating the consideration of nonmagnetic (NM), ferromagnetic (FM), ferrimagnetic (FIM) and antiferromagnetic (AFM) configurations, which increases the number of calculations substantially.

**Figure 1:**
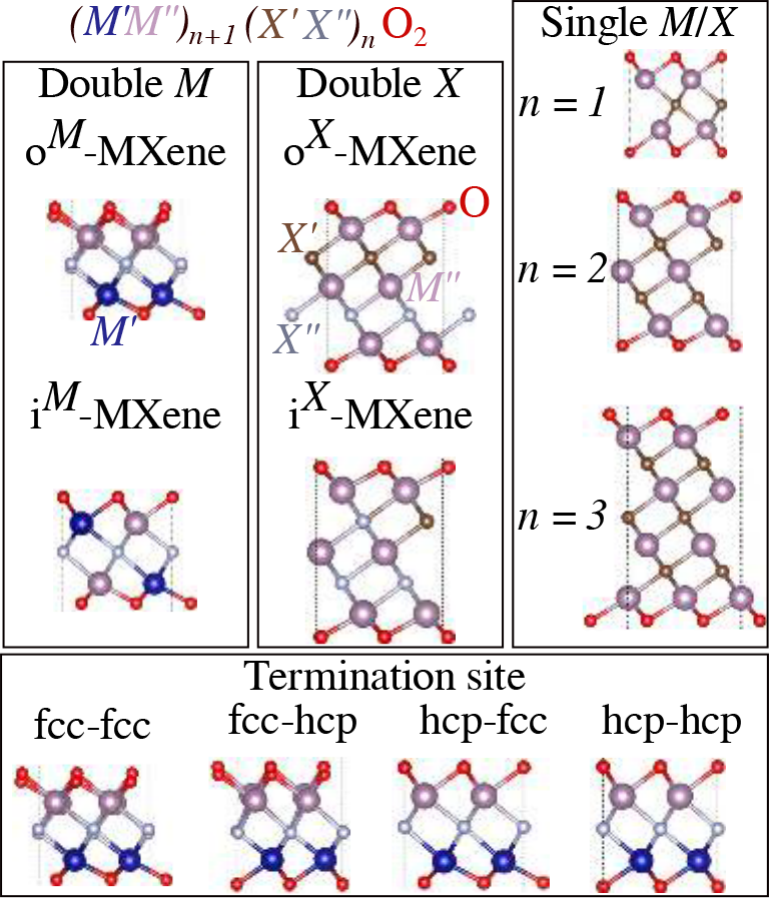
Schematic representation of the selected MXenes structures, including *M* and *X* species forming in-plane and out-of-plane structures, fcc and hcp *T* surface terminations, and MXenes with different numbers of layers.

Figure 2 shows a schematic representation of the exploration of the configuration space to obtain the lowest energy configurations for the selected MXenes. Based on this diagram, it yields about 240 stress tensor calculations, which can be explained as follows: (i) Initially, we considered the initial configurations of o^*M*^-MXenes, i^*M*^-MXenes, o^*X*^-MXenes, i^*X*^-MXenes, FM, and AFM. We also considered fcc and hcp sites for oxygen termination; (ii) We further explored only the most promising region, for example, for Nb_3_CNO_2_, after initial exploration, the lowest energy region was fcc-fcc-i^*M*^-MXene, then we calculated several other configurations of i^*M*^-MXene to obtain the lowest energy structure. All systems with a total magnetic moment of 0.1 μ_B_/atom were classified as FM or FIM; (iii) For systems containing Cr, Mn or V, we further explored the structure space using PBE + *U*, that is, we considered different structures from PBE calculations within a range of 100 meV.

**Figure 2:**
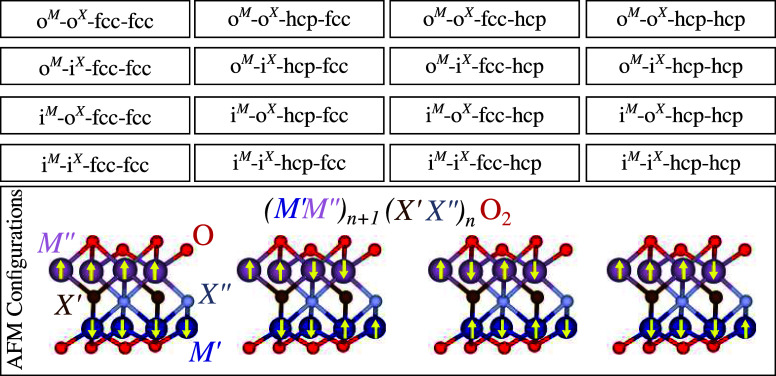
Schematic representation of structure possibilities for the out-of-plane and in-plane MXenes, represented by the letters o- and i-, respectively. The possible oxygen sites are labeled as fcc or hcp. The electronic AFM, FM, FIM, and NM configurations were considered but are not shown here.

## 3 Results and Discussion

The most significant trends and results derived from this study are summarized and analyzed below. We begin our discussion with all results from the structural space exploration, namely the PBE calculations. Then, we discuss the differences between PBE + vdW and PBE + vdW + *U* for the selected systems. Finally, we focus on the lowest energy structures, which include several physical-chemical properties. We applied ligand field theory (LFT) to the lowest-energy structures to explain termination sites. We also analyzed the covalent, ionic, and metallic character of chemical bonds in MXenes. Finally, we explore the relationship between bond character and the band gap of the systems. In the last section, we discuss potential applications of MXenes based on the knowledge obtained throughout this paper.

### 3.1 Screening of the Structural Configurational Space via PBE Calculations

#### 3.1.1 Relative Energies

Figure 3 summarizes the relative total energy (Δ*E*_tot_) for all optimized configurations, where Δ*E*_tot_ = *E*_tot_^*i*^ – *E*_tot_^*lowest*^. *E*_tot_^*i*^ is the total energy of the configuration *i* and *E*_tot_^lowest^ is the lowest total energy configuration. We found that most of the optimized structures are within Δ*E*_tot_ of 301.785 meV/atom. The exception is MnNbCO_2_, where the highest energy is at 631.998 meV/atom. Furthermore, there are many configurations within the relative total energy precision limit, i.e., smaller than 5 meV/atom. For example, several FIM, AFM, and NM configurations obtained for Cr_2_CO_2_, CrMoNO_2_, and Cr_4_C_3_O_2_ are degenerate.

**Figure 3:**
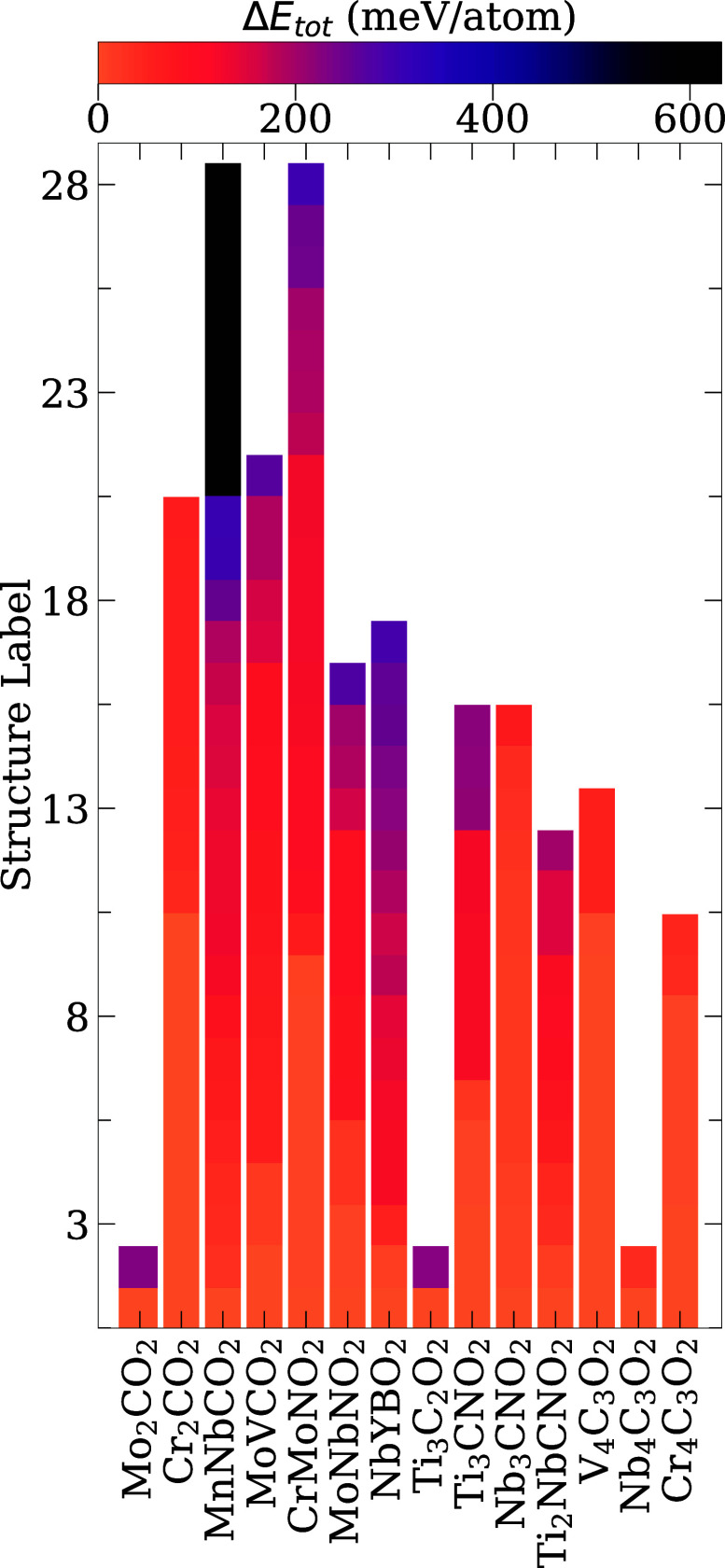
Heat map of the PBE Δ*E*_tot_ results for all optimized MXene configurations. The structure labels on the *y*-axis are provided solely for visual representation, where each point on the *y*-axis corresponds to a specific configuration. The energies and configurations of all calculations, including in- and out-of-plane, hcp, fcc, and magnetic configurations, are available in the electronic Supporting Information file. Single metal NM systems have few possibilities, while the number of structural/magnetic configurations increase for double metal magnetic systems, specially due to assessment of AFM possibilities.

#### 3.1.2 Termination Site

For most systems, the O species have an energetic preference for the fcc sites, whereas the hcp sites are obtained for systems composed of Mo atoms. The behavior discussed previously does not occur in MoNbNO_2_ because these i^*M*^-MXene have an equal number of both metals in each layer. Systems with Nb atoms prefer the fcc sites in most cases, except for MoNbNO_2_ due to the presence of Mo, as discussed before. We will return to this discussion of the fcc or hcp preference in Section 3.5, where we discuss electronic structure.

#### 3.1.3 In- and Out-of-Plane

All carbonitrides have a preference for the i^*X*^-MXene, except for Ti_2_NbCNO_2_. These results can guide other theoretical works, i.e., o^*X*^-MXene should not be the first choice of structure. For double-metal MXenes, our results indicate that only structure exploration can lead to the lowest energy structure, i.e., half of our MXenes prefer i^*M*^-MXene structures while the other half prefers o^*M*^-MXene structures.

### 3.2 Investigation of the Role of the Hubbard *U* Correction

As shown in Table 1, the addition of Hubbard correction *U* (PBE + *U*) in MXene systems containing Cr, Mn, and V induces significant changes in both their structural and electronic/magnetic properties, which is consistent with previous results.^4,39^ For example, in general, the addition of *U* results in a modest increase in the thicknesses *a*_0_ and MXene thicknesses (*d*_*z*_) across all selected compounds. This behavior aligns with a decrease in the delocalization of *d*-states, which consequently weakens the covalent bonding and leads to expanded interatomic distances. As expected from previous studies,^4,39^ PBE + *U* tends to enhance the magnitude of local magnetic moments (*m*_loc_) and, in numerous instances, modifies the total magnetic moment (*m*_tot_) of the unit cell. For example, MXenes characterized as NM or exhibiting weak magnetism under PBE frequently become magnetically ordered under the PBE + *U* approximation, exhibiting FM or AFM configurations. Furthermore, while several systems remain metallic after incorporation of *U*, others undergo a bandgap (*E*_g_) opening or widening. For certain systems, PBE + *U* predicts a divergent lowest-energy configuration relative to PBE, underscoring the necessity of accounting for localization effects in structural optimizations.

**Table 1:** Comparison between the PBE and PBE + *U* for MXenes Compositions with Cr, Mn or V^a^

system	final conf.	functional	*a*_0_	*d*_*z*_	*E*_g_	*m*_tot_	*m*_loc_^*M′*^	*m*_loc_^*M″*^
Cr_2_CO_2_	hcp	PBE	2.68	4.85	metal			
	fcc	PBE + *U*	3.01	4.93	0.36	0.00	2.41	
MnNbCO_2_	i-fcc-fcc	PBE	3.01	4.55	metal	8.10	2.19	0.11
		PBE + *U*	3.14	4.61	metal	16.48	4.26	0.06
MoVCO_2_	o-hcp-fcc	PBE	2.89	4.80	metal	3.54	0.06	0.85
		PBE + *U*	2.92	4.81	metal	4.03	0.04	1.18
CrMoNO_2_	o-fcc-hcp	PBE	2.87	4.85	0.56	10.84	2.58	0.09
	o-hcp-hcp	PBE + *U*	2.89	4.91	0.87	0.00	2.62	0.03
V_4_C_3_O_2_	fcc	PBE	2.85	9.23	metal			
		PBE + *U*	2.96	9.35	metal	0.00	0.80	
Cr_4_C_3_O_2_	hcp	PBE	2.77	9.50	metal	0.00	0.28	
	fcc	PBE + *U*	2.94	9.38	metal	32.46	2.35	

a In this table, we show only the lowest energy structure for each functional. We show the average of *a*_0_ and *b*_0_ and *d*_*z*_, in Å. The *E*_g_ is given in eV. The *m*_tot_ and *m*_loc_ are the total and modulus of metal local magnetic moments in units of (μ_B_) and (μ_B_/atom), respectively.

The magnitude of the effects induced by the addition of the Hubbard *U* correction is different for each system. For example, Cr_2_CO_2_ shows a substantial response to the addition of *U*: (i) The favored O termination changes from hcp (PBE) to fcc (PBE + *U*), with a significant increase in *a*_0_ from 2.68 to 3.01 Å; (ii) PBE predicts a metallic solution, while PBE + *U* yields a moderate *E*_g_ of (0.36 eV) along with the development of considerable local magnetic moments on the Cr atoms, which are arranged AFM. With respect to MnNbCO_2_, the structural configuration remains unchanged (i-fcc-fcc) with PBE + *U*, although *a*_0_ expands from 3.01 to 3.14 Å. The metallic nature is retained; nevertheless, the magnetic properties are considerably modified: the total magnetic moment nearly doubles and the local Mn moment increases from 2.19 to 4.26 μ_*B*_/atom, indicating a substantial enhancement of the magnetic ordering under PBE + *U*.

For compound MoVCO_2_, the structural configuration of o-hcp-fcc is consistently retained between PBE and PBE + *U*, the material that maintains its metallic nature. Only marginal increases are observed in both *a*_0_ and *d*_*z*_, and the alterations in magnetic properties are comparatively moderate: a minor increase in total magnetic moment and an enhancement of the local magnetic moment in V from 0.85 to 1.18 μ_B_/atom. In contrast, for CrMoNO_2_, there is a structural transition from o-fcc-hcp (PBE) to o-hcp-hcp (PBE + *U*), accompanied by a slight increase in *a*_0_. For V_4_C_3_O_2_, the *T* site is retained for both approximations and we observed a notable increase in *a*_0_, that is, a difference of 0.17 Å between PBE and PBE + *U*. A similar increase occur for *d*_*z*_, however the material retains its metallic nature for both approximations. Furthermore, the lowest energy structure changes from NM to AFM with a *m*_loc_ of 0.80 μ_*B*_/atom.

We found significant changes for Cr_4_C_3_O_2_: the structural configuration changes from hcp as determined by PBE to fcc as determined by PBE + *U*, which is accompanied by a substantial increase in the lattice parameter. PBE + *U* indicates a significant alteration in magnetic properties, in which the total magnetic moment escalates from zero to 32.46 μ_*B*_, and the local Cr moments become markedly larger, suggesting intensified ferromagnetic ordering.

For Cr_2_CO_2_, Bera and Kumar found a metallic FM structure in their work,^4^ however, they considered 2 × 1 supercells, and we should highlight that, as energy differences are in the range of meV/atom, different computational parameters play an important role, e.g., the **k**-mesh, supercell size, and initial magnetic configuration. Another evidence of the complexity of Cr_2_CO_2_ is the fact that Tan et al.^39^ found a hcp-FM lowest energy structure, but the energy differences in their work were also within a range of meV/atom. For V_4_C_3_O_2_, our *a*_0_ from PBE + *U* matches the experimental results available.^52^ For the other systems, we found no studies in the literature for comparison.

### 3.3 Structure Features of the Lowest Energy Configurations

Figure 4 shows the lowest energy structure for each composition from our PBE and PBE + *U* calculations. In the following, we summarize the most important results. Our calculations show that the fcc O-terminations minimize energy for most cases, namely Cr_2_CO_2_, MnNbCO_2_, MoVCO_2_ (V side), NbYBO_2_, Ti_3_C_2_O_2_, Ti_3_CNO_2_, Nb_3_CNO_2_, Ti_2_NbCNO_2_, V_4_C_3_O_2_, Nb_4_C_3_O_2_, and Cr_4_C_3_O_2_. The remaining systems prefer hcp O-terminations, namely, Mo_2_CO_2_, MoVCO_2_ (Mo side), CrMoNO_2_, and MoNbNO_2_.

**Figure 4:**
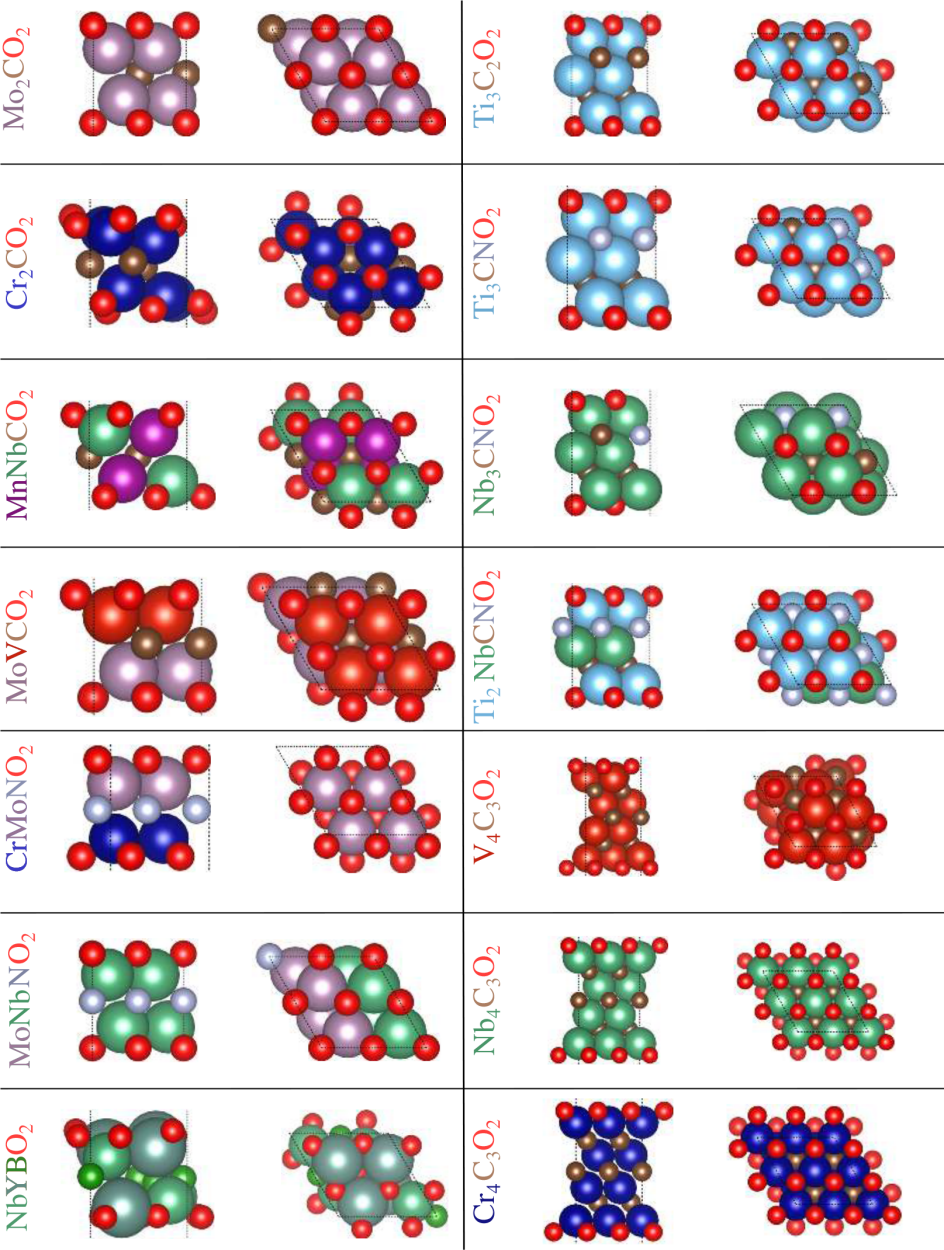
Side (left) and top (right) views of the lowest energy structures for all MXene compositions.

The i^*M*^-MXenes are more stable for MnNbCO_2_, NbYBO_2_, and MoNbCO_2_ while o^*M*^-MXene minimizes the energy in CrMoNO_2_, Ti_2_NbCNO_2_, and MoVCO_2_. Carbonitrides are more stable in the i^*X*^-MXene configuration, except Ti_2_NbCNO_2_. Our results are consistent with the literature for the systems Mo_2_CO_2_ and V_4_C_3_O_2_.^52,53^ For Ti_3_CNO_2_, Nb_3_CNO_2_, and Ti_2_NbCNO_2_ the literature has only considered o^*X*^-MXenes,^20,21^ but our calculations show that i^*X*^-MXenes yield the lowest energy.

The systems MoNbNO_2_ and NbYBO_2_ were previously investigated in the literature; however, only fcc-fcc-o^*M*^-MXene structures were considered, as exemplified in the machine learning work by Abraham et al.^17^ Our calculations demonstrate that for both systems, hcp-hcp-i^*M*^-MXene structures represent the lowest energy configurations. Interestingly, in the 2 × 2 cell NbYBO_2_ structure, three of the B atoms are aligned in the lowest energy structure; see Figure 4. The NbYBO_2_ MXene (MBene) has not yet been synthesized. Previous literature reports for other MBenes indicate that these materials can have hexagonal or orthorhombic structures.^54,55^ Our results indicate that this subclass of materials should be further explored theoretically and experimentally. To date, we do not have experimental work to compare these results for NbYBO_2_. A similar distortion of *X* sites was also observed for Cr_2_CO_2_. With regard to electronic structure, the lowest-energy structure of most systems is NM, which is expected for oxygen-terminated MXenes. The systems MnNbCO_2_ and MoVCO_2_, and Cr_4_C_3_O_2_ are FIM. For Cr_2_CO_2_, CrMoNO_2_ and V_4_C_3_O_2_ the lowest energy structure was calculated as AFM.

### 3.4 Lattice Parameters, Cohesive Energies, and Magnetic Trends in MXenes

In Table 2, we present the structural, energetic, electronic, and magnetic properties of the lowest-energy configurations calculated with the PBE and PBE + *U*. For the equilibrium lattice parameters, we report only the average of *a*_0_ and *b*_0_ because the differences between these constants are negligible (smaller than 0.083 Å for Cr_2_CO_2_), reinforcing the near hexagonal symmetry of the structures. Individual values of *a*_0_ and *b*_0_ are available in the electronic Supporting Information file. Our lattice constants agree excellently with the values in the literature for Mo_2_CO_2_, Ti_3_C_2_O_2_, Ti_2_NbCNO_2_, and V_4_C_3_O_2_. For example, for Ti_3_CNO_2_, Nb_3_CNO_2_, and Ti_2_NbCNO_2_, the reported experimental or theoretical values of 3.00, 3.14, and 3.04 Å, respectively,^20,21^ are consistent with our findings. However, comparisons must be made cautiously, given that the literature values are for o^*X*^-MXenes, while our work is focused on i^*X*^-MXene phases, which involve different stacking sequences of nonmetal (*X*) layers.

**Table 2:** Lowest Energy MXenes, Average of *a*_0_ and *b*_0_, and *d*_*z*_, in Å^a^

system	*a*_0_	*d*_*z*_	*E*_coh_	*E*_g_	*m*_tot_	*m*_loc_^*M′*^	*m*_loc_^*M″*^	Φ
Mo_2_CO_2_	2.86	5.18	–6.61	metal	NM	NM		7.34
	(2.88 ^53^)			(metal^22,56^ )				(6.75 ^56^)
Cr_2_CO_2_	3.01	4.93	–4.20	0.36	0.00	2.41		6.11
MnNbCO_2_	3.14	4.61	–5.36	metal	16.48	4.26	0.06	5.64
MoVCO_2_	2.92	4.81	–5.44	metal	4.03	0.04	1.18	7.18/6.40
CrMoNO_2_	2.89	4.91	–4.54	0.87	0.00	2.62	0.03	7.42/6.64
MoNbNO_2_	2.89	5.21	–6.71	metal	NM	NM	NM	6.81
NbYBO_2_	3.40	5.21	–6.71	0.08	NM	NM	NM	3.75/4.12
Ti_3_C_2_O_2_	3.02	6.95	–7.36	metal	NM	NM		6.02
	(3.03 ^57^)			(metal^57^)				(6.18 ^58^)
Ti_3_CNO_2_	3.00	6.90	–7.29	metal	NM	NM		5.88
Nb_3_CNO_2_	3.11	7.33	–7.44	metal	NM	NM		5.56
Ti_2_NbCNO_2_	3.04	6.95	–8.07	metal	NM	NM	NM	5.66/5.58
	3.04 ^21^							
V_4_C_3_O_2_	2.96	9.35	–6.03	metal	0.00	0.80		6.30
	(2.95 ^52^)							
Nb_4_C_3_O_2_	3.14	9.79	–7.80	metal	NM	NM		5.55
								(6.39 ^59^)
Cr_4_C_3_O_2_	2.94	9.38	–4.21	metal	32.46	2.35		7.07

a *E*_coh_ in eV/atom, *E*_g_ in eV, and work function (Φ), given in eV. *m*_tot_ is the total magnetic moments in the 2 × 2 supercells, while *m*_loc_ are the metals local magnetic moments normalized by the number of atoms; in units of (μ_B_) and (μ_B_/atom), respectively. For non-symmetric MXenes, we show both values of the Φ according to the metal order, e.g., for (*M′M* *″*)_*n*+1_(*X′X* *″*)_*n*_O_2_, the first (second) value is for the side with *M′* (*M* *″*). Results from literature works are shown in parentheses.

The cohesive energy (*E*_coh_) is a direct measure of the energetic stability of a material, reflecting the strength of the chemical bonding network. It was calculated as follows:

$$E_{\mathrm{coh}}=\frac{E_{\mathrm{tot}}^{i}-\sum_{n=1}^{N_{\mathrm{atoms}}}E_{\mathrm{tot}}^{\mathrm{atoms},n}}{N_{\mathrm{atoms}}}$$ 1

where *E*_tot_^*i*^ is the total energy of MXene and *E*_tot_^atoms, *n*^ are the isolated atomic energies. As expected from chemical bonding principles, thicker MXenes (those with more atomic layers or larger *d*_*z*_) tend to exhibit more negative *E*_coh_, indicating greater thermodynamic stability. This can be rationalized by considering that thicker structures benefit from enhanced metallic bonding interactions, effectively reducing the energy per atom. For example, Ti_2_NbCNO_2_ presents the most negative *E*_coh_ among double metal systems (−8.08 eV/atom), suggesting that it would be particularly resistant to decomposition, while thinner structures such as Cr_2_CO_2_ and CrMoNO_2_ are energetically less stable. This trend is crucial for practical applications; for example, materials with lower stability might be more suitable for defect engineering or surface modifications,^60^ whereas more stable MXenes are promising candidates for robust coatings and electronic devices.

From an electronic perspective, most MXenes exhibit metallic behavior, as indicated by their zero *E*_g_. This is consistent with the delocalized nature of *d*-states in transition metals, which dominate the DOS at the Fermi level. The exception is CrMoNO_2_, which exhibits a moderate *E*_g_ of 0.87 eV, suggesting a potential for semiconducting applications where a small but nonzero *E*_g_ is advantageous. Furthermore, Cr_2_CO_2_ shows a small *E*_g_ (0.36 eV), qualitatively aligning with previous findings using hybrid functionals.^39^ For the three semiconductor systems, we observed the smaller *E*_g_ for NbYBO_2_.

With respect to magnetism properties, electronic filling and hybridization between metal *d*- and oxygen *p*-states play a decisive role. As a general rule, surface functionalization (e.g., with O, OH, or F groups) quenches unpaired electrons and magnetism by saturating dangling bonds and leading to filled or empty *d*-bands.^4^ This is observed in most systems here, which are nonmagnetic. However, some systems exhibit magnetic order: Cr_2_CO_2_, CrMoNO_2_, and V_4_C_3_O_2_ adopt AFM solutions, reflecting incomplete spin compensation between neighboring transition-metal atoms. In particular, Cr_2_CO_2_ is intriguing because DFT-PBE predicts a metallic NM phase,^61,62^ while DFT-HSE calculations favor a semiconductor ferromagnetic (FM) ground state with significant magnetic moments.^39^ In our PBE + *U* calculations, although the AFM configuration is energetically favored, the local magnetic moments (2.41 μ_B_/atom) and *E*_g_ agree well with the HSE values, indicating the robustness of the local moment formation.

Moreover, several double-metal MXenes (MnNbCO_2_, MoVCO_2_, CrMoNO_2_) show significant total magnetic moments (e.g., 16.475 μ_B_ for MnNbCO_2_), primarily localized in one of the transition metals, emphasize the possibility of tailoring magnetic properties by selective metal mixing. This magnetism is particularly promising for spintronics applications.

### 3.5 Structure Stability Analyses via Ligand Field Theory

#### 3.5.1 Nominal Charges

To obtain insights into the relationship of the atomic and electronic structure, we applied LFT. According to the LFT, in fcc-terminated MXenes, the octahedral field around the metal splits the *d*-states into two energy levels, namely, a lower energy *t*_2*g*_-state (*d*_*xy*_, *d*_*yz*_, and *d*_*xz*_) and an antibonding *e*_g_^*^-state (*d*_*z* ^2^_ and *d*_*x* ^2^–*y* ^2^_).^4^ The importance of *d*-states splitting has only been realized recently in the literature, in the systematic theoretical work of Bera.^4^ Other experimental works have discussed the *d*-states splitting, but only octahedral environments were considered.^63,64^ Therefore, we aim to extend the LFT to our MXenes compositions, which include a large variety of structural possibilities, as discussed in the methodology section.

Table 3 shows the nominal charge of metals and the number of nonbonding *d* electrons. Our MXenes follow the expected trends; that is, *d* ^2^ MXenes prefer trigonal prismatic environments, and any other occupancy of *d* leads to octahedral environments. However, for Cr_2_CO_2_, CrMoNO_2_ (Cr side) and Cr_4_C_3_O_2_, our calculations and LFT predictions are different, probably caused by the Hubbard corrections, which change the number of nonbonding states. This analysis is easier for single-metal MXenes than for double-metal MXenes; for example, o^*M*^-MXene MoVCO_2_ prefers a trigonal (octahedral) environment for the Mo (V) sides. In terms of LFT, this can be rationalized if both metals have equal charges, leading to *d* ^2^ and *d* ^1^ configurations. It is important to note that this choice is not unique, which makes the analysis difficult.

**Table 3:** Nominal Charge of Cations (*Q*_n_) and Average DDEC6 Charges (*Q*_av_) of the Lowest Energy MXenes, Given in *e*/Atom^a^

system	*Q*_n_^*M′*^	*Q*_n_^*M″*^	*M′d* ^occ^	*M* *″* *d* ^occ^	structure	*Q*_av_^*M′*^	*Q*_av_^*M″*^	*Q*_av_^*X′*^	*Q*_av_^*X″*^	*Q*_av_^*O*^
Mo_2_CO_2_	4.0		2		hcp	1.15		–1.24		–0.52
Cr_2_CO_2_	4.0		2		fcc	1.55		–1.33		–0.88
MnNbCO_2_	3.0	4.0	4	0	fcc-fcc	1.03	2.19	–1.58		–0.82
MoVCO_2_	4.0	4.0	2	1	hcp-fcc	1.35	1.76	–1.66		–0.73
CrMoNO_2_	3.0	4.0	3	2	hcp-hcp	1.47	1.29		–1.30	–0.73
MoNbNO_2_	4.0	3.0	2	2	hcp-hcp	1.07	1.26		–1.16	–0.59
NbYBO_2_	4.0	3.0	1	0	fcc-fcc	1.55	1.50	–1.29		–0.88
Ti_3_C_2_O_2_	4.0		0		fcc	1.84		–1.82		–0.94
Ti_3_CNO_2_	3.7		1		fcc	1.83		–1.96	–1.65	–0.95
Nb_3_CNO_2_	3.7		4^b^		fcc-fcc	1.50		–1.70	–1.27	–0.77
Ti_2_NbCNO_2_	3.0	5.0	1	0	fcc-fcc	1.86	1.46	–1.73	–1.51	–0.97
V_4_C_3_O_2_	4.0		1		fcc	1.72		–1.70		–0.89
Nb_4_C_3_O_2_	4.0		1		fcc	1.50		–1.49		–0.77
Cr_4_C_3_O_2_	4.0		2		fcc	1.47		–1.41		–0.82

a We considered the general formula (*M′M* *″*)_*n*+1_(*X′X* *″*)_*n*_O_2_. For the nominal charge of oxygen, carbon, nitrogen, and boron, we considered −2, −4, −3, and −3, respectively. The *d*-states occupancy is represented by *d* ^*occ*^ and is the number of non-bonding electrons per atom, following the electron counting rule. The electronic configuration of the free atoms is available in the SI. We used the following terminology in this table: fcc-hcp for (*M′M* *″*)_*n*+1_(*X′X* *″*)_*n*_O_2_ means that the oxygen in *M′* (*M* *″*) side prefers fcc (hcp) site.

b The *d* ^*occ*^ for Nb_3_CNO_2_ is the sum of nonbonding *d* electrons for the three Nb atoms.

#### 3.5.2 Effective Charge Analysis

To proceed further, we calculated electrostatic and chemical (DDEC6) charges, which are shown in Table 3. The DDEC6 analysis, as with other charge analyses, considers the flexibility in determining how to allocate the overall electron distribution among the atoms. In Bader charges, for example, because the electron density is continuous, the minimum in the electronic density between atoms is the point that separates the electronic density of one atom from another.^65^ The DDEC6 analysis has a different approach: (*i*) one electron distribution is assigned for each atom; and (*ii*) core electrons are assigned to the host atom.^66^ The main advantage of this approach is that reference ions are used, which makes the DDEC6 applicable to various materials; e.g., an oxygen anion in any material should resemble the reference ions O^2–^.

All metals are positively charged with values ranging from 1.03 to 2.19 *e*/atom. As expected for carbonitrides and borides, most of the negative charge is in the *X* elements, with values ranging from −1.96 to −1.16 *e*/atom. The oxygen terminations are negatively charged, that is, values ranging from −0.97 to −0.52 *e*/atom, which is due to the smaller coordination of the termination groups in MXenes. Furthermore, we observed that O has more negative charges at the fcc sites, which is explained by the coordination environment, that is, in the fcc sites, O is above the hollow site of the metals. However, for the hcp site, O lies above the electronegative *X* site, which causes a more homogeneous charge displacement.

For all systems, there is no quantitative match between nominal and DDEC6 charges, which is expected due to charge partitioning. For single-metal MXenes, there is qualitative agreement. For MnNbCO_2_, the calculated charge on Mn and Nb are 1.03 and 2.19 *e*/atom, respectively, so it is justifiable to place more positive charges on Nb, in accordance with our nominal charges. The same behavior was observed for NbYBO_2_. However, for systems MoVCO_2_, CrMoNO_2_, MoNbNO_2_, and Ti_2_NbCNO_2_, there is neither quantitative nor qualitative agreement between the nominal charges and the calculated DDEC6 charges. Thus, the LFT can be easily applied to single-metal MXenes to predict the lowest energy structures; however, for double-metal MXenes, it is difficult to correlate the lowest energy structure only from nominal or calculated charges on metals.

#### 3.5.3 Occupation of the *d*-States

To further investigate the role of the *d*_*z* ^2^_-states at the surface O-termination sites, we calculated the integrated projected DOS, that is, we integrated the *d*_*z* ^2^_-states as

$$d_{z^{2}}\mathrm{Occupancy}=\int_{-80\mathrm{eV}}^{E_{\mathrm{Fermi}}}\mathrm{LDOS}(E)dE$$ 2

where *E*_Fermi_ is the Fermi energy and d*E* represents the integration variable, that is, the energy of the states. For semiconductor systems, we used the VBM instead of *E*_Fermi_. LDOS is the local density of states for the *d*_*z* ^2^_-states. We used −80 eV, as it corresponded to the lowest energy state observed in our calculations.

Our results in Figure 5 show that systems with hcp preference have a larger *d*_*z* ^2^_-states occupancies, even for double-metal MXenes. These results extend the LFT to a larger class of materials, i.e., single- and double-metal MXenes, including in- and out-of-plane MXenes. The outliers in Figure 5 are Cr_2_CO_2_, MnNbCO_2_, and Cr_4_C_3_O_2_; these outliers imply that the application of LFT to systems with Hubbard corrections is more complicated. Furthermore, we should emphasize that this analysis is qualitative, since the integration of the LDOS using plane waves as a basis set for DFT calculations does not account for interstitial density due to the projection scheme. The complete results of electronic states occupancies are available in the SI.

**Figure 5:**
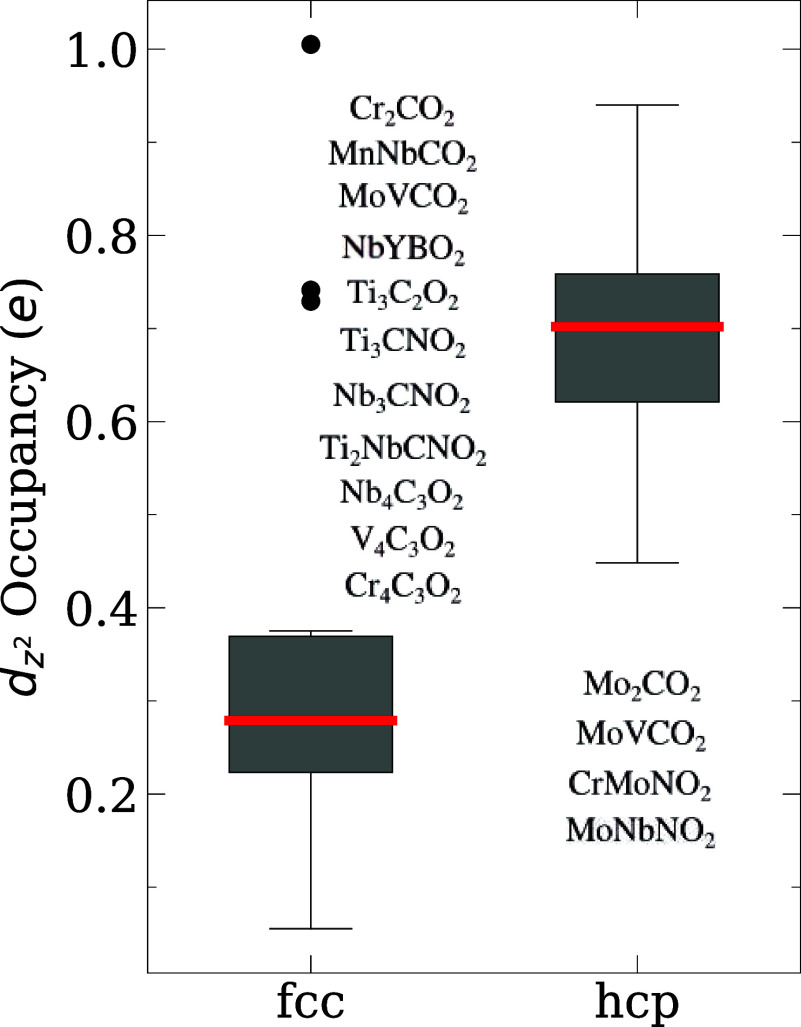
Analysis of *d*_*z* ^2^_-states occupancies calculated via integration of local density of states until the Fermi level for metallic systems or valence band maximum for semiconductor systems. The red lines represent the medians.

### 3.6 Work Function versus Surface Composition

We calculated the work function (Φ) to understand its variation as a function of the composition of MXene. The Φ is orientation dependent and represents the minimum energy required for an electron to escape from a solid:^67^

$$\Phi =V_{\mathrm{es}}(r_{\mathrm{vac}})-E_{\mathrm{Fermi}}$$ 3

where *V*_es_(**r**_vac_) is the electrostatic potential in the middle of the vacuum region of the slab, and *E*_Fermi_ is the Fermi energy. However, for semiconductor systems, we used the valence band maximum (VBM). Table 2 shows the values Φ for all the lowest energy structures, while Figure 6 shows the correlation of Φ and surface area.

**Figure 6:**
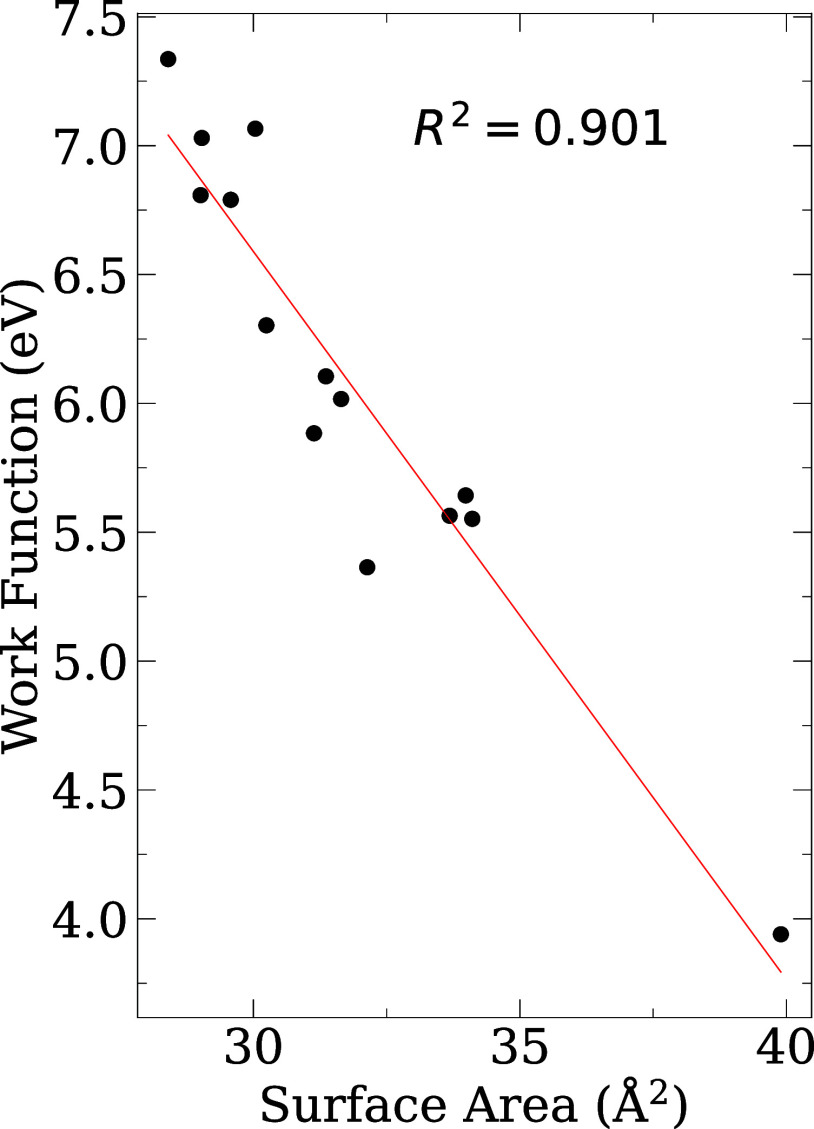
Correlation of work function and surface area. For nonsymmetric MXenes, we considered the average work function for the correlation.

Our Φ values (Table 2 vary considerably among the MXenes. Systems containing Mo, Cr, and V generally have high work functions (Φ > 6.0 eV), associated with strong metallic bonding and dense electronic clouds, while those containing systems Nb or Y exhibit lower values (Φ ∼ 3.75 eV) for NbYBO_2_). In particular, asymmetric MXenes such as MoVCO_2_ and CrMoNO_2_ exhibit two distinct Φ depending on the termination side, which can be used to design devices with tailored directional electron emission or Schottky barriers.

We found that Φ increases with planar density due to the Smoluchowski smoothing phenomenon.^68^ Surface electrons tend to redistribute themselves to smooth out abrupt changes in potential at the atomic scale. This redistribution causes an accumulation of electron density in lower coordinated regions. This smoothing reduces the surface dipole moment, especially on rough or low-density surfaces, leading to a lower work function. Conversely, on smoother or more densely packed surfaces where electron smoothing is less pronounced, the surface dipole remains stronger, resulting in a higher work function. The planar density in atoms/Å^2^ can be calculated as

$$\mathrm{Planar}\;\mathrm{Density}=N_{s}/A_{s}$$ 4

where *N*_s_ is the number of atoms on the surface and *A*_s_ is the area of the surface.

In our case, for all MXenes, the number of atoms on the surface is the same, i.e. four O atoms. The only remaining variable is *A*_s_. The Φ is inversely proportional to the surface area. However, for systems that we used dipole correction, that is, nonsymmetric MXenes, the trend is linear only when we consider the average of Φ. The trend is not perfectly linear because we are comparing surfaces with different compositions. Therefore, the nature of the chemical element also influences the value of Φ, which impacts the value of the coefficient of determination (*R* ^2^), which is not equal to unity.

In the following, we compare our results with other theoretical or experimental data. However, Φ results for our specific MXenes compositions and calculation settings are scarce in the literature. Hence, our comparisons in many cases will be for similar MXenes. For Nb_4_C_3_O_2_, our results do not match the values of the work of Xin and Yu.^59^ In their work, they used a different approach to dispersion corrections. For Nb_2_CO_*x*_, literature results found 5.04 eV; however, the number of layers is different from our work, which makes the comparison difficult.^69^ For Mo_2_CO_2_, the difference between our work and literature may be attributed to different functional.^56,59^ In the case of Ti_3_C_2_O_2_, our calculated value is close to other theoretical works but different from experiments (4.85 eV) due to F terminations; that is, the experimental value is larger due to the high electronegativity of F.^70–72^ Terminations that have electronegativities lower than those of the bare surface decrease the Φ, while those with higher electronegativities have the opposite effect. This phenomenon is interpreted as follows: higher electronegative species harvest electrons from the surface of the material, which reduce the Fermi level.

### 3.7 Electron Localization Function Analysis

To further characterize our MXenes, we investigated the nature of the chemical bond. For that, we calculated the electron localization function (ELF),^73^ which measures the probability density of finding another electron near the reference electron. In this analysis, values closer to unity between atoms indicate complete localization (covalent bonds). Values close to zero between the atoms indicate no localization (ionic bonds). The ELF is calculated using the following equation:

$$\mathrm{ELF}=\frac{1}{1+\chi \left(r\right)}$$ 5

where χ­(**r**) is the ratio of electron localization to the uniform electron gas.

In MXenes, it is known that the metal *t*_2*g*_-states overlap with the *X* and *T p*-states leading to the π-bonds; however, the lower-lying bonding *e*_*g*_-states form strong directional σ-bonds with the *s*- and *p*-states of the *X* and *T* elements.^4,63^ Figure 7 shows that most MXenes have some ionic character, i.e., localization in atoms and smaller localization between the atoms. Some systems are more ionic than others, namely Cr_2_CO_2_, MnNbCO_2_, MoVCO_2_, CrMoNO_2_ and NbYBO_2_, where we observed the localization of electrons in the atoms (ELF≈ 1) and no localization between the atoms (ELF≈ 0), indicating a high ionic character. The other systems have bonds with smaller ionic character.

**Figure 7:**
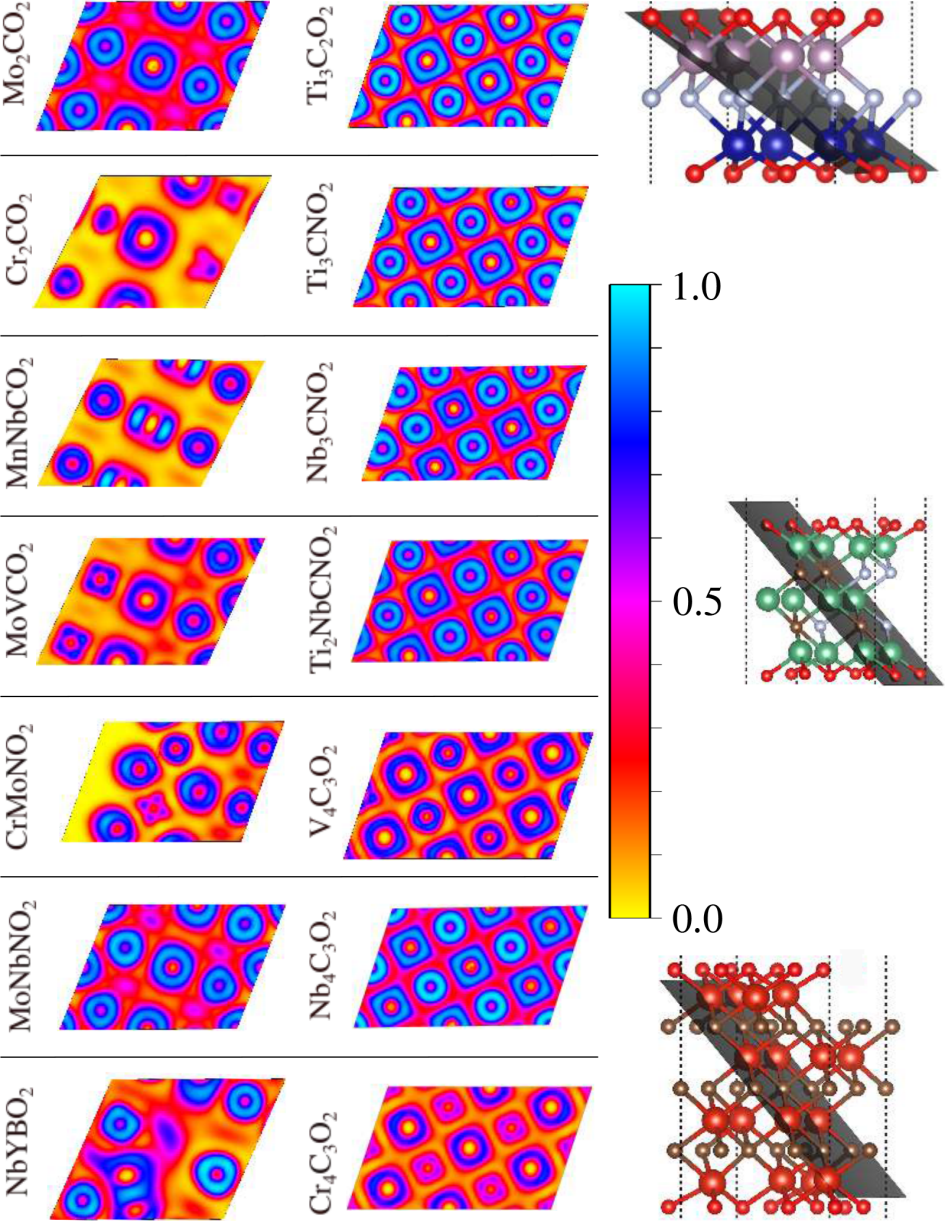
Two-dimensional projection of electron localization function for lowest energy structures. We considered the layer with the maximum number of bonds.

### 3.8 Density of States Characterization

We calculated the DOS of the lowest energy structures to complement the results of band structure calculations (see below). The DOS allows us to observe, for example, which materials can become semiconductors if calculated with the hybrid functional. This analysis is important because metallic materials are more suitable for electrochemical applications.^74^ Before discussing our results, we highlight that the PBE usually underestimates *E*_g_ due to incomplete cancellation of the self-interaction error.^75^ This issue is partially corrected by Hubbard corrections. Regarding the effect of vdW corrections on the *E*_g_ of MXenes, we have one systematic study of Ontiveros et al., which showed that both DFT and DFT-D3 underestimate the *E*_g_ for the MXenes Zr_2_CO_2_, HfCO_2_, ScCO_2_, Y_2_NO_2_, W_2_NO_2_ and Mo_2_NO_2_.^76^ For other materials, other literature works led to the same conclusions; e.g., Torres et al. and Barhoumi and Said demonstrated that the *E*_g_ for TiO_2_, Ti_2_O_3_ and bismuth oxyhalides is underestimated by DFT and DFT-vdW framework.^77,78^

Figure 8 shows that most of our MXenes are metallic. The systems MnNbCO_2_, MoNbNO_2_, Ti_3_C_2_O_2_, and Ti_3_CNO_2_ have a small DOS at or near the Fermi level, which may result in a band gap when calculated with hybrid functionals. The results of the literature show that, with the HSE calculations, Cr_2_CO_2_ is a magnetic semiconductor.^39,79^ The literature reports also show that V_4_C_3_ (no termination group) and Nb_4_C_3_O_2_ are metallic, according to our results.^80,81^ For other systems, we lack experimental or theoretical results for comparison. The other metallic systems exhibit a high DOS near the Fermi level, which suggests that they are likely metallic, even when using other functionals. The *d*-states of the metal contribute predominantly to the DOS near the Fermi level.

**Figure 8:**
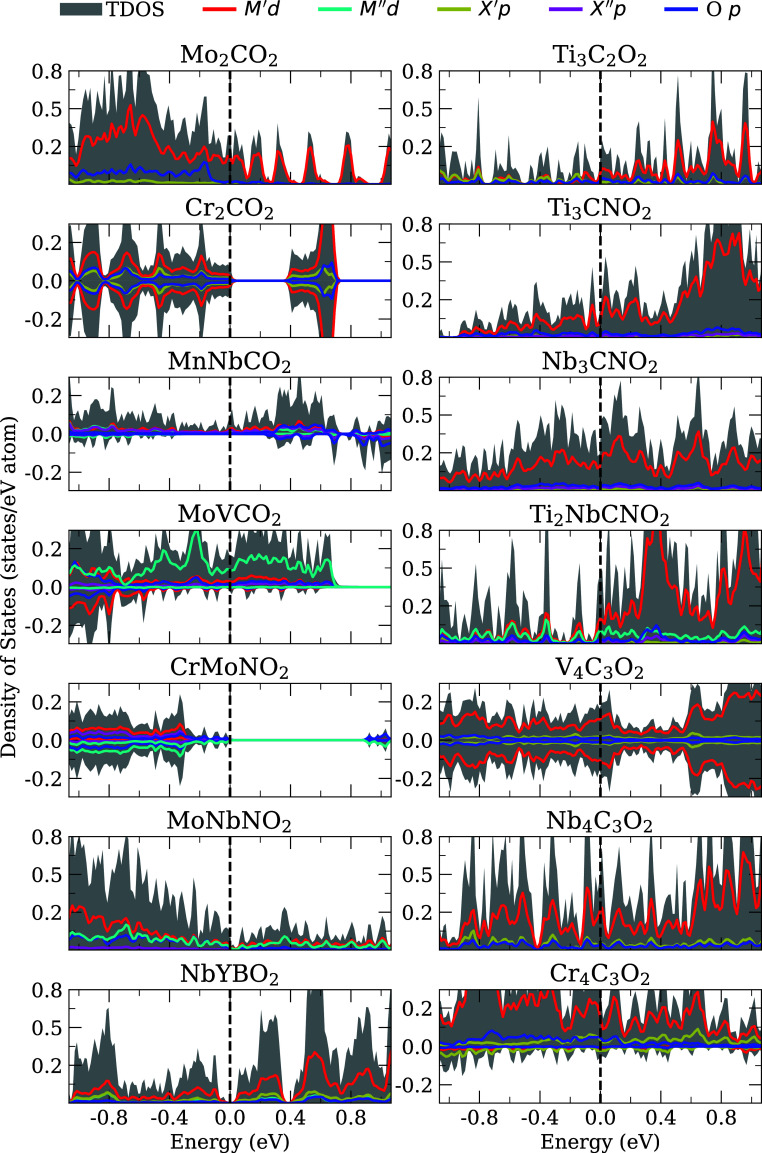
Average total (TDOS) and projected density of states for lowest energy structures. We used a Gaussian broadening of 0.01 eV. The vertical black dashed lines indicate the Fermi level.

### 3.9 Band Structure of Semiconductor MXenes

We calculated the band structure of the lowest energy structures to understand the metallic or semiconductor nature of our MXenes and complement the DOS calculations. Figure 9 shows the results for semiconductor MXenes. Additional results for band structure and DOS are available in the SI. For all compositions, the electronic states contributions show that the largest contribution for the bands comes from the metal *d*-states, then oxygen *p*-states, and finally *p*-states from *X* elements. The majority of our MXenes are metallic with parabolic dispersion near the Fermi level.

**Figure 9:**
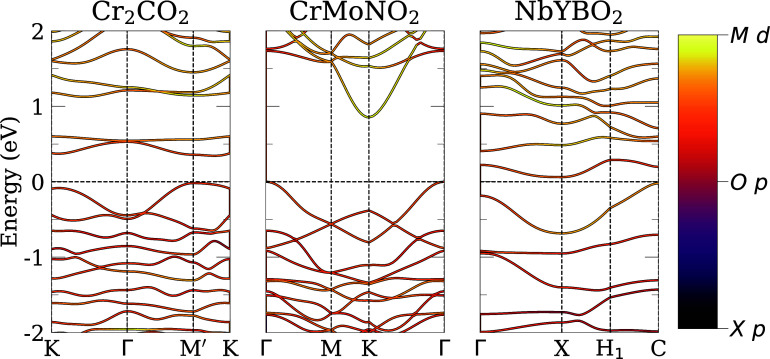
Band structure for the lowest energy structure of semiconductor MXenes. The horizontal black dashed lines indicate the VBM, obtained from a calculation with **k**-mesh doubled. For the color coding, we considered the general formula (*M′M* ^*″*^)_*n*+1_(*X′X* ^*″*^)_*n*_O_2_ and for MXenes with two different metals or *X* elements, we summed the contributions. The paths are in accordance with the work of Wang et al.^82^

The semiconductor systems are Cr_2_CO_2_ (direct *E*_g_), CrMoNO_2_ (indirect), and NbYBO_2_ (indirect). The *E*_g_ is larger for Cr_2_CO_2_ and CrMoNO_2_; see Table 2. The *E*_g_ is directly proportional to the ionic character of the bond between metals and anions. The thermodynamic electronegativity of nitrogen (3.56) is higher than that of boron (3.04), and the sum of the electronegativity of Cr (2.12) and Mo (2.47) is lower than the sum of Nb (2.59) and Y (2.52).^83^ Therefore, the bonds Cr_2_CO_2_ and CrMoNO_2_ are more ionic, resulting in a larger electronic *E*_g_. The metallic system MnNbCO_2_ has a bandgap for one spin component, which opens possibilities for spintronics applications, see SI.

These results are in accordance with the ELF discussion; e.g., most systems have a small ionic character, which results in a metallic nature. For Cr_2_CO_2_ and CrMoNO_2_, bonds are predominantly ionic, which implies a relatively large *E*_g_. For NbYBO_2_, the ionic character is smaller, resulting in a smaller electronic *E*_g_. Note that the bonds in other systems have some ionic character; however, it is not enough to result in a semiconductor nature. Other literature works have shown that the character of the *M*-*X* and *M*-*T* bonds determines the *E*_g_, e.g., the larger the ionic character, the larger the *E*_*g*_. The ionic character is smaller for less electronegative ligands, e.g., C < *N* < O and also decreases with the number of *d*-electrons.^4,63,64^ Metallic interactions occur for long-range *t*_2*g*_ – *t*_2*g*_ overlap, which is larger for metals with many *d* electrons. We should highlight that other mechanisms can be involved in the magnitude of the *E*_g_, e.g., shifting of the Fermi energy to the center of the gap between the *M d*- and *X p*-states due to mixing of these states upon functionalization.^84^

## 4 Insights into MXenes Applications

The values of Φ are an important parameter for practical applications of MXenes in catalysis,^67^ electrochemistry,^74^ photodetection,^85^ and energy conversion.^69^ We observed the lowest Φ values for NbYBO_2_ and Nb_4_C_3_O_2_ and the highest Φ values for Mo_2_CO_2_ and Cr_2_CO_2_. In catalysis, this quantity is important because adsorbates that have electronegativities lower than those of the substrate atoms typically decrease the Φ, while those with higher electronegativities have the opposite effect.^67^ This phenomenon is interpreted as follows: higher electronegative species harvest electrons from the surface of the material, which reduces the Fermi level.

In electrochemistry, MXenes with lower Φ values lose electrons easier and can be used in HER, nitrogen reduction, CO_2_ reduction, or other redox reactions.^74,86^ For example, Gui et al. modified Ti_3_C_2_ *T*_*x*_ with Fe, which greatly reduced the Φ. The MXene/TiFeO_*x*_ achieved a Faradaic efficiency of 25.44% and NH_3_ yield larger than all reported MXene-based materials for nitrogen reduction.^87^ Chen et al. studied Ti_3_C_2_ *T*_*x*_ for photodetection applied to image sensing. In this area, materials with lower Φ are used to collect electrons, and the ones with higher Φ will be used to collect holes.^85^ The authors were able to synthesize Ti_3_C_2_ *T*_*x*_ with few defects, which finally enhanced the efficiency of the optoelectronic device.

In energy conversion, Zhang et al. applied Nb_2_C*T*_*x*_ MXene as the hole transport layer for perovskite solar cells.^69^ Nb_2_C*T*_*x*_ was chosen because it has a larger surface area in comparison with Ti_3_C_2_ *T*_*x*_. In their work, they demonstrated that treating MXene with oxygen plasma increased the number of oxygen terminations, causing an increase in Φ due to the high electronegativity of oxygen. In the case of perovskite solar cells, the increase in Φ is important because it decreases the recombination of charges. This led to an increase in the hole transfer efficiency in the perovskite/MXene interface. Their final material was stable and achieved a power conversion efficiency of 20.74%. The authors also highlight the flexibility of MXenes, which is another important feature of solar cells for flexible devices. In addition, Φ can be further tuned with other terminations.

## 5 Conclusions

In this work, we investigated the compositions of 14 MXenes with a different number of layers using the PBE and PBE + *U* approximations. We extensively explored the structure space, including fcc and hcp terminations, o^*M*^-MXenes, i^*M*^-MXenes, o^*X*^-MXenes, i^*X*^-MXenes, NM, FM, FIM, and AFM configurations. After 240 stress-tensor calculations, we selected the lowest energy structure to calculate cohesive energies, band structures, density of states, work function, nominal charges, electron localization function, and DDEC6 charge analysis.

Our results from PBE calculations showed that most systems are NM for all configurations in the structure space, namely Mo_2_CO_2_, MoNbNO_2_, NbYBO_2_, Ti_3_C_2_O_2_, Ti_3_CNO_2_, Nb_3_CNO_2_, Ti_2_NbCNO_2_, and Nb_4_C_3_O_2_. This is expected because O binds to *d* electrons, which hinders magnetism. Our PBE and PBE + *U* analysis shows that, for systems with Cr, Mn, V the inclusion of *U* leads to important differences. For MnNbCO_2_ and V_4_C_3_O_2_, both functionals agree in terms of *T* site preference, however, there are important differences regarding *a*_0_ and magnetic properties. For Cr_2_CO_2_, CrMoNO_2_, and Cr_4_C_3_O_2_, the differences between both functionals are more critical, that is, even the predicted *T* site changes. For MoVCO_2_, the differences between both functionals are marginal, with only an enhancement of local magnetic moments.

Our results of the cohesive energies showed that most MXenes prefer fcc terminations, while hcp minimized the energy for Mo_2_CO_2_, CrMoNO_2_, MoVCO_2_ (Mo side), and MoNbNO_2_. For double-metal MXenes, in-plane configuration is more stable for MnNbCO_2_, MoNbNO_2_, NbYBO_2_, Ti_3_CNO_2_, and Nb_3_CNO_2_, while the out-of-plane structure minimized the energy for MoVCO_2_, CrMoNO_2_, and Ti_2_NbCNO_2_. Therefore, theoretical calculations, even when focused on specific applications of MXenes, should explore the structural space to identify the lowest energy configuration. This is particularly important for double-metal MXenes, where most of the literature studies assume the structure of o^*M*^-MXene. This assumption is unjustified and may result in the identification of an incorrect energy minimum. The same conclusion is valid for double *X* MXenes. Interestingly, the lowest energy structure of NbYBO_2_ showed an unexpected structure with migration of the boron atom site. These results and the lack of experimental data for MBenes indicate that these materials should be further explored. A similar *X* site migration occurs for Cr_2_CO_2_.

Regarding structural features, the preference for the hcp or fcc termination site is explained by LFT and the number of nonbonding electrons, which is in agreement with the majority of DDEC6 charge analysis. Our analysis of *d*-states occupancy further showed that the *d*_*z* ^2^_ occupancy is correlated with the termination site. However, explaining the configurations of o^*M*^-MXenes, i^*M*^-MXenes, o^*X*^-MXenes, or i^*X*^-MXenes is beyond the scope of LFT, which implies again that structure space exploration is essential for obtaining meaningful theoretical results.

As expected, the majority of our compositions are metallic, which is advantageous for applications of these materials as electrodes, e.g., in electrochemistry applications. In these applications, metals are favorable because there is no bandgap and a large concentration of electrons on the surface. Thus, the application of a potential can result in electron transference to adsorbates more easily than in semiconductors.^74^

For the systems MnNbCO_2_, MoNbNO_2_, Ti_3_C_2_O_2_, and Ti_3_CNO_2_, we observed a small density of states near the Fermi level. Depending on the desired application, the band gap of these two systems should be further investigated theoretically or experimentally. Finally, our calculated work functions are within a range of 3.75–7.42 eV, which is explained by surface area and compositions. We emphasize how information about work functions can be used to guide MXene applications. Therefore, our results can aid experimentalists in practical applications and theoretical scientists in future studies.

## Supplementary Material



## Data Availability

The authors declare no competing financial interest. As mentioned, all DFT calculations were done using the VASP package, which can be used under a nonfree academic license. Furthermore, additional details are provided within the SI, while additional raw data can be obtained directly from the authors upon request. We also provide the data in the Mendeley Data Repository, where it is listed under the same title as this work.

## Notes

Additional technical details, further computational methodologies, in-depth convergence calculations, and complete set of equilibrium lattice parameters and electronic properties for all systems (PDF)

## Abbreviation

DFT: Density functional theory
VASP: Vienna ab initio simulation package
PBE: Perdew–Burke–Ernzerhof
PAW: Projector augmented-wave
