# Supplementary material for: Unveiling Termination Preferences and Screening of Structural Space in Multi-Metal MXenes
Source: ACS Omega. 2025 Jul 15;10(29):32310–25. doi: 10.1021/acsomega.5c04416 (PMC12311674; doi:10.1021/acsomega.5c04416)
Supplement: Supplementary file 1 [file ao5c04416_si_001.pdf]

Electronic Supporting Information: Unveiling Termination Preferences and Screening of Structural Space in Multi-Metal MXenes

Mauricio Mocelim, Henrique A. B. Fonseca, Pedro Ivo R. Moraes, and Juarez L. F. Da Silva*

São Carlos Institute of Chemistry, University of São Paulo, Av. Trabalhador São-Carlense 400, 13560-970*, São Carlos, SP, Brazil*

E-mail: juarez_dasilva@iqsc.usp.br

Contents

S-1 Introduction **S-2**

S-2 Additional Data on the Selected MXene Materials **S-2**

S-3 Additional Computational Details **S-3**

S-4 Computational Convergence Tests **S-5**

S-5 Additional Results – MXenes Properties **S-6**

S-5.1 Equilibrium Lattice Parameters and Electronic Properties S-6

S-5.2 Density of States S-16

S-5.3 Band Structure S-33

S-5.4 Work Function S-48

S-5.5 Electron Density Analysis S-49

S-5.6 Electron Localization Function S-50

References **S-52**

S-1 Introduction

This document provides supplementary computational details that can help in the reproduction of all results. In Section S-2, we examine the materials selected for our investigation. Section S-3 elucidates the specifics of the projector augmented-wave (PAW) method. In Section S-4, we explore computational convergence calculations. The subsequent sections present and analyze complementary findings.

S-2 Additional Data on the Selected MXene Materials

In this work, we are interested in studying the structural, energetic, and electronic properties of MXenes that can be used in the hydrogen evolution reaction (HER). Many of our MXenes structures are double transition metals with the formula $(M'M'')_{n+1}(X'X'')_nO_2$, where M', M'' represent metals and X, X'' is either C, N or B. In our calculations, we considered both out-of-plane double-metal ordered MXenes (o^M-MXene), in-plane double-metal ordered MXenes (i^M-MXene), out-of-plane double X ordered MXenes (o^X-MXene), and in-plane double X ordered MXenes (i^X-MXene).

Table S-1: Selected materials for MXenes structure space exploration. Selected materials for MXenes structure space exploration. The Gibbs free energies of hydrogen adsorption is a descriptor for HER and is is represented by ΔG_{H^*}. Overpotential (η) is for an exchange current density of $10\,\mathrm{mA\,cm^{-2}}$. MXenes have space group $P63/mmc$.[1]

System	ΔG_{H^*} (eV)	η (mV)
Mo_2CO_2[2,3]	−0.01	48
Cr_2CO_2[4]	–	212
$MnNbCO_2$[5]	−0.01	–
$MoVCO_2$[5]	−0.09	–
$CrMoNO_2$[5]	−0.09	–
$MoNbNO_2$[5]	0.09	–
$NbYBO_2$[5]	0.10	–
$Ti_3C_2O_2$[6,7]	−0.21	190
Ti_3CNO_2[8]	−0.12	–
Nb_3CNO_2[8]	−0.10	–
Ti_2NbCNO_2[9]	0.02	–
$V_4C_3O_2$[10]	–	200
$Nb_4C_3O_2$[11,12]	0.20	398
$Cr_4C_3O_2$[13]	0.01	–

S-3 Additional Computational Details

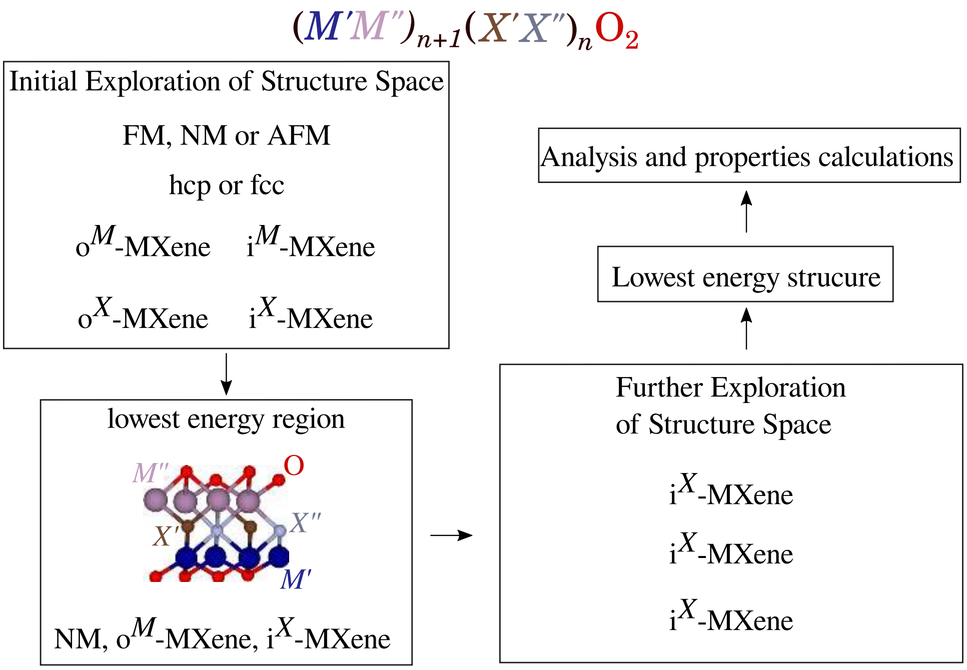

Figure S-1: Summary of the workflow used in this work to explore the structure space of MXenes with the general formula $(M'M'')_{n+1}(X'X'')_nO_2$.

In the Vienna *Ab initio* Simulation Package (VASP), the `INCAR` file encompasses the commands (flags) necessary to execute the calculations. For geometry optimization calculations, we used: `EDIFF` $= 1 \times 10^{-6}$ eV, as a global energy convergence parameter in the self-consistent cycle; `SPIN` $= 2$, for spin-polarized calculations; `ISMEAR` $= 0$ with Gaussian smearing of `SIGMA` $= 0.010$ eV; `ENCUT` $= 1.125 \times$ `ENMAX` was used to calculate properties, where `ENMAX` is recommended in the POTCAR file. The forces acting on the atoms were reduced to $-0.010\,\mathrm{eV\,\AA^{-1}}$ as controlled by `EDIFFG`. For the number of bands, we used `NBANDS` $=$ `number of electrons` and `IVDW` $= 11$ for DFT-D3 method of Grimme with a zero-damping function. For band structure calculations, we used `LCHARG=.TRUE.` in a single-point calculation; then we defined the **k**-path and set `LCHARG=.FALSE.` and `ICHARG=11` for another single-point calculation.

Table S-2 shows the details of the PAW projectors obtained from the VASP library. Standard projectors have a smaller cutoff radius and require a larger number of plane waves to describe the regions near the core. Soft projectors have a larger cutoff radius and therefore require fewer computational resources to describe oscillations near the core.[14] We employed standard PAW projectors because we calculated different compositions, and the use of soft PAW would require a precision assessment for each case. Figure S-1 shows the workflow for this research project.

Table S-2: Technical details of the selected PAW-PBE projectors for this study. Recommended cut-off energy for the plane-wave basis sets (`ENMIN`) and (`ENMAX`), number of valence electrons, (Z_{val}), and valence electronic configuration. Soft projectors are identified by the label *sv*.

Element	PAW projector	`ENMIN` (eV)	`ENMAX` (eV)	Z_{val}	Valence
Mo	Mo_sv_GW_05Dec2013	258.686	344.914	14	$4s^2 4p^6 4d^6$
Cr	Cr_sv_GW_05Dec2013	288.699	384.932	14	$3s^2 3p^6 3d^6$
Mn	Mn_GW_31Mar2010	208.850	278.466	7	$3d^6 4s^1$
Nb	Nb_sv_GW_05Dec2013	156.456	208.608	13	$4s^2 4p^6 4d^5$
Y	Y_sv_GW_05Dec2013	254.818	339.758	11	$4s^2 4p^6 4d^3$
V	V_sv_GW_05Dec2013	286.741	382.321	13	$3s^2 3p^6 3d^5$
Ti	Ti_sv_GW_05Dec2013	287.830	383.774	12	$3s^2 3p^6 3d^4$
B	B_GW_28Sep2005	238.960	318.614	3	$2s^2 2p^1$
C	C_GW_new_19Mar2012	310.494	413.992	4	$2s^2 2p^2$
N	N_GW_new_19Mar2012	315.677	420.902	5	$2s^2 2p^3$
O	O_GW_new_19Mar2012	325.824	434.431	6	$2s^2 2p^4$

S-4 Computational Convergence Tests

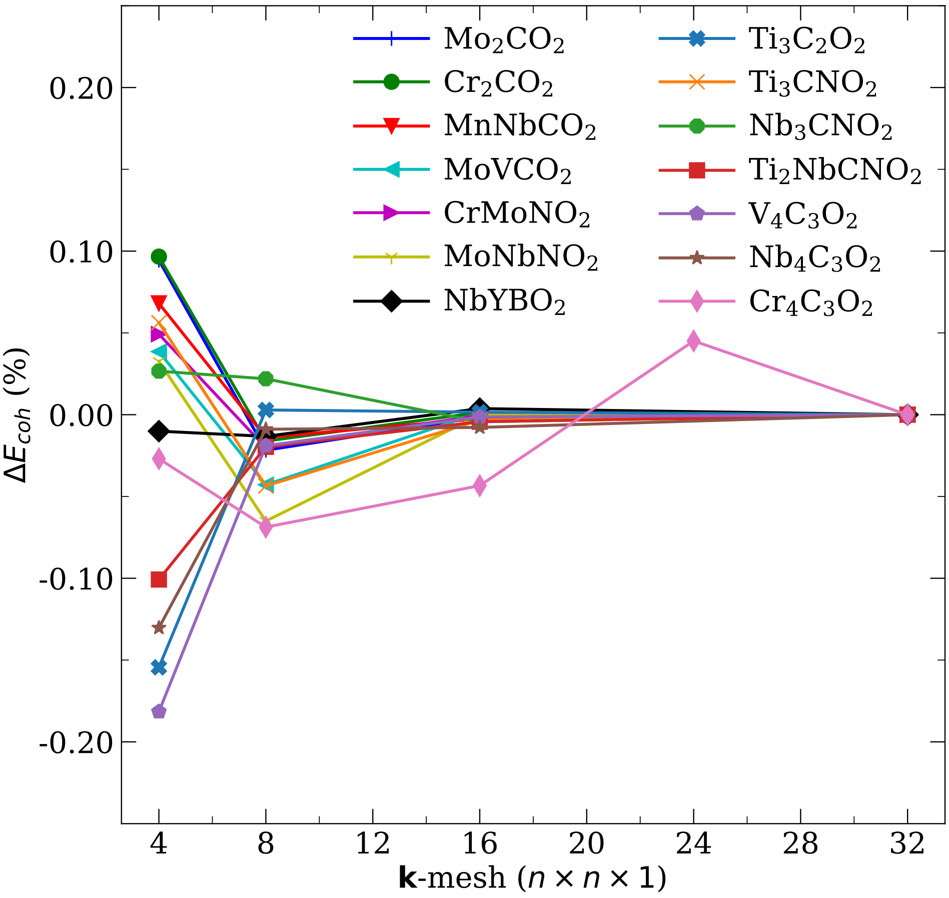

Figure S-2: MXene convergence calculations for cohesive energies (E_{coh}). The Δ was calculated considering the structure with the largest **k**-mesh as a reference.

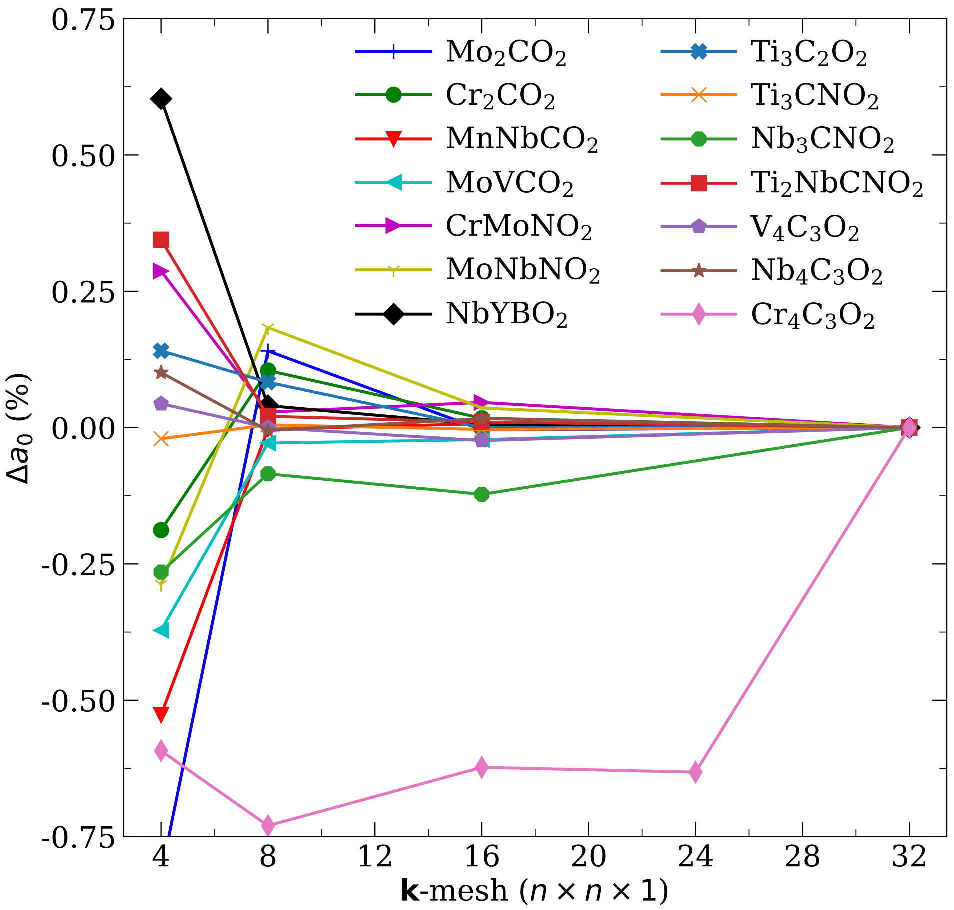

Figure S-3: MXene convergence calculations for lattice parameter (a_0). The Δ was calculated considering the structure with the largest **k**-mesh as a reference.

Figures S-2–S-3 show the results of computational convergence tests for 1×1 cells. Our results show that, for cohesive energies (E_{coh}), if we consider an error of 1 %, all systems are converged even with a **k**-mesh of $4 \times 4 \times 1$. Regarding a_0 convergence, considering an error up to 0.75 %, a **k**-mesh of $4{\times}4{\times}1$ is enough to yield accurate results for Cr_2CO_2, $MoVCO_2$, $CrMoNO_2$, $MoNbNO_2$, $Ti_3C_2O_2$, Ti_3CNO_2, Nb_3CNO_2, Ti_2NbCNO_2, $V_4C_3O_2$, $Nb_4C_3O_2$, and $Cr_4C_3O_2$, while it is increased for $8{\times}8{\times}1$ **k**-mesh for Mo_2CO_2, $MnNbCO_2$, and $NbYBO_2$.

S-5 Additional Results – MXenes Properties

S-5.1 Equilibrium Lattice Parameters and Electronic Properties

Table S-3 shows all calculations for the exploration of the structure space of MXenes. We ordered the structures according to the relative total energies but reported several energetic, electronic,

and structural properties for MXenes. Our results show that fcc terminations minimize energy in most cases; however, for MXenes with different compositions, the exploration of structure space is essential, e.g., the lowest energy structure of $CrMoNO_2$ is an AFM, and many MXenes prefer in-plane configurations. For further discussion, see the manuscript. Table S-4–S-6 shows the results for PBE+U calculations. Our results indicate that, for systems without Cr, Mn or V, trends are consistent; that is, PBE and PBE+U agree qualitatively, including energetic and magnetic properties and bandgap. However, for systems with Cr, Mn and V we explored the structure space with PBE+U, as detailed in the manuscript.

For Cr_2CO_2, the results available in the literature using HSE functional are for hcp terminations. Therefore, comparison should be careful.[15] If we consider that the termination does not affect the magnetic properties, then the PBE+U correctly describes the local magnetic moments in Cr_2CO_2. For PBE, the magnetic and NM systems are degenerate. Overall, our PBE+U results for this system are in accordance with previous PBE+U calculations, but we found a AFM lowest energy structure.[16] For Mo_2CO_2, we do not have hybrid calculations to compare; however, the results for PBE and PBE+U are consistent.

Table S-3: Summary of MXenes properties for PBE structure space exploration. In the configuration column we represent out-of-plane and in-plane MXenes by o- and i-. We also show oxygen site preference, i.e., hexagonal close packed (hcp) or face-centered cubic (fcc), e.g., fcc-hcp for $MnNbCO_2$ means that the oxygen in Mn (Nb) side prefers fcc (hcp). Ferromagnetic, antiferromagnetic, ferrimagnetic, and non-magnetic are represented by FM, AFM, FIM, and NM, respectively. Cohesive energies (E_{coh}), relative total energies (ΔE_{tot}) and lattice parameters (a_0, b_0) are given in (eV/atom), (meV/atom) and (Å), respectively. a_0^L is the lattice parameter from previous literature works in (Å) and d_z is the MXene thickness. For each composition, the lowest energy structure is at the bottom. m_{tot} and m_{loc} are the total and modulus of metal local magnetic moments in units of (μ_B) and (μ_B/atom), respectively. AFM configurations are labeled with a number; however, we reported the final magnetic configuration. Different i-MXenes structures were labeled with capital letters. We considered the general formula $(M'M'')_{n+1}(X'X'')_nO_2$.

System	Final Conf.	E_{coh}	ΔE_{tot}	a_0	b_0	a_0^L	d_z	m_{tot}	$m_{loc}^{M'}$	$m_{loc}^{M''}$
Mo_2CO_2	fcc-NM	−6.384	228	3.084	3.084	–	4.48	–	–	–
	hcp-NM	−6.611	0	2.860	2.866	2.880[17]	5.18	–	–	–
Cr_2CO_2	fcc-FIM-1	−5.489	62	2.859	2.884	–	4.40	1.50	1.55	–
	fcc-AFM-7	−5.493	58	2.873	2.849	–	4.51	0.00	1.44	–
	fcc-AFM-3	−5.494	57	2.847	2.822	–	4.52	0.00	1.15	–
	fcc-FIM-8	−5.495	56	2.873	2.828	–	4.52	0.50	1.36	–

Continued on next page

	fcc-FIM-6	−5.495	55	2.846	2.826	–	4.49	1.00	1.17	–
	fcc-FIM-9	−5.496	55	2.882	2.823	–	4.56	1.50	1.40	–
	fcc-AFM-4	−5.498	53	2.830	2.833	–	4.53	0.00	1.26	–
	fcc-FIM-2	−5.499	51	2.833	2.845	–	4.47	2.00	1.41	–
	fcc-AFM-5	−5.502	49	2.824	2.826	–	4.51	0.00	1.45	–
	fcc-FIM	−5.509	41	2.856	2.841	–	4.50	10.23	1.35	–
	hcp-FIM-6	−5.549	1	2.663	2.689	–	4.81	0.50	0.16	–
	hcp-AFM-9	−5.550	1	2.664	2.679	–	4.83	0.00	0.13	–
	hcp-FIM	−5.550	1	2.671	2.680	–	4.82	1.34	0.19	–
	hcp-AFM-3	−5.550	1	2.674	2.675	–	4.83	0.00	0.22	–
	hcp-AFM-4	−5.550	0	2.694	2.663	–	4.83	0.00	0.11	–
	hcp-FIM-7	−5.550	0	2.678	2.678	–	4.82	1.00	0.25	–
	hcp-NM-2	−5.550	0	2.667	2.667	–	4.85	–	–	–
	hcp-NM-1	−5.550	0	2.668	2.667	–	4.85	–	–	–
	hcp-NM-5	−5.550	0	2.668	2.668	–	4.85	–	–	–
	hcp-NM-8	−5.550	0	2.668	2.668	–	4.85	–	–	–
$MnNbCO_2$	i-H-hcp-hcp-NM	−5.745	632	2.565	2.556	–	6.19	–	–	–
	i-D-hcp-hcp-NM	−5.745	632	2.564	2.556	–	6.20	–	–	–
	i-A-hcp-hcp-NM	−5.745	632	2.564	2.557	–	6.19	–	–	–
	i-G-hcp-hcp-NM	−5.745	632	2.562	2.557	–	6.20	–	–	–
	i-E-hcp-hcp-NM	−5.745	632	2.556	2.561	–	6.19	–	–	–
	i-B-hcp-hcp-NM	−5.745	632	2.560	2.560	–	6.19	–	–	–
	i-F-hcp-hcp-NM	−5.745	632	2.559	2.560	–	6.19	–	–	–
	i-C-hcp-hcp-NM	−5.745	632	2.548	2.563	–	6.20	–	–	–
	o-hcp-hcp-FIM	−6.071	306	2.913	2.913	–	4.79	7.42	1.61	0.33
	o-hcp-hcp-FIM-1	−6.071	306	2.894	2.935	–	4.83	7.42	1.61	0.33
	o-hcp-fcc-FIM	−6.121	256	3.017	3.021	–	4.46	7.81	1.96	0.05
	o-hcp-fcc-FIM-1	−6.188	190	3.078	3.060	–	4.60	1.96	1.20	0.02
	i-J-fcc-fcc-FIM	−6.208	170	3.198	2.940	–	5.15	9.38	2.36	0.04
	o-fcc-hcp-AFM-1	−6.220	157	2.966	2.955	–	4.68	0.00	2.53	0.12
	o-fcc-hcp-FIM	−6.227	151	2.966	2.967	–	4.46	9.43	2.71	0.11
	i-G-hcp-fcc-FIM	−6.235	142	3.066	3.020	–	4.82	8.32	2.04	0.04
	i-H-hcp-fcc-FIM-1	−6.248	129	2.990	2.990	–	4.65	2.07	0.58	0.04
	i-H-hcp-fcc-FIM	−6.248	129	2.990	2.990	–	4.65	2.07	0.58	0.04
	o-fcc-fcc-AFM-1	−6.249	128	3.007	3.005	–	4.50	0.00	0.01	2.42
	fcc-fcc-FIM-o	−6.264	113	3.031	3.030	–	4.39	8.19	2.34	0.24
	i-G-fcc-hcp-FIM	−6.289	89	2.946	2.944	–	5.05	8.38	2.15	0.09
	i-H-fcc-hcp-FIM-1	−6.309	69	2.931	2.931	–	4.83	2.89	1.97	0.07
	i-H-fcc-hcp-FIM	−6.312	65	2.938	2.938	–	4.81	8.82	2.25	0.06
	i-H-fcc-fcc-FIM-1	−6.328	49	3.018	3.006	–	4.59	3.48	1.91	0.04
	i-E-fcc-fcc-FIM	−6.338	40	3.019	3.039	–	4.60	7.86	2.61	0.03
	i-G-fcc-fcc-FIM	−6.339	39	3.036	3.019	–	4.59	10.15	2.61	0.11
	i-H-fcc-fcc-FIM	−6.348	29	3.010	3.008	–	4.55	8.14	2.22	0.16
	i-F-fcc-fcc-FIM	−6.377	0	3.023	2.987	–	4.55	8.11	2.19	0.11
$MoVCO_2$	o-J-hcp-fcc-FIM	−6.676	270	3.120	2.917	–	5.47	2.11	0.04	0.56
	o-fcc-hcp-FIM-1	−6.756	189	2.903	2.905	–	4.79	0.02	0.02	0.01
	o-fcc-hcp-FIM	−6.757	189	2.902	2.906	–	4.89	0.40	0.00	0.00
	i-H-fcc-hcp-NM	−6.785	161	2.905	2.901	–	4.97	–	–	–

Continued on next page

	i-G-fcc-hcp-NM	−6.791	155	2.902	2.906	–	4.89	–	–	–
	o-fcc-fcc-FIM	−6.848	97	2.965	2.958	–	4.74	0.42	0.04	0.00
	o-fcc-fcc-FIM-1	−6.848	97	2.965	2.958	–	4.74	0.42	0.04	0.01
	i-H-fcc-fcc-NM	−6.855	90	2.975	2.985	–	4.71	–	–	–
	i-G-fcc-fcc-NM	−6.856	90	2.992	2.976	–	4.71	–	–	–
	i-H-hcp-hcp-NM	−6.867	79	2.839	2.839	–	4.99	–	–	–
	i-G-hcp-hcp-NM	−6.867	79	2.839	2.839	–	4.99	–	–	–
	o-hcp-hcp-FIM-1	−6.871	75	2.832	2.853	–	4.93	0.41	0.03	0.02
	o-hcp-hcp-FIM	−6.874	71	2.842	2.844	–	4.96	2.09	0.04	0.06
	i-G-hcp-fcc-FIM-1	−6.878	68	2.897	2.890	–	4.88	0.43	0.02	0.10
	i-H-hcp-fcc-FIM-1	−6.883	62	2.876	2.898	–	4.89	2.13	0.10	0.43
	i-G-hcp-fcc-FIM	−6.886	59	2.898	2.897	–	4.84	3.84	0.20	0.01
	i-H-hcp-fcc-FIM	−6.888	58	2.899	2.898	–	4.86	3.84	0.19	0.01
	o-hcp-fcc-FIM-2	−6.932	14	2.893	2.892	–	4.78	0.43	0.04	0.64
	o-hcp-fcc-FIM-1	−6.933	13	2.898	2.894	–	4.79	1.29	0.04	0.01
	o-hcp-fcc-FIM-3	−6.946	0	2.892	2.892	–	4.80	3.90	0.04	0.92
	o-hcp-fcc-FIM	−6.946	0	2.892	2.892	–	4.80	3.90	0.08	0.00
$CrMoNO_2$	o-J-fcc-hcp-FM	−5.548	302	2.912	2.990	–	5.09	5.86	1.70	0.09
	o-hcp-fcc-AFM-1	−5.601	249	2.773	2.761	–	5.26	0.00	0.53	0.02
	o-hcp-fcc-FIM	−5.603	247	2.767	2.760	–	5.30	0.90	0.20	0.07
	i-H-hcp-fcc-NM	−5.649	201	2.769	2.764	–	5.35	–	–	–
	i-G-hcp-fcc-NM	−5.652	197	2.768	2.765	–	5.34	–	–	–
	i-G-fcc-fcc-NM-1	−5.657	192	2.863	2.895	–	5.00	–	–	–
	i-H-fcc-fcc-AFM-1	−5.674	175	2.860	2.851	–	5.30	0.00	0.01	0.00
	i-H-fcc-hcp-AFM-1	−5.728	122	2.803	2.794	–	5.25	0.00	0.01	0.00
	i-H-fcc-hcp-NM	−5.728	122	2.803	2.794	–	5.25	–	–	–
	o-hcp-hcp-NM	−5.734	116	2.769	2.769	–	5.02	–	–	–
	i-H-fcc-fcc-FIM	−5.735	114	2.947	2.939	–	4.90	9.14	2.29	0.17
	i-G-fcc-fcc-FIM	−5.735	114	2.947	2.939	–	4.90	9.14	2.29	0.18
	i-G-fcc-hcp-FIM	−5.738	112	2.824	2.814	–	5.22	4.63	1.28	0.12
	o-fcc-fcc-AFM-1	−5.740	110	2.905	2.920	–	4.87	0.00	2.25	0.01
	o-fcc-fcc-FIM	−5.749	100	2.918	2.926	–	4.87	9.98	2.42	0.05
	i-G-hcp-hcp-NM	−5.751	99	2.763	2.762	–	5.08	–	–	–
	i-H-hcp-hcp-NM	−5.751	99	2.763	2.762	–	5.08	–	–	–
	o-fcc-hcp-FIM-3	−5.756	94	2.839	2.838	–	5.13	5.46	1.31	0.06
	o-hcp-hcp-NM-9	−5.787	62	2.902	2.895	–	5.08	0.10	1.71	0.02
	o-fcc-hcp-FIM-7	−5.845	5	2.868	2.868	–	4.86	5.41	2.43	0.05
	o-fcc-hcp-AFM-9	−5.846	4	2.857	2.869	–	4.85	0.00	2.38	0.02
	o-fcc-hcp-AFM-2	−5.846	4	2.858	2.869	–	4.85	0.00	2.38	0.02
	o-fcc-hcp-AFM-8	−5.846	4	2.857	2.869	–	4.85	0.00	2.38	0.02
	o-fcc-hcp-AFM-5	−5.846	4	2.858	2.870	–	4.85	0.00	2.38	0.02
	o-fcc-hcp-AFM-1	−5.846	4	2.857	2.869	–	4.85	0.00	2.38	0.02
	o-fcc-hcp-AFM-4	−5.846	4	2.858	2.870	–	4.85	0.00	2.38	0.02
	o-fcc-hcp-AFM-6	−5.846	4	2.858	2.870	–	4.85	0.00	2.38	0.02
	o-fcc-hcp-FM	−5.850	0	2.872	2.872	–	4.85	10.82	2.58	0.08
$MoNbNO_2$	i-J-hcp-hcp-NM	−6.434	277	3.042	3.055	–	5.26	–	–	–
	o-hcp-fcc-NM	−6.509	202	2.914	2.883	–	5.29	–	–	–
	o-fcc-hcp-NM	−6.522	190	2.965	2.934	–	5.06	–	–	–

Continued on next page

	o-fcc-fcc-NM	−6.548	164	3.105	3.105	–	4.45	–	–	–
	i-G-hcp-fcc-NM	−6.616	96	2.938	2.910	–	5.44	–	–	–
	i-H-hcp-fcc-NM	−6.616	96	2.938	2.910	–	5.44	–	–	–
	i-G-fcc-fcc-NM	−6.622	90	3.108	3.079	–	4.71	–	–	–
	i-H-fcc-fcc-NM	−6.622	90	3.108	3.080	–	4.71	–	–	–
	o-hcp-hcp-NM	−6.628	83	2.908	2.888	–	5.22	–	–	–
	i-H-fcc-hcp-NM	−6.630	82	2.923	2.941	–	5.30	–	–	–
	i-G-fcc-hcp-NM	−6.631	81	2.974	2.930	–	5.37	–	–	–
	i-H-hcp-hcp-NM	−6.689	23	2.906	2.886	–	5.23	–	–	–
	i-G-hcp-hcp-NM	−6.689	23	2.906	2.887	–	5.23	–	–	–
	i-A-hcp-hcp-NM	−6.707	5	2.883	2.898	–	5.22	–	–	–
	i-C-hcp-hcp-NM	−6.708	3	2.911	2.885	–	5.22	–	–	–
	i-B-hcp-hcp-NM	−6.712	0	2.886	2.898	–	5.21	–	–	–
$NbYBO_2$	o-hcp-fcc-NM	−6.414	294	3.389	3.349	–	4.90	–	–	–
	o-fcc-fcc-NM	−6.448	260	3.359	3.359	–	4.92	–	–	–
	i-G-fcc-hcp-NM	−6.451	257	3.533	3.526	–	5.34	–	–	–
	o-fcc-hcp-NM	−6.472	235	3.513	3.519	–	4.30	–	–	–
	o-hcp-hcp-NM	−6.484	223	3.513	3.503	–	4.34	–	–	–
	i-H-hcp-hcp-NM	−6.500	208	3.511	3.512	–	4.46	–	–	–
	i-H-fcc-hcp-NM	−6.519	189	3.466	3.474	–	5.22	–	–	–
	i-H-hcp-fcc-NM	−6.540	167	3.475	3.384	–	5.00	–	–	–
	i-G-hcp-fcc-NM	−6.532	175	3.301	3.341	–	4.34	–	–	–
	i-I-fcc-fcc-NM	−6.561	146	3.508	3.505	–	5.21	–	–	–
	i-G-hcp-hcp-NM	−6.569	138	3.572	3.527	–	4.79	–	–	–
	i-B-fcc-fcc-NM	−6.592	116	3.338	3.331	–	5.10	–	–	–
	i-A-fcc-fcc-NM	−6.598	110	3.324	3.307	–	5.16	–	–	–
	i-C-fcc-fcc-NM	−6.598	110	3.301	3.324	–	5.17	–	–	–
	i-H-fcc-fcc-NM	−6.658	50	3.468	3.420	–	5.32	–	–	–
	i-J-fcc-fcc-NM	−6.699	9	3.436	3.373	–	6.40	–	–	–
	i-G-fcc-fcc-NM	−6.707	0	3.436	3.373	–	5.21	–	–	–
$Ti_3C_2O_2$	hcp-NM	−7.131	226	2.999	3.011	–	7.13	–	–	–
	fcc-NM	−7.357	0	3.018	3.022	3.030[18]	6.95	–	–	–
Ti_3CNO_2	o-hcp-hcp-NM	−7.069	222	2.951	2.966	–	7.21	–	–	–
	i-G-hcp-hcp-NM	−7.072	220	2.952	2.969	–	7.24	–	–	–
	i-H-hcp-hcp-NM	−7.074	217	2.974	2.956	–	7.25	–	–	–
	o-fcc-hcp-NM	−7.173	118	2.976	2.983	–	7.05	–	–	–
	o-hcp-fcc-NM	−7.174	118	2.990	2.975	–	7.09	–	–	–
	i-H-hcp-fcc-NM	−7.181	111	2.992	2.976	–	7.09	–	–	–
	i-G-hcp-fcc-NM	−7.181	110	2.993	2.976	–	7.08	–	–	–
	i-G-fcc-hcp-NM	−7.181	110	2.977	2.984	–	7.09	–	–	–
	i-H-fcc-hcp-NM	−7.182	109	2.993	2.977	–	7.08	–	–	–
	o-fcc-fcc-NM	−7.273	19	2.997	2.996	3.004[8]	6.91	–	–	–
	i-H-fcc-fcc-NM	−7.287	4	2.993	2.999	–	6.91	–	–	–
	i-G-fcc-fcc-NM	−7.288	3	2.997	2.997	–	6.91	–	–	–
	i-B-fcc-fcc-NM	−7.290	1	2.994	2.999	–	6.91	–	–	–
	i-C-fcc-fcc-NM	−7.291	1	2.994	2.999	–	6.91	–	–	–
	i-A-fcc-fcc-NM	−7.291	0	2.996	2.999	–	6.90	–	–	–

Continued on next page

Nb_3CNO_2	o-hcp-fcc-NM	−7.370	69	3.080	3.080	–	7.41	–	–	–
	i-G-fcc-hcp-NM	−7.402	36	3.079	3.087	–	7.60	–	–	–
	i-H-fcc-hcp-NM	−7.407	32	3.086	3.086	–	7.60	–	–	–
	o-fcc-fcc-NM	−7.411	27	3.110	3.110	–	7.26	–	–	–
	o-hcp-hcp-NM	−7.420	18	3.048	3.047	–	7.56	–	–	–
	i-G-hcp-hcp-NM	−7.420	18	3.047	3.047	–	7.57	–	–	–
	i-H-fcc-fcc-NM	−7.422	17	3.109	3.109	–	7.28	–	–	–
	i-H-hcp-fcc-NM	−7.422	16	3.075	3.069	–	7.44	–	–	–
	o-fcc-hcp-NM	−7.422	16	3.068	3.058	3.136[8]	7.49	–	–	–
	i-H-hcp-hcp-NM	−7.423	15	3.048	3.048	–	7.57	–	–	–
	i-B-fcc-fcc-NM	−7.422	16	3.118	3.113	–	7.32	–	–	–
	i-G-hcp-fcc-NM	−7.426	12	3.072	3.070	–	7.44	–	–	–
	i-G-fcc-fcc-NM	−7.430	9	3.122	3.104	–	7.32	–	–	–
	i-A-fcc-fcc-NM	−7.430	8	3.120	3.117	–	7.29	–	–	–
	i-C-fcc-fcc-NM	−7.438	0	3.110	3.115	–	7.33	–	–	–
Ti_2NbCNO_2	o-hcp-hcp-NM	−7.875	200	2.999	2.997	–	7.34	–	–	–
	i-G-hcp-hcp-NM	−7.920	155	2.997	3.007	–	7.38	–	–	–
	i-H-hcp-hcp-NM	−7.921	154	2.983	2.985	–	7.37	–	–	–
	o-fcc-hcp-NM	−7.972	103	3.033	3.037	–	7.06	–	–	–
	i-G-fcc-hcp-NM	−7.975	99	3.029	3.036	–	7.18	–	–	–
	i-H-fcc-hcp-NM	−7.977	97	3.031	3.023	–	7.20	–	–	–
	o-hcp-fcc-NM	−7.995	80	3.026	3.008	–	7.16	–	–	–
	i-H-hcp-fcc-NM	−8.006	69	3.018	3.012	–	7.20	–	–	–
	i-G-hcp-fcc-NM	−8.032	42	3.012	3.027	–	7.15	–	–	–
	i-H-fcc-fcc-NM	−8.039	35	3.060	3.037	–	7.09	–	–	–
	i-G-fcc-fcc-NM	−8.064	10	3.064	3.041	–	6.97	–	–	–
	o-fcc-fcc-NM	−8.075	0	3.036	3.046	3.040[9]	6.95	–	–	–
$V_4C_3O_2$	hcp-NM-1	−7.561	53	2.873	2.873	–	9.41	–	–	–
	hcp-NM-2	−7.561	53	2.908	2.900	–	9.41	–	–	–
	hcp-FIM	−7.561	53	2.873	2.873	–	9.40	0.45	0.03	–
	fcc-FIM	−7.612	3	2.908	2.900	–	9.24	3.01	0.21	–
	fcc-NM-1	−7.614	1	2.907	2.905	–	9.24	–	–	–
	fcc-NM	−7.614	1	2.906	2.905	–	9.23	–	–	–
	fcc-FIM-2	−7.615	0	2.906	2.908	–	9.23	0.01	0.03	–
	fcc-FIM-5	−7.615	0	2.908	2.906	–	9.23	0.01	0.03	–
	fcc-FIM-3	−7.615	0	2.908	2.906	–	9.23	0.01	0.03	–
	fcc-FIM-7	−7.615	0	2.908	2.906	–	9.23	0.01	0.03	–
	fcc-FIM-4	−7.615	0	2.908	2.906	–	9.23	0.43	0.04	–
	fcc-FIM-6	−7.615	0	2.909	2.907	–	9.23	0.43	0.04	–
	fcc-NM-8	−7.615	0	2.909	2.907	2.950[19]	9.23	–	–	–
$Nb_4C_3O_2$	hcp-NM	−7.763	36	3.096	3.097	–	10.08	–	–	–
	fcc-NM	−7.799	0	3.135	3.138	–	9.79	–	–	–
$Cr_4C_3O_2$	fcc-NM-1	−5.715	42	2.823	2.823	–	9.14	0.00	0.00	–
	fcc-AFM-2	−5.718	39	2.824	2.824	–	9.16	0.00	0.46	–
	fcc-FIM	−5.752	5	2.856	2.856	–	9.14	15.01	1.00	–
	hcp-AFM-4	−5.753	5	2.765	2.765	–	9.48	0.00	0.03	–
	hcp-AFM-3	−5.753	5	2.765	2.765	–	9.48	0.00	0.03	–

Continued on next page

hcp-NM-1	−5.753	5	2.765	2.765	–	9.49	0.00	0.00	–
hcp-FIM-6	−5.753	4	2.760	2.755	–	9.60	4.83	0.55	–
hcp-AFM-5	−5.757	0	2.767	2.767	–	9.50	0.00	0.45	–
hcp-AFM	−5.757	0	2.767	2.767	–	9.50	0.00	0.45	–
hcp-AFM-2	−5.757	0	2.767	2.767	–	9.50	0.00	0.45	–

Table S-4: Comparison of properties for different functionals, that is, PBE, PBE+U, and HSE. The $m_{loc}^{M'}$ values are given in μ_B/atom. This table was constructed based on results of previous literature works.[15,16] E_g, a_0, and d_z are given in eV, Å, and Å, respectively. The PBE and PBE+U from this work are marked with *. For Mo_2CO_2, our PBE+U results are for a single-point PBE lowest energy structure. HSE results for Cr_2CO_2 are only available for hcp termination.

System	Property	PBE*	PBE+U*	PBE+U[16]	HSE[15]
Cr_2CO_2	$m_{loc}^{M'}$	NM	2.41 (AFM)	2.57 (FM)	2.35 (FM)
	E_g	metal	0.36	metal	~ 0.5
	a_0	2.67	3.05	–	–
	d_z	4.85	4.93	–	–
	Termination	hcp	fcc	fcc	hcp
Mo_2CO_2	$m_{loc}^{M'}$	NM	NM	NM	–
	E_g	metal	metal	metal	–
	a_0	2.86	2.86	2.89	–
	d_z	5.18	5.18	5.26	–
	Termination	hcp	hcp	hcp	–

Table S-5: Summary of MXenes properties for PBE and PBE+U structure space exploration. We used the PBE lowest energy structure for a single point spin-polarized PBE and PBE+U calculation; see Table S-3. For PBE+U calculations, we considered different structures within a range of 100 meV from PBE calculations, including AFM and FM configurations. E_{tot} are given in (eV/atom). m_{tot} and m_{loc} are in units of (μ_B) and (μ_B/atom), respectively. E_g is the bandgap energy. 'sp' and 'opt' stand for single-point and optimization calculations. 'with' and 'without' stand for spin polarized and unpolarized calculations. 'opt+sp' means PBE structure optimized via stress tensor and then a single-point calculation to adjust the plane wave cutoff.

System	Final Conf.	Functional	Structure	Spin	E_{tot}	m_{tot}	$m_{loc}^{M'}$	$m_{loc}^{M''}$	E_g
Mo_2CO_2	hcp	PBE	opt+sp	without	−9.807	–	–	–	metal
		PBE	sp	with	−9.807	–	–	–	metal
		PBE+U	sp	with	−8.146	–	–	–	metal
Cr_2CO_2	fcc2	PBE	opt+sp	with	−9.380	1.63	1.53	–	metal
		PBE+U	sp	with	−7.985	0.73	2.14	–	metal
		PBE+U	opt+sp	with	−8.116	0.00	2.41	–	0.36
	fcc1	PBE	opt+sp	with	−9.402	9.54	1.37	–	metal
		PBE+U	sp	with	−8.013	15.31	2.22	–	metal

Continued on next page

		PBE+U	opt+sp	with	−8.104	15.23	2.27	–	metal
	hcp	PBE	opt+sp	without	−9.450	–	–	–	metal
		PBE	sp	with	−9.450	–	–	–	metal
		PBE+U	sp	with	−7.618	2.64	0.41	–	metal
		PBE+U	opt+sp	with	−7.769	0.86	2.13	–	metal
		PBE+U	opt+sp	with	−7.842	15.11	2.25	–	metal
$MnNbCO_2$	i-fcc-fcc	PBE	opt+sp	with	−9.328	8.10	2.19	0.11	metal
		PBE+U	sp	with	−8.121	15.09	3.84	0.08	metal
		PBE+U	opt+sp	with	−8.316	16.48	4.26	0.06	metal
		PBE+U	opt+sp	with	−8.271	16.32	4.22	0.06	metal
		PBE+U	opt+sp	with	−8.258	16.30	4.17	0.07	0.53
		PBE+U	opt+sp	with	−8.239	16.26	4.17	0.09	0.11
		PBE+U	opt+sp	with	−8.234	0.05	4.06	0.04	0.45
	i-fcc-hcp	PBE+U	opt+sp	with	−8.124	14.72	3.96	0.09	metal
		PBE+U	opt+sp	with	−8.100	14.67	3.94	0.09	metal
$MoVCO_2$	o-hcp-fcc	PBE	opt+sp	with	−9.844	3.54	0.06	0.85	metal
		PBE+U	sp	with	−8.358	4.00	0.04	1.03	metal
		PBE+U	opt+sp	with	−8.355	4.03	0.04	1.18	metal
		PBE+U	opt+sp	with	−8.355	4.03	0.04	1.18	metal
	i-hcp-fcc	PBE+U	opt+sp	with	−8.234	–	–	–	0.26
		PBE+U	opt+sp	with	−8.320	0.95	0.06	1.07	metal
		PBE+U	opt+sp	with	−8.164	1.81	0.40	0.81	metal
		PBE+U	opt+sp	with	−8.192	1.75	0.15	1.22	0.11
	i-hcp-hcp	PBE+U	opt+sp	with	−8.211	2.61	0.09	0.70	metal
		PBE+U	opt+sp	with	−8.212	3.32	0.08	0.78	metal
	o-hcp-hcp	PBE+U	opt+sp	with	−8.248	3.79	0.02	0.98	metal
		PBE+U	opt+sp	with	−8.233	0.00	0.01	0.97	metal
	i-fcc-fcc	PBE+U	opt+sp	with	−8.300	2.17	0.22	1.28	metal
		PBE+U	opt+sp	with	−8.299	2.78	0.14	1.25	metal
	o-fcc-fcc	PBE+U	opt+sp	with	−8.250	1.04	0.24	0.75	metal
		PBE+U	opt+sp	with	−8.259	2.03	0.34	1.03	metal
$CrMoNO_2$	o-fcc-hcp	PBE	opt+sp	with	−9.744	10.84	2.58	0.09	0.56
		PBE+U	sp	with	−8.444	11.05	2.71	0.05	0.85
		PBE+U	opt+sp	with	−8.445	0.00	2.64	0.03	0.80
		PBE+U	opt+sp	with	−8.450	11.04	2.72	0.05	0.71
	o-hcp-hcp	PBE+U	opt+sp	with	−8.450	0.00	2.62	0.03	0.87
	i-hcp-hcp	PBE+U	opt+sp	with	−8.329	7.73	2.61	0.63	0.44
		PBE+U	opt+sp	with	−8.331	7.73	2.62	0.64	0.47
$MoNbNO_2$	i-hcp-hcp	PBE	opt+sp	without	−10.070	–	–	–	metal
		PBE	sp	with	−10.070	–	–	–	metal
		PBE+U	sp	with	−8.646	–	–	–	metal
$NbYBO_2$	i-fcc-fcc	PBE	opt+sp	without	−9.320	–	–	–	0.08
		PBE	sp	with	−9.320	–	–	–	0.08
		PBE+U	sp	with	−8.552	–	–	–	0.17
$Ti_3C_2O_2$	fcc	PBE	opt+sp	without	−10.248	–	–	–	metal
		PBE	sp	with	−10.248	–	–	–	metal

Continued on next page

		PBE+U	sp	with	−9.169	–	–	–	metal
Ti_3CNO_2	i-fcc-fcc	PBE	opt+sp	without	−10.424	–	–	–	metal
		PBE	sp	with	−10.420	–	–	–	metal
		PBE+U	sp	with	−9.324	–	–	–	metal
Nb_3CNO_2	i-fcc-fcc	PBE	opt+sp	without	−10.530	–	–	–	metal
		PBE	sp	with	−10.549	–	–	–	metal
		PBE+U	sp	with	−9.214	–	–	–	metal
		PBE+U	opt+sp	with	−9.222	–	–	–	metal
Ti_2NbCNO_2	o-fcc-fcc	PBE	opt+sp	without	−10.505	–	–	–	metal
		PBE	sp	with	−10.424	–	–	–	metal
		PBE+U	sp	with	−9.331	–	–	–	metal
		PBE+U	opt+sp	with	−9.341	–	–	–	metal
$V_4C_3O_2$	fcc	PBE	opt+sp	without	−10.268	–	–	–	metal
		PBE	sp	with	−10.268	–	–	–	metal
		PBE+U	sp	with	−8.642	2.27	0.68	–	metal
		PBE+U	opt+sp	with	−8.691	3.40	0.78	–	metal
		PBE+U	opt+sp	with	−8.692	0.00	0.80	–	metal
	hcp	PBE+U	opt+sp	with	−8.579	0.00	0.75	–	metal
		PBE+U	opt+sp	with	−8.579	0.00	0.75	–	metal
$Nb_4C_3O_2$	fcc	PBE	opt+sp	without	−10.673	–	–	–	metal
		PBE	sp	with	−10.673	–	–	–	metal
		PBE+U	sp	with	−9.312	–	–	–	metal
$Cr_4C_3O_2$	hcp	PBE	opt+sp	with	−9.806	0.00	0.28	–	metal
		PBE+U	sp	with	−7.964	0.00	1.14	–	metal
		PBE+U	opt+sp	with	−8.129	0.00	2.07	–	metal
		PBE+U	opt+sp	with	−8.144	0.00	2.39	–	metal
	fcc	PBE+U	opt+sp	with	−8.292	32.46	2.35	–	metal
		PBE+U	opt+sp	with	−8.263	0.14	2.24	–	metal

Table S-6: Summary of properties for PBE and PBE+U structure space exploration. For PBE+U calculations, we considered different structures within a range of 100 meV from PBE calculations, including AFM and FM configurations; see Table S-3. E_{tot} is given in (eV/atom). a_0, b_0, and d_z are given in (Å). 'sp' and 'opt' stand for single-point and optimization calculations. 'with' and 'without' stand for spin-polarized and unpolarized calculations. 'opt+sp' means PBE structure optimized via stress tensor and then a single-point calculation to adjust the plane wave cutoff. We also indicate the final magnetic configuration.

System	Final Conf.	Functional	Structure	Spin	E_{tot}	a_0	b_0	d_z
Cr_2CO_2	fcc2	PBE	opt+sp	with, FIM	−9.380	2.859	2.884	4.40
		PBE+U	opt+sp	with, AFM	−8.116	3.053	2.969	4.93
	fcc1	PBE	opt+sp	with, FIM	−9.402	2.856	2.841	4.50
		PBE+U	opt+sp	with, FIM	−8.104	2.920	2.922	4.62
	hcp	PBE	opt+sp	without, NM	−9.451	2.668	2.668	4.85
		PBE+U	opt+sp	with, FIM	−7.769	2.883	2.876	4.72

Continued on next page

		PBE+U	opt+sp	with, FIM	−7.842	2.851	2.850	4.82
$MnNbCO_2$	i-fcc-fcc	PBE	opt+sp	with, FIM	−9.328	3.023	2.987	4.55
		PBE+U	opt+sp	with, FIM	−8.316	3.141	3.136	4.61
		PBE+U	opt+sp	with, FIM	−8.271	3.078	3.197	4.67
		PBE+U	opt+sp	with, FIM	−8.239	3.085	3.172	4.61
		PBE+U	opt+sp	with, FIM	−8.234	3.034	3.201	4.64
	i-fcc-hcp	PBE+U	opt+sp	with, FIM	−8.124	3.044	3.055	4.90
		PBE+U	opt+sp	with, FIM	−8.100	3.038	3.037	4.89
$MoVCO_2$	o-hcp-fcc	PBE	opt+sp	with, FIM	−9.844	2.892	2.892	4.80
		PBE+U	opt+sp	with, FIM	−8.355	2.925	2.919	4.81
		PBE+U	opt+sp	with, FIM	−8.355	2.925	2.919	4.81
	i-hcp-fcc	PBE+U	opt+sp	with, NM	−8.234	2.925	2.895	4.92
		PBE+U	opt+sp	with, FIM	−8.320	2.939	2.943	4.82
		PBE+U	opt+sp	with, FIM	−8.164	2.945	2.944	4.94
		PBE+U	opt+sp	with, FIM	−8.192	2.949	2.954	4.92
	i-hcp-hcp	PBE+U	opt+sp	with, FIM	−8.211	2.867	2.866	5.03
		PBE+U	opt+sp	with, FIM	−8.212	2.868	2.866	5.03
	o-hcp-hcp	PBE+U	opt+sp	with, FIM	−8.248	2.877	2.870	4.96
		PBE+U	opt+sp	with, AFM	−8.233	2.881	2.894	4.95
	i-fcc-fcc	PBE+U	opt+sp	with, FIM	−8.300	3.055	3.013	4.73
		PBE+U	opt+sp	with, FIM	−8.299	3.044	3.000	4.74
	o-fcc-fcc	PBE+U	opt+sp	with, FIM	−8.250	2.983	2.979	4.79
		PBE+U	opt+sp	with, FIM	−8.259	2.982	2.991	4.74
$CrMoNO_2$	o-fcc-hcp	PBE	opt+sp	with, FM	−9.744	2.872	2.872	4.85
		PBE+U	opt+sp	with, AFM	−8.445	2.896	2.902	4.86
		PBE+U	opt+sp	with, FIM	−8.450	2.900	2.900	4.86
	o-hcp-hcp	PBE+U	opt+sp	with, FIM	−8.450	2.892	2.898	4.91
	i-hcp-hcp	PBE+U	opt+sp	with, FIM	−8.329	2.915	2.918	4.94
		PBE+U	opt+sp	with, FIM	−8.331	2.906	2.925	4.92
$V_4C_3O_2$	fcc	PBE	opt+sp	with, NM	−10.268	2.856	2.841	9.23
		PBE+U	opt+sp	with, FIM	−8.691	2.959	2.953	9.36
		PBE+U	opt+sp	with, AFM	−8.692	2.969	2.941	9.35
	hcp	PBE+U	opt+sp	with, AFM	−8.579	2.900	2.899	9.59
		PBE+U	opt+sp	with, AFM	−8.579	2.899	2.899	9.59
$Cr_4C_3O_2$	hcp	PBE	opt+sp	with, AFM	−9.806	2.767	2.767	9.50
		PBE+U	opt+sp	with, AFM	−8.129	2.874	2.907	9.65
		PBE+U	opt+sp	with, FIM	−8.144	2.976	2.967	9.33
	fcc	PBE+U	opt+sp	with, FIM	−8.292	2.945	2.945	9.38
		PBE+U	opt+sp	with, FIM	−8.263	2.972	2.943	9.37

S-5.2 Density of States

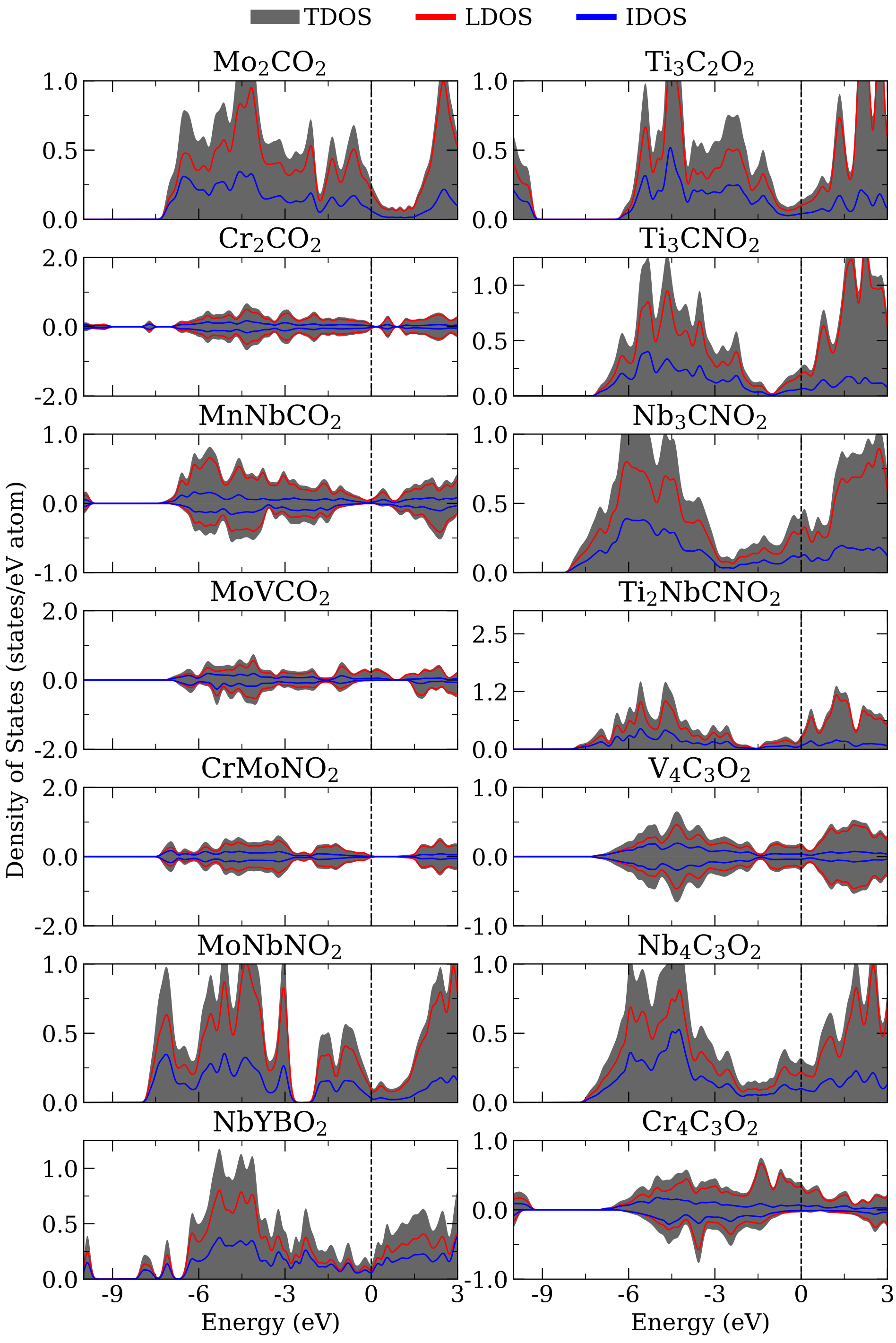

Figure S-4: Average total (TDOS), local (LDOS) and interstitial (IDOS) density of states for the lowest energy structure for all compositions. We used a Gaussian broadening of 0.1 eV. The vertical black dashed lines indicate the Fermi level.

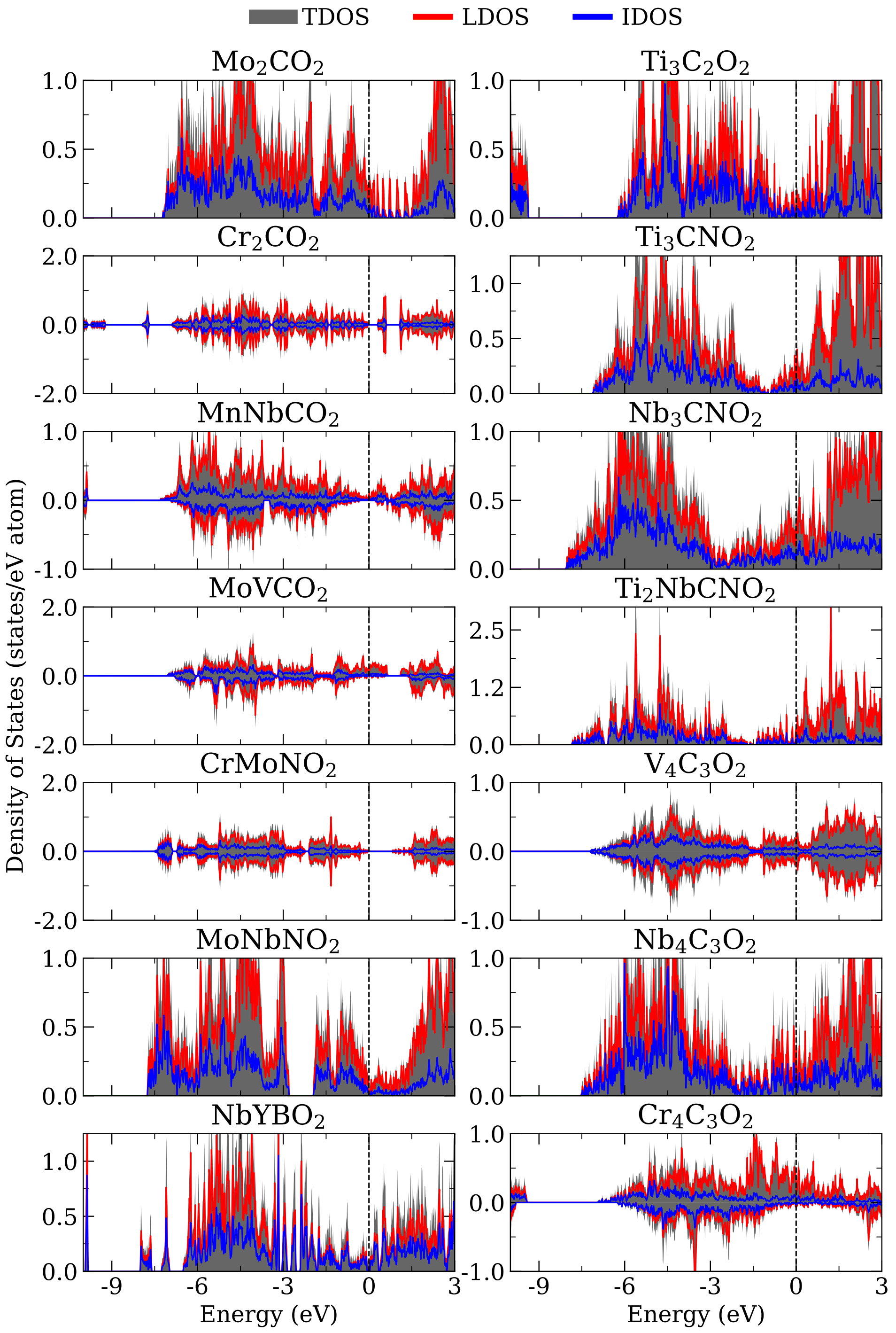

Figure S-5: Average total (TDOS), local (LDOS) and interstitial (IDOS) density of states for the lowest energy structure for all compositions. We used a Gaussian broadening of 0.01 eV. The vertical black dashed lines indicate the Fermi level.

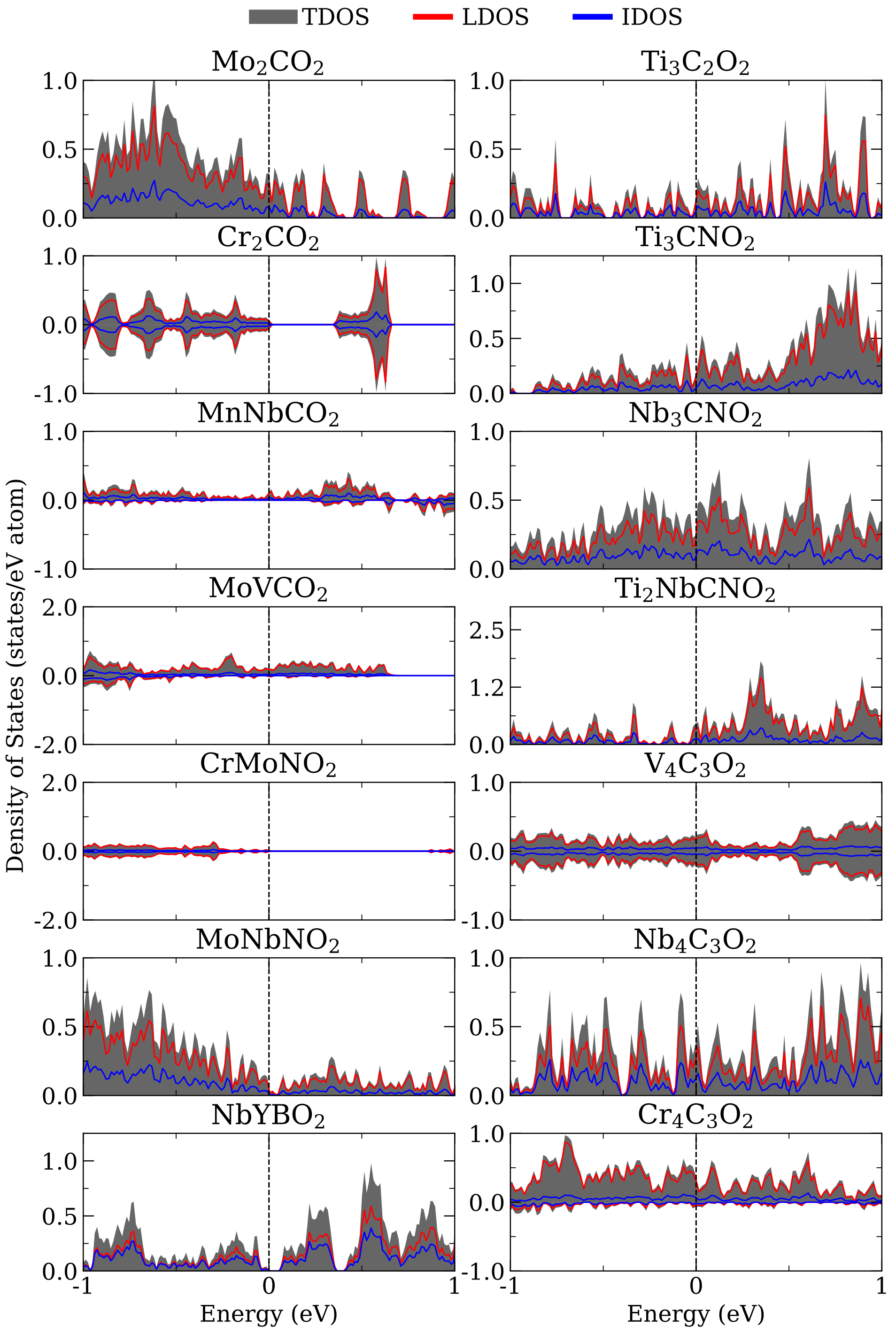

Figure S-6: Average total (TDOS), local (LDOS) and interstitial (IDOS) density of states for the lowest energy structure for all compositions. We used a Gaussian broadening of 0.01 eV. The vertical black dashed lines indicate the Fermi level.

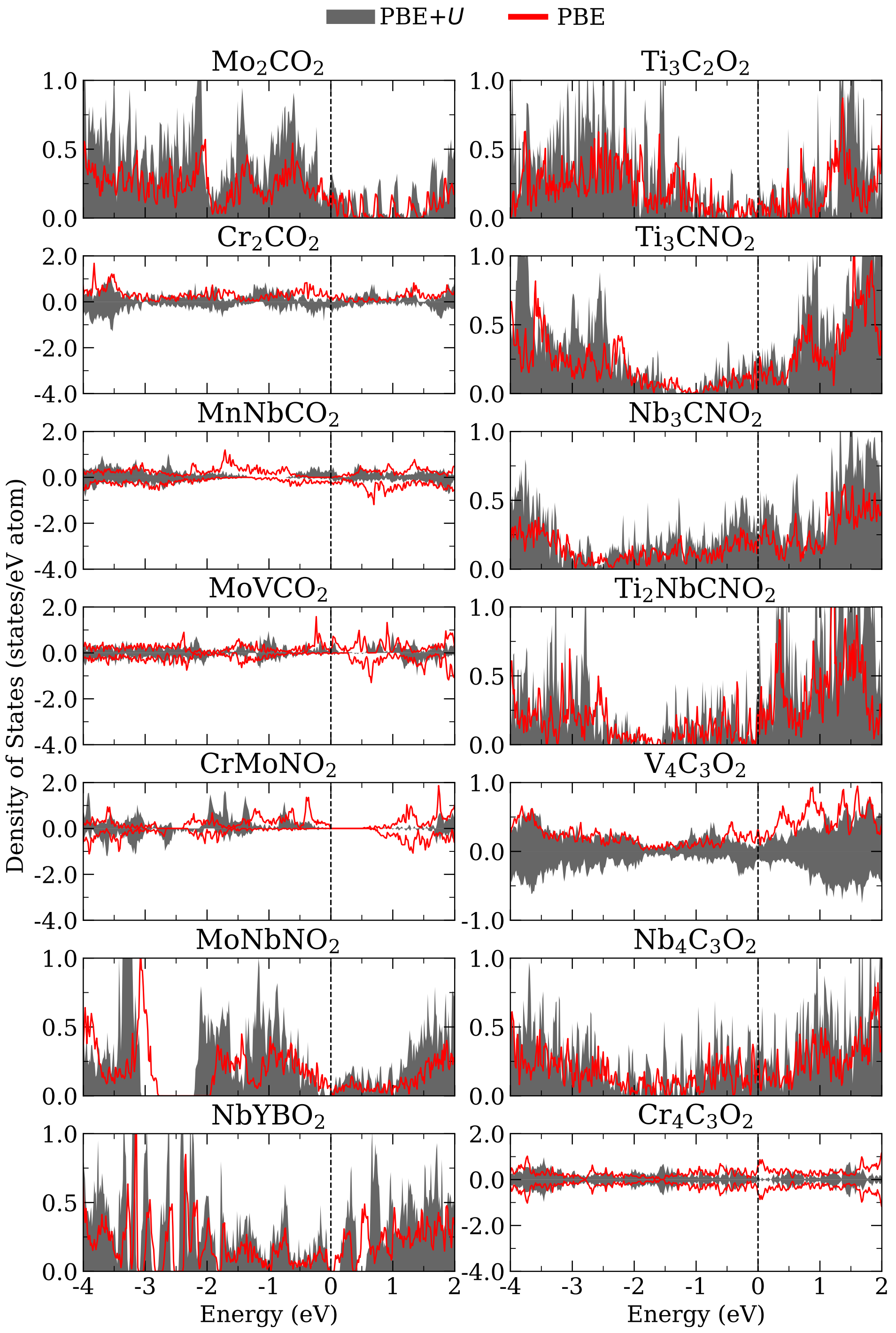

Figure S-7: Average total density of states (TDOS) for the lowest energy structure (PBE) and single-point PBE+U calculation for all compositions. We used a Gaussian broadening of 0.01 eV. The vertical black dashed lines indicate the Fermi level of PBE calculations.

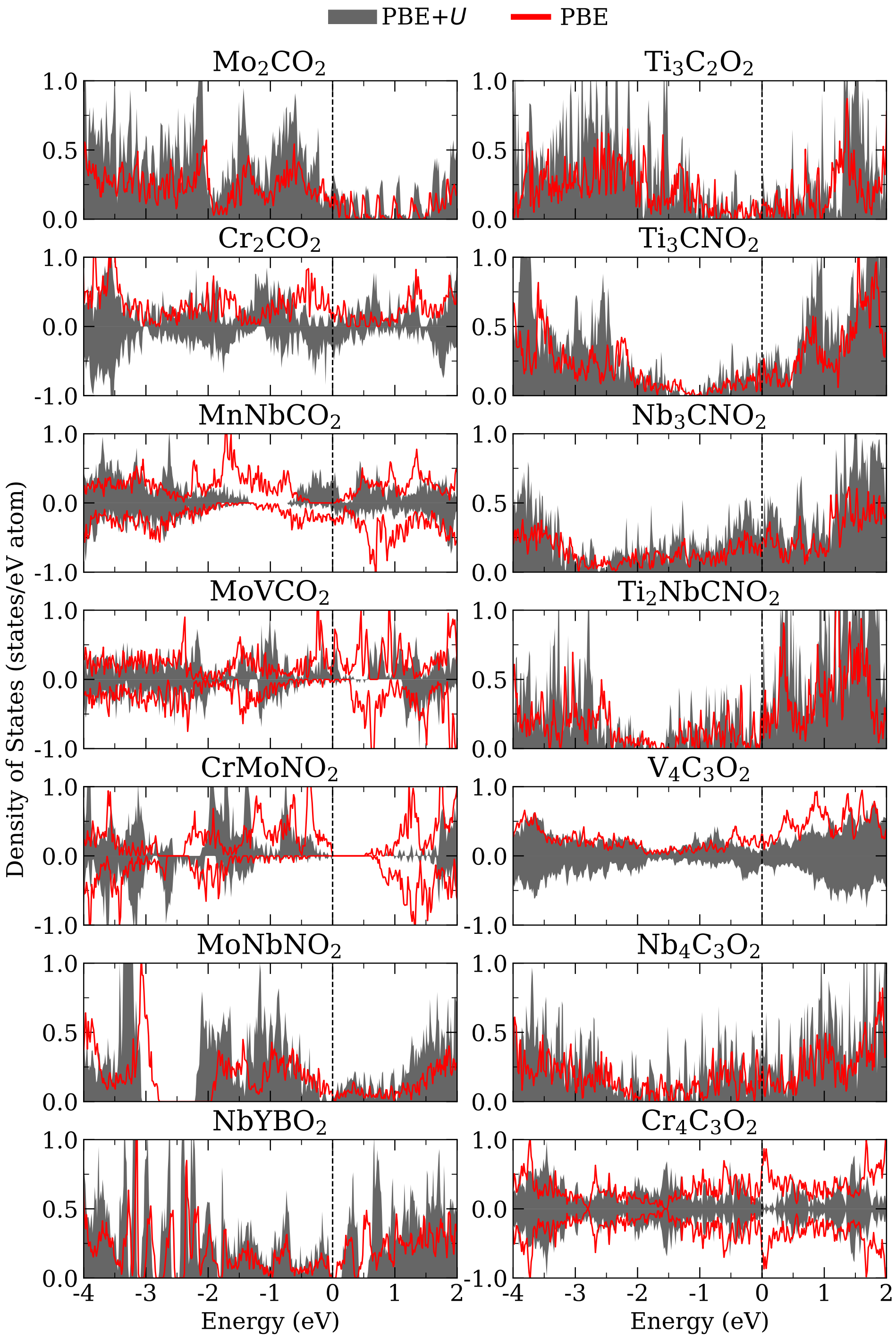

Figure S-8: Average total density of states (TDOS) for the lowest energy structure (PBE) and single-point PBE+U calculation for all compositions. We used a Gaussian broadening of 0.01 eV. The vertical black dashed lines indicate the Fermi level of PBE calculations.

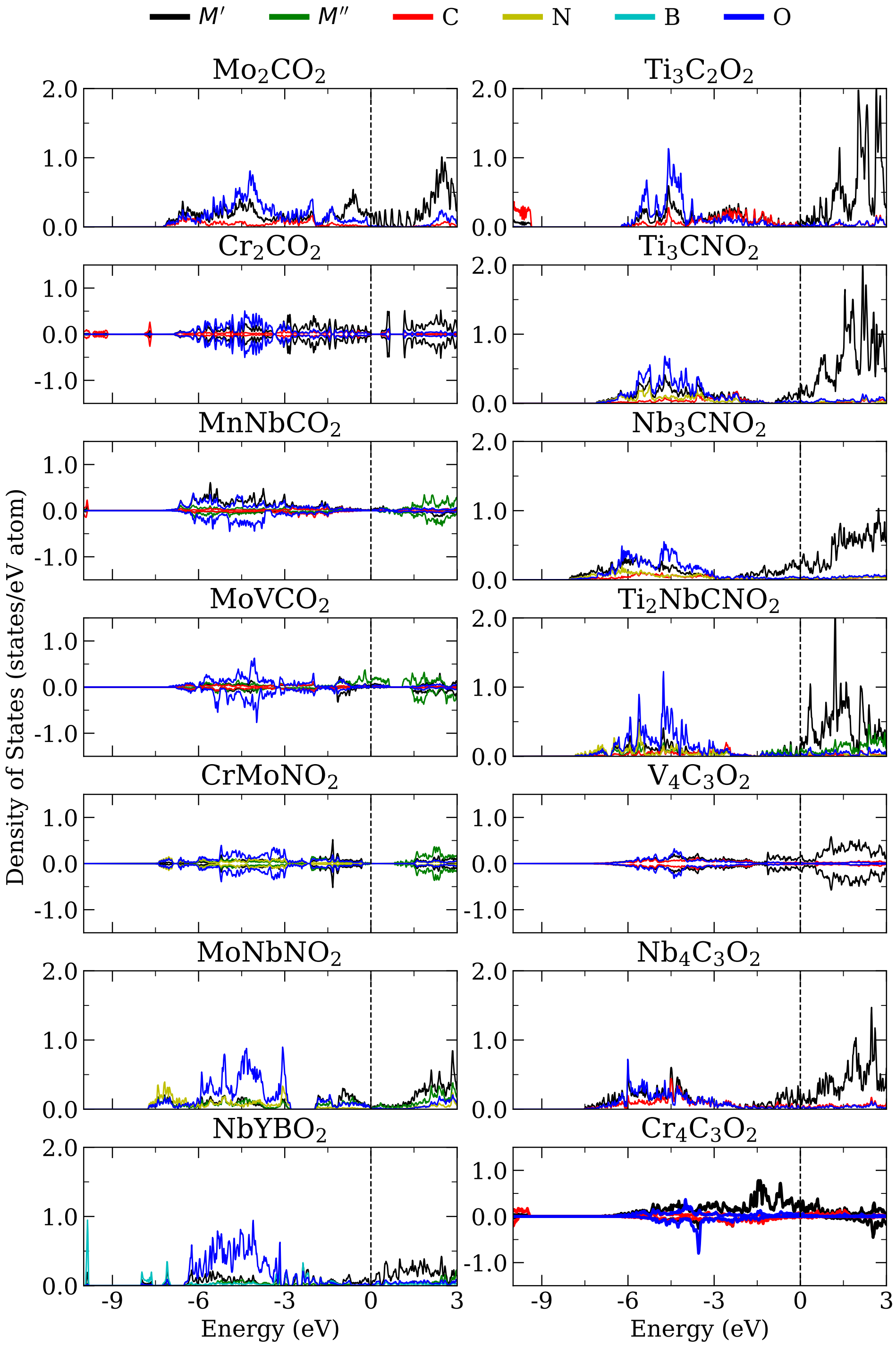

Figure S-9: Average local density of states (LDOS) for each chemical element. We considered the lowest energy structure for all compositions and the general formula $(M'M'')_{n+1}(X'X'')_nO_2$. We used a Gaussian broadening of 0.01 eV. The vertical black dashed lines indicate the Fermi level.

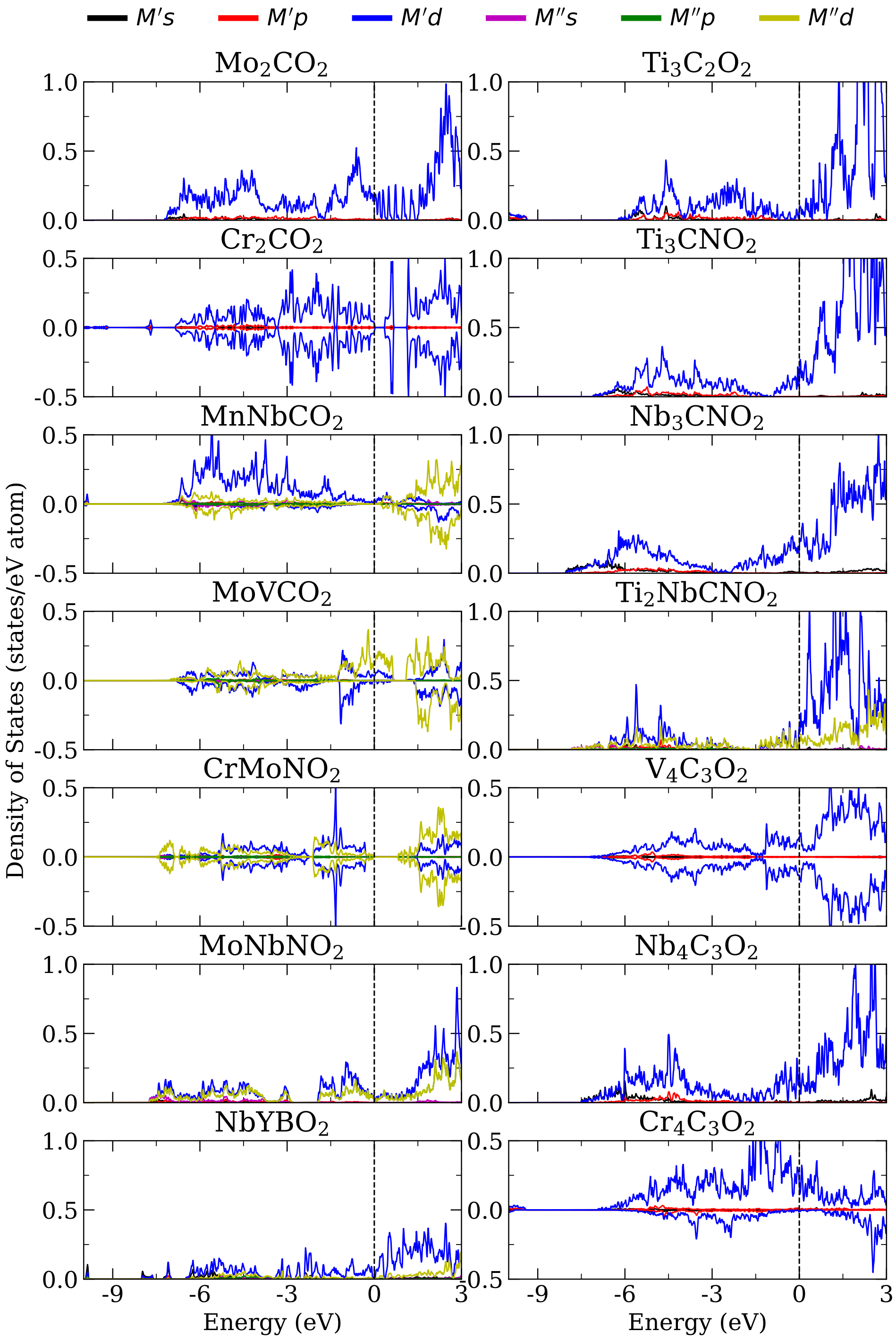

Figure S-10: Average density of states (DOS) for s, p, d states for metals. We considered the lowest energy structure for all compositions and the general formula $(M'M'')_{n+1}(X'X'')_nO_2$. We used a Gaussian broadening of 0.01 eV. The vertical black dashed lines indicate the Fermi level.

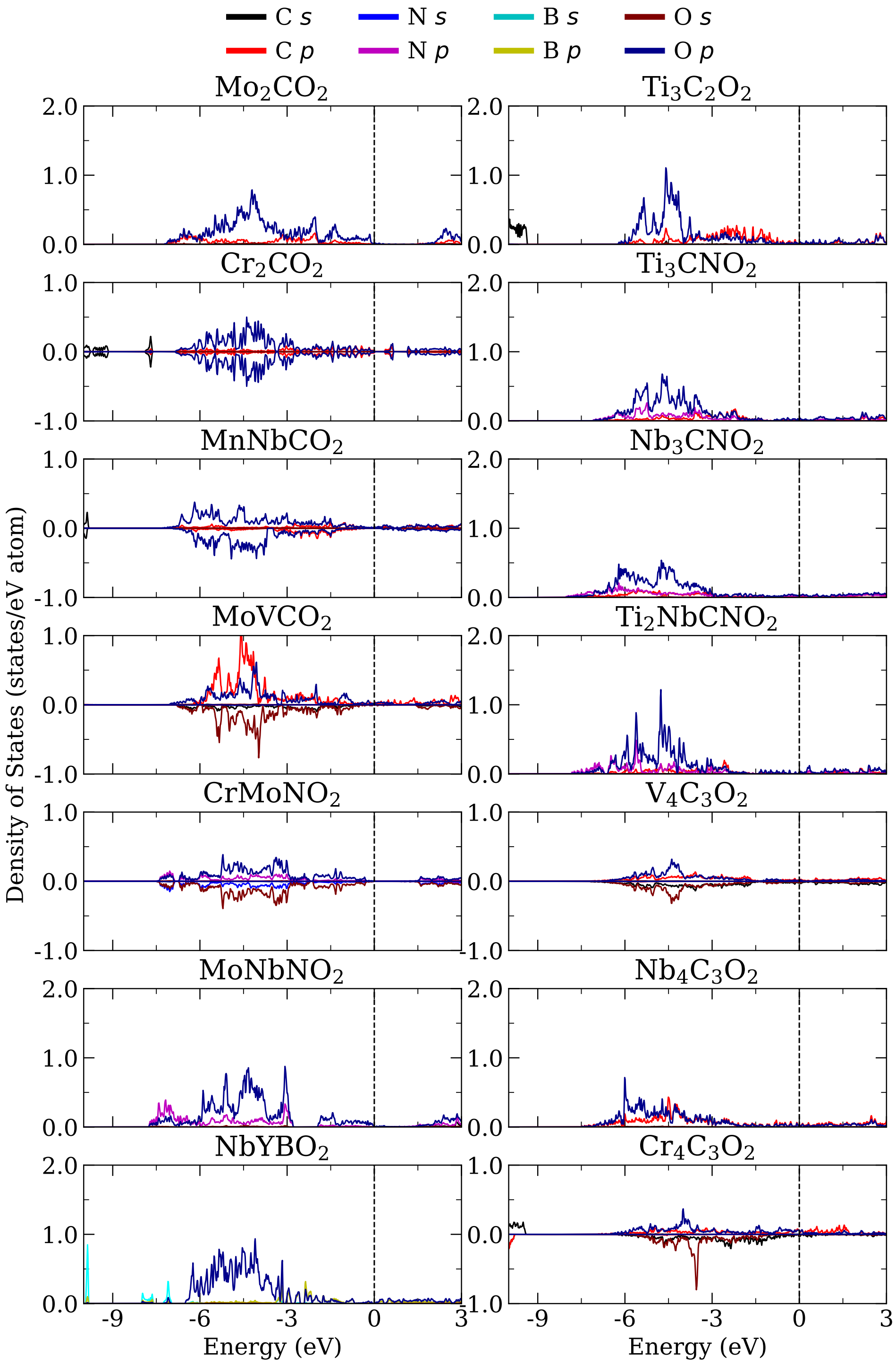

Figure S-11: Average density of states (DOS) for s, p, d states for metals. We considered the lowest energy structure for all compositions and the general formula $(M'M'')_{n+1}(X'X'')_nO_2$. We used a Gaussian broadening of 0.01 eV. The vertical black dashed lines indicate the Fermi level.

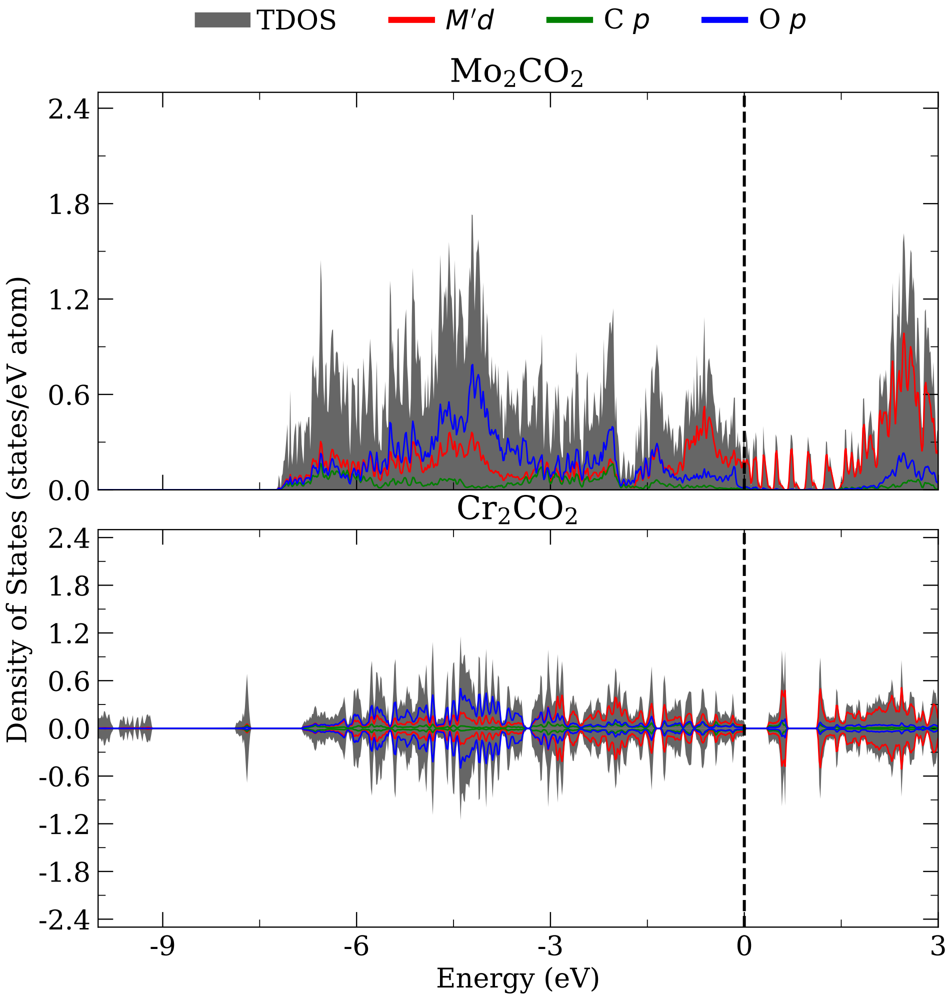

Figure S-12: Average total density of states (TDOS) and s, p, d states contribution for Mo_2CO_2 and Cr_2CO_2. We considered the lowest energy structure for all compositions and the general formula $(M'M'')_{n+1}(X'X'')_nO_2$. We used a Gaussian broadening of 0.01 eV. The vertical black dashed lines indicate the Fermi level.

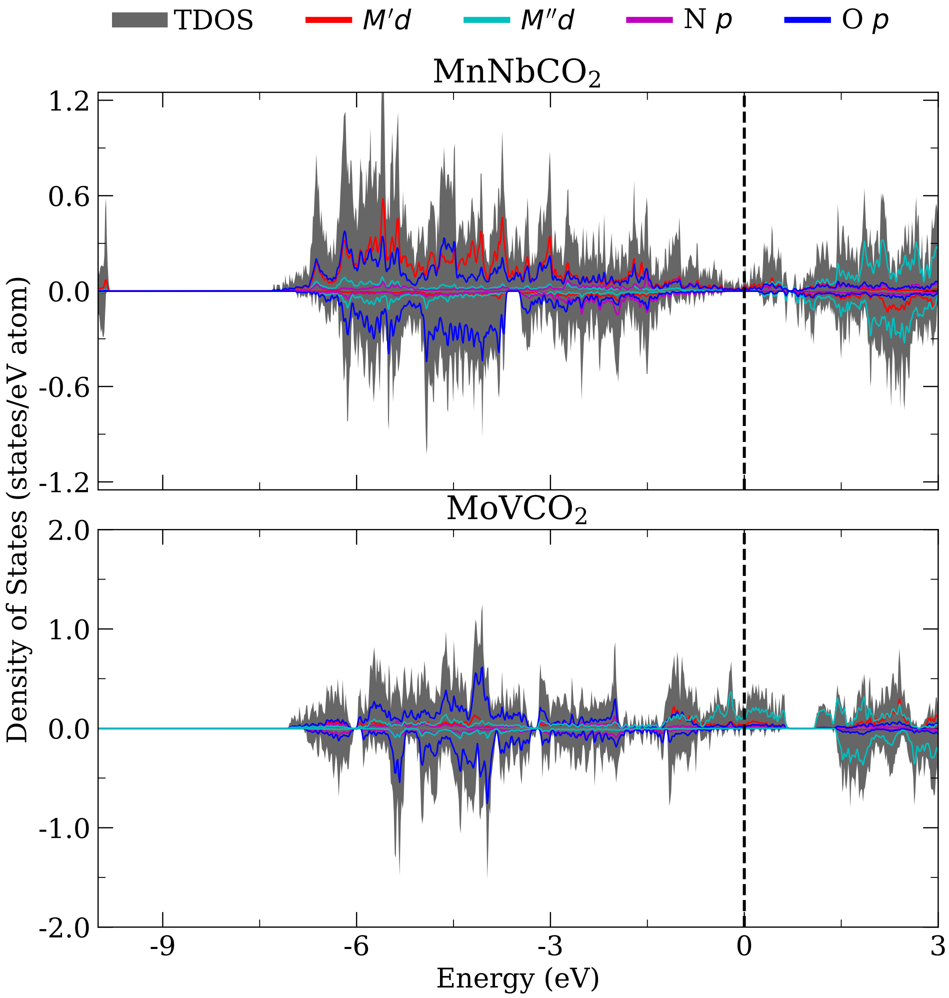

Figure S-13: Average total density of states (TDOS) and s, p, d states contribution for $MnNbCO_2$ and $MoVCO_2$. We considered the lowest energy structure for all compositions and the general formula $(M'M'')_{n+1}(X'X'')_nO_2$. We used a Gaussian broadening of 0.01 eV. The vertical black dashed lines indicate the Fermi level.

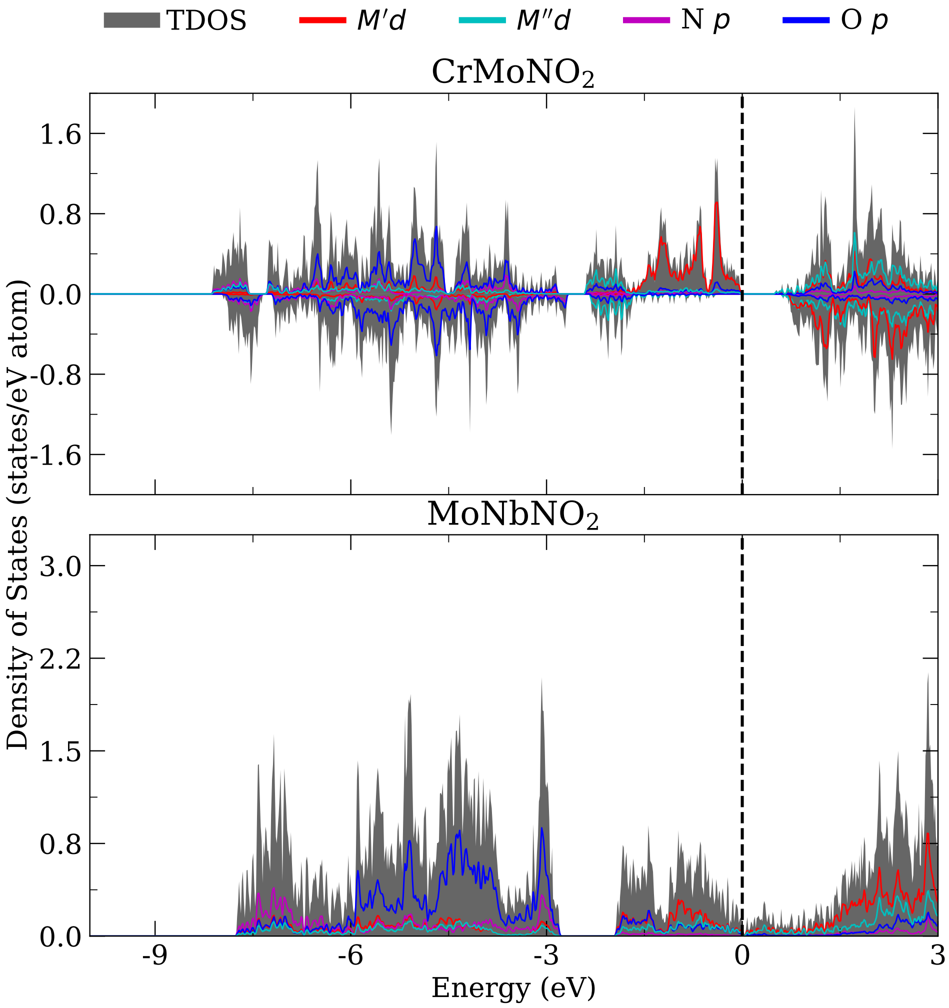

Figure S-14: Average total density of states (TDOS) and s, p, d states contribution for $CrMoNO_2$ and $MoNbNO_2$. We considered the lowest energy structure for all compositions and the general formula $(M'M'')_{n+1}(X'X'')_nO_2$. We used a Gaussian broadening of 0.01 eV. The vertical black dashed lines indicate the Fermi level.

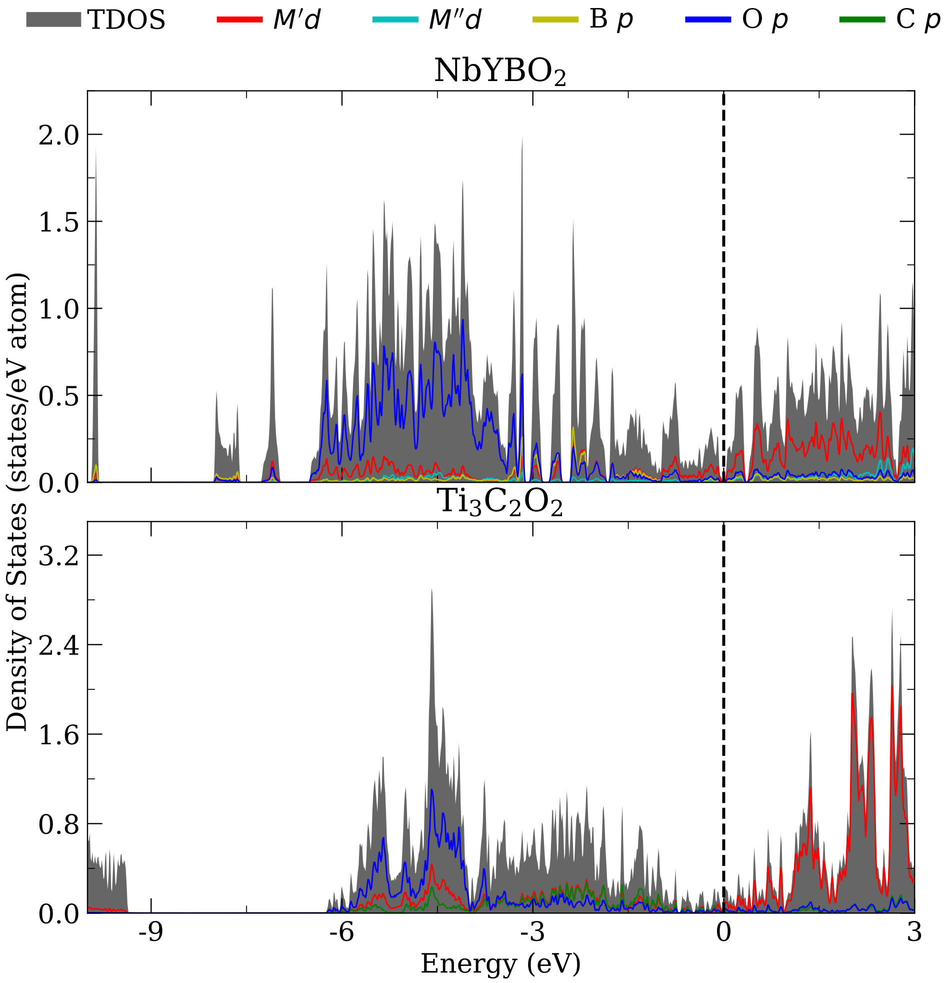

Figure S-15: Average total density of states (TDOS) and s, p, d states contribution for $NbYBO_2$ and $Ti_3C_2O_2$. We considered the lowest energy structure for all compositions and the general formula $(M'M'')_{n+1}(X'X'')_nO_2$. We used a Gaussian broadening of 0.01 eV. The vertical black dashed lines indicate the Fermi level.

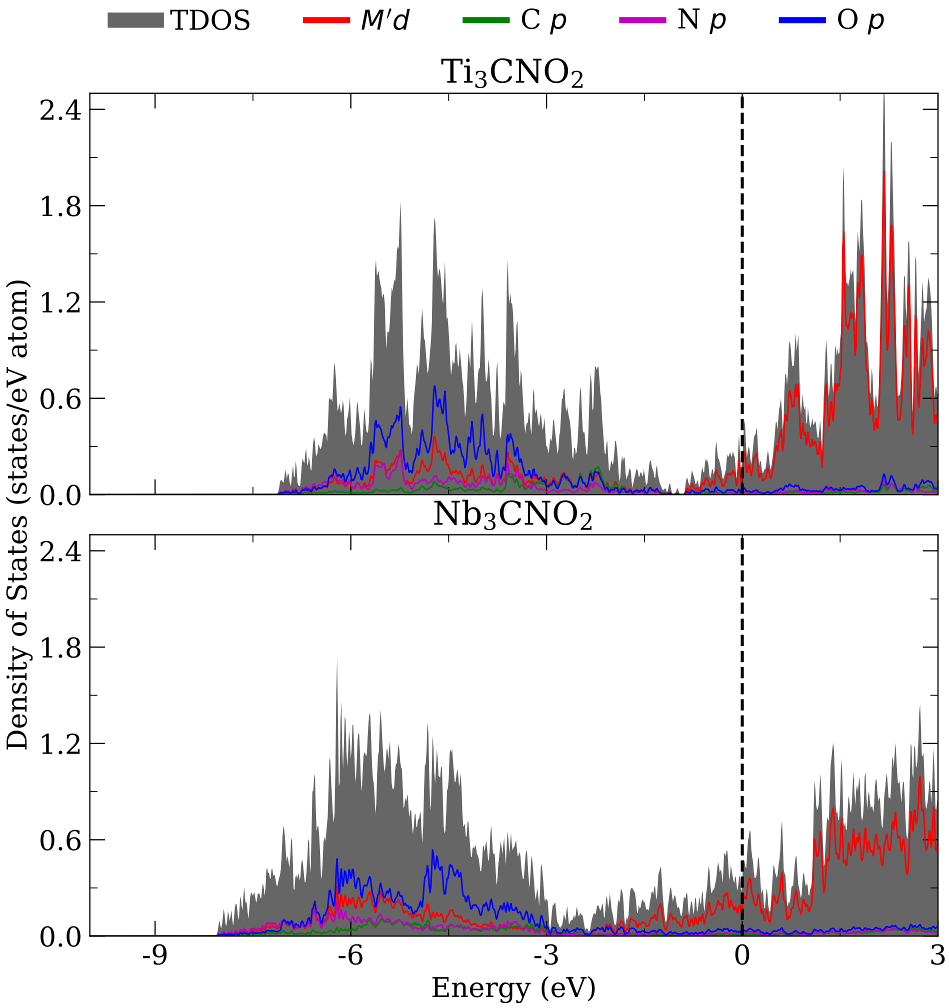

Figure S-16: Average total density of states (TDOS) and s, p, d states contribution for Ti_3CNO_2 and Nb_3CNO_2. We considered the lowest energy structure for all compositions and the general formula $(M'M'')_{n+1}(X'X'')_nO_2$. We used a Gaussian broadening of 0.01 eV. The vertical black dashed lines indicate the Fermi level.

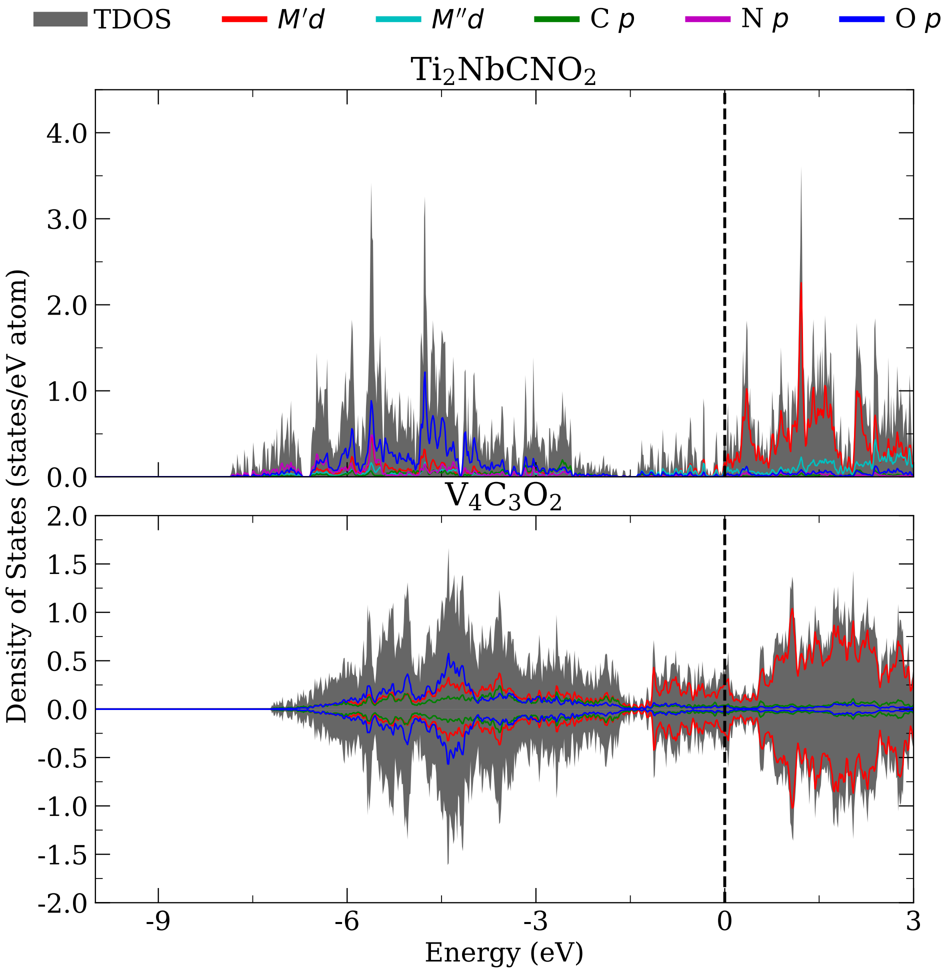

Figure S-17: Average total density of states (TDOS) and s, p, d states contribution for Ti_2NbCNO_2 and $V_4C_3O_2$. We considered the lowest energy structure for all compositions and the general formula $(M'M'')_{n+1}(X'X'')_nO_2$. We used a Gaussian broadening of 0.01 eV. The vertical black dashed lines indicate the Fermi level.

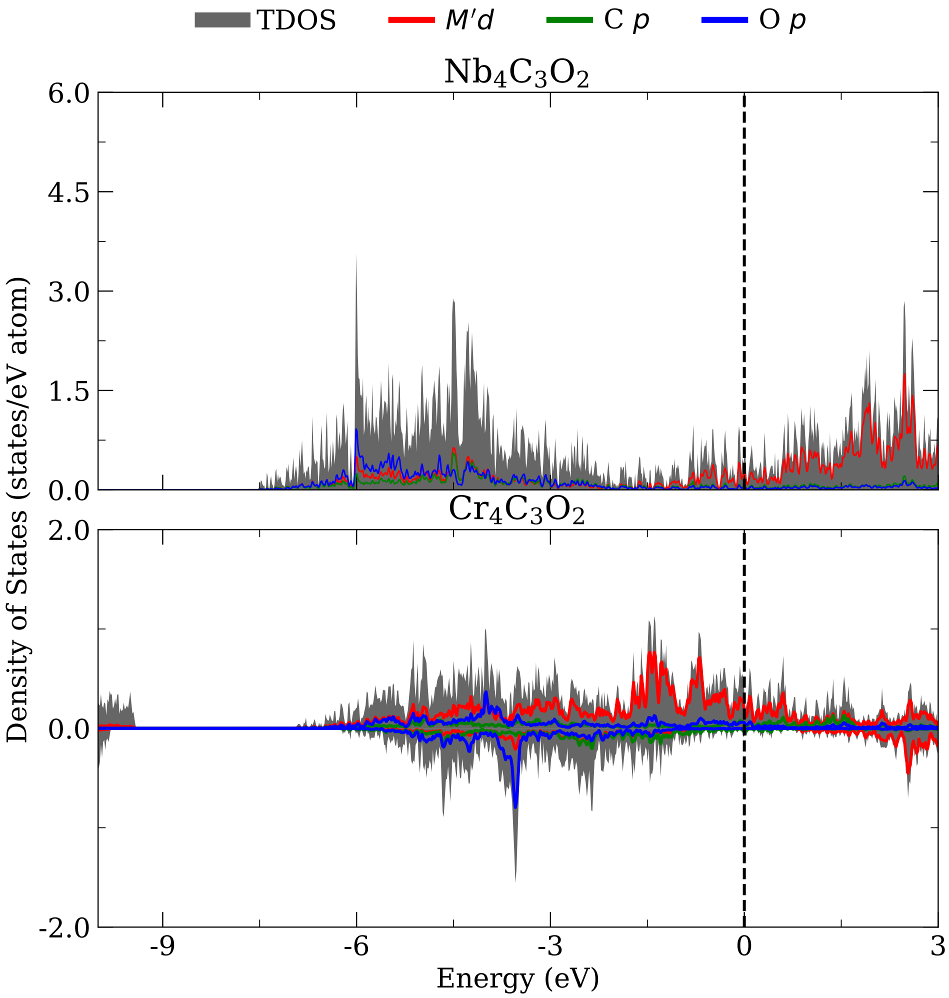

Figure S-18: Average total density of states (TDOS) and s, p, d states contribution for $Nb_4C_3O_2$ and $Cr_4C_3O_2$. We considered the lowest energy structure for all compositions and the general formula $(M'M'')_{n+1}(X'X'')_nO_2$. We used a Gaussian broadening of 0.01 eV. The vertical black dashed lines indicate the Fermi level.

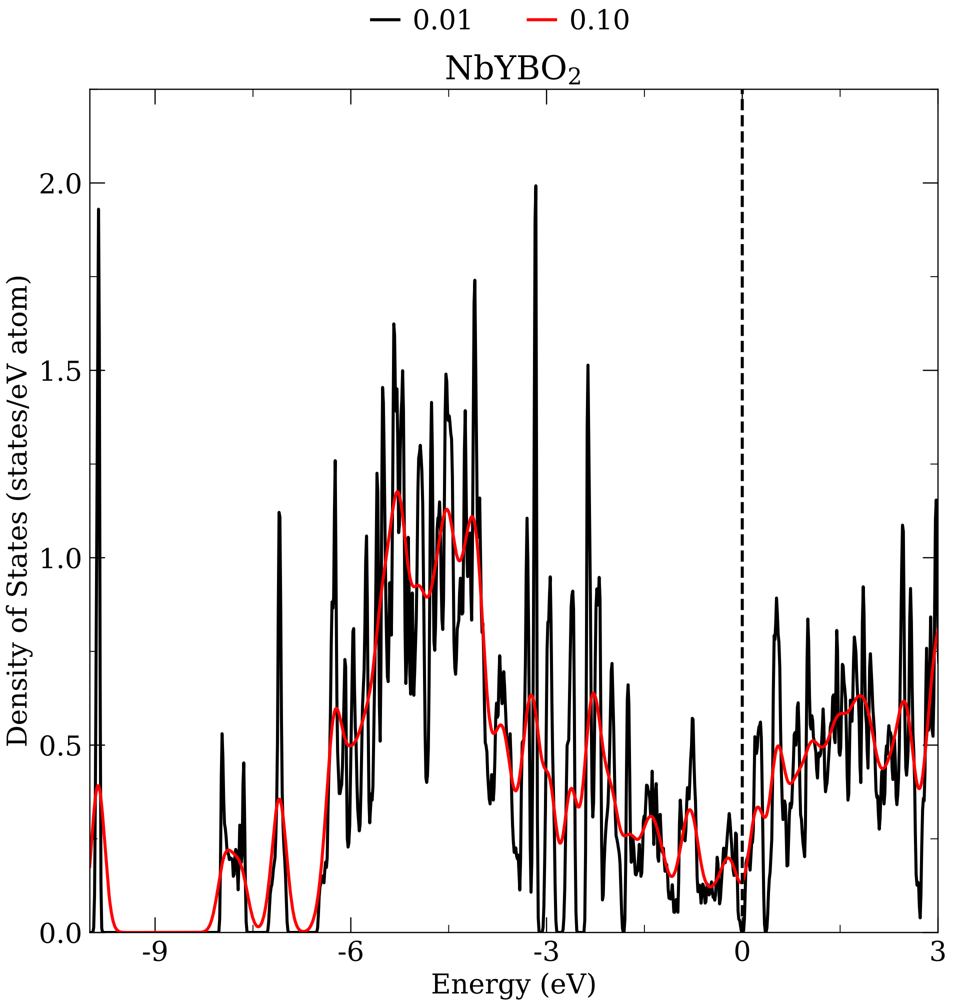

Figure S-19: Average total density of states (TDOS) for $NbYBO_2$ for different Gaussian broadenings, eV. We considered the lowest energy structure for all compositions. The vertical black dashed lines indicate the Fermi level.

Figure S-4–S-18 shows the metallic character of most MXenes for PBE and PBE+U calculations. The d-states of the metal are the main contribution the density of states near the Fermi level. Figure S-19 shows that one must be careful with the Gaussian broadening value used to plot the DOS, once a very large value can turn a system with a small bandgap into a metallic system. Table S-7 shows the results of d-states occupancies. The calculated orbital occupancies for d_{xy}, d_{yz}, d_{xz}, and $d_{x^2-y^2}$ represent a combination of orbital characters due to hexagonal symmetry. Nevertheless, there is a relation between fcc/hcp structures and d_{z^2} occupancy, i.e., for hcp terminations, the d_{z^2} occupancy is always larger than other projected d-states. It is worth noting that this analysis is qualitative because interstitial regions are not considered in the integration of projected DOS.

Table S-7: Qualitative occupancies analysis of d states (d^{occ}) calculated via integration of density of states until the Fermi level for metallic systems or valence band maximum for semiconductor systems. We considered the general formula $(M'M'')_{n+1}(X'X'')_nO_2$. For spin-polarized systems, we summed the up and down contributions for each projected state. The values are normalized per atom.

System	$M'd^{occ}_{xy}$	$M'd^{occ}_{yz}$	$M'd^{occ}_{z^2}$	$M'd^{occ}_{xz}$	$M'd^{occ}_{x^2-y^2}$	$M''d^{occ}_{xy}$	$M''d^{occ}_{yz}$	$M''d^{occ}_{z^2}$	$M''d^{occ}_{xz}$	$M''d^{occ}_{x^2-y^2}$
Mo_2CO_2	0.62	0.52	0.68	0.51	0.63	–	–	–	–	–
Cr_2CO_2	0.72	0.70	0.74	0.67	0.68	–	–	–	–	–
$MnNbCO_2$	1.03	1.03	1.01	1.03	1.02	0.30	0.37	0.25	0.37	0.33
$MoVCO_2$	0.62	0.51	0.60	0.51	0.62	0.54	0.55	0.23	0.55	0.53
$CrMoNO_2$	0.71	0.60	0.94	0.60	0.70	0.60	0.45	0.72	0.45	0.60
$MoNbNO_2$	0.67	0.43	0.77	0.43	0.66	0.42	0.32	0.45	0.32	0.43
$NbYBO_2$	0.39	0.41	0.31	0.44	0.36	0.11	0.09	0.08	0.09	0.11
$Ti_3C_2O_2$	0.31	0.39	0.22	0.35	0.29	–	–	–	–	–
Ti_3CNO_2	0.30	0.35	0.25	0.35	0.31	–	–	–	–	–
Nb_3CNO_2	0.39	0.38	0.38	0.39	0.38	–	–	–	–	–
Ti_2NbCNO_2	0.08	0.09	0.06	0.08	0.07	0.11	0.10	0.10	0.10	0.08
$V_4C_3O_2$	0.42	0.58	0.33	0.51	0.57	–	–	–	–	–
$Nb_4C_3O_2$	0.42	0.40	0.35	0.41	0.33	–	–	–	–	–
$Cr_4C_3O_2$	0.71	0.71	0.73	0.70	0.71	–	–	–	–	–

S-5.3 Band Structure

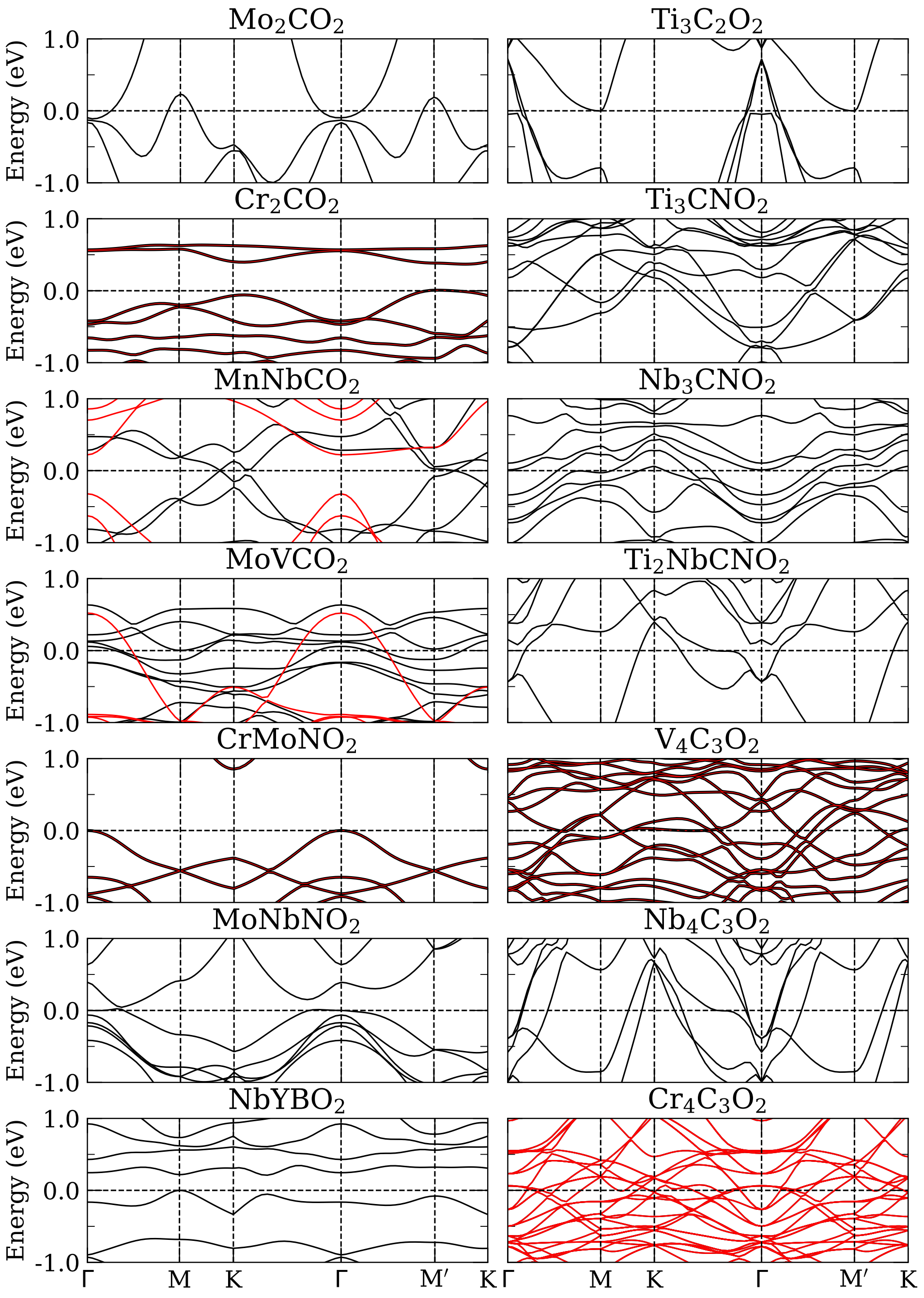

Figure S-20: Band structure for the lowest energy structure for all compositions. The horizontal black dashed lines indicate the valence band maximum (VBM) for semiconductors or the Fermi level for metallic systems. For spin-polarized systems, the black and red lines indicate the spin up and down.

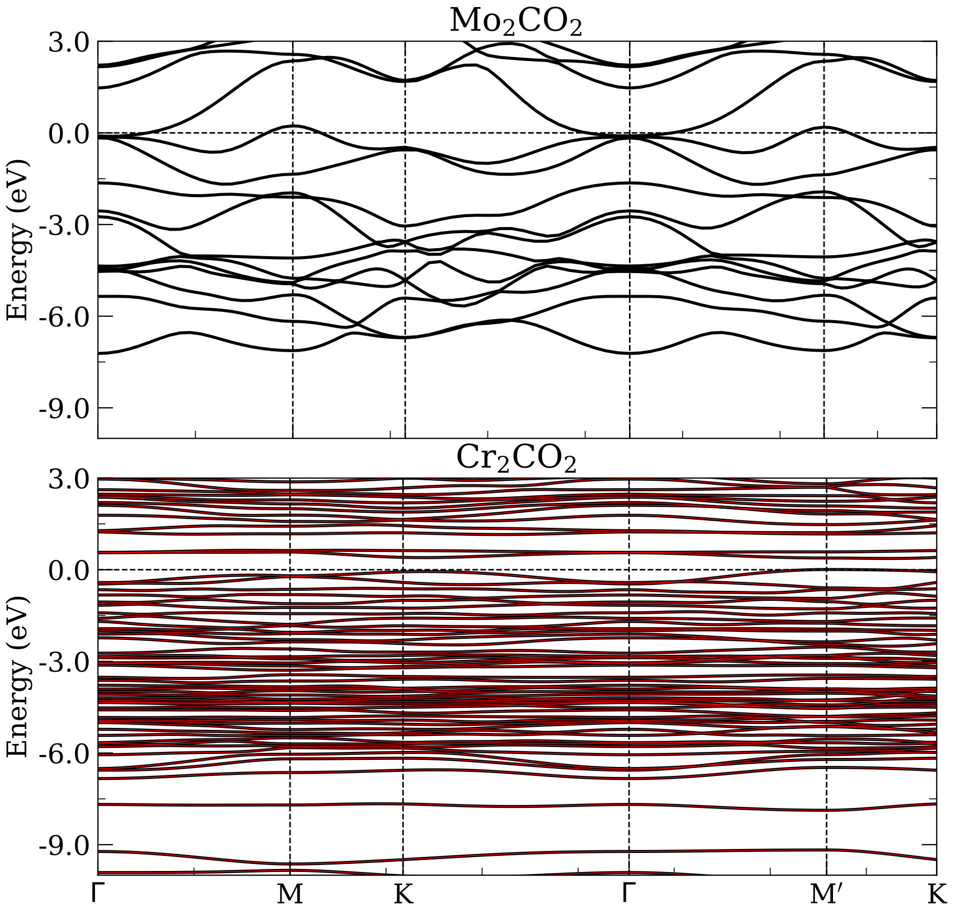

Figure S-21: Band structure for Mo_2CO_2 and Cr_2CO_2. We considered the lowest energy structure for all compositions. The horizontal black dashed lines indicate the valence band maximum (VBM) for semiconductors or the Fermi level for metallic systems.

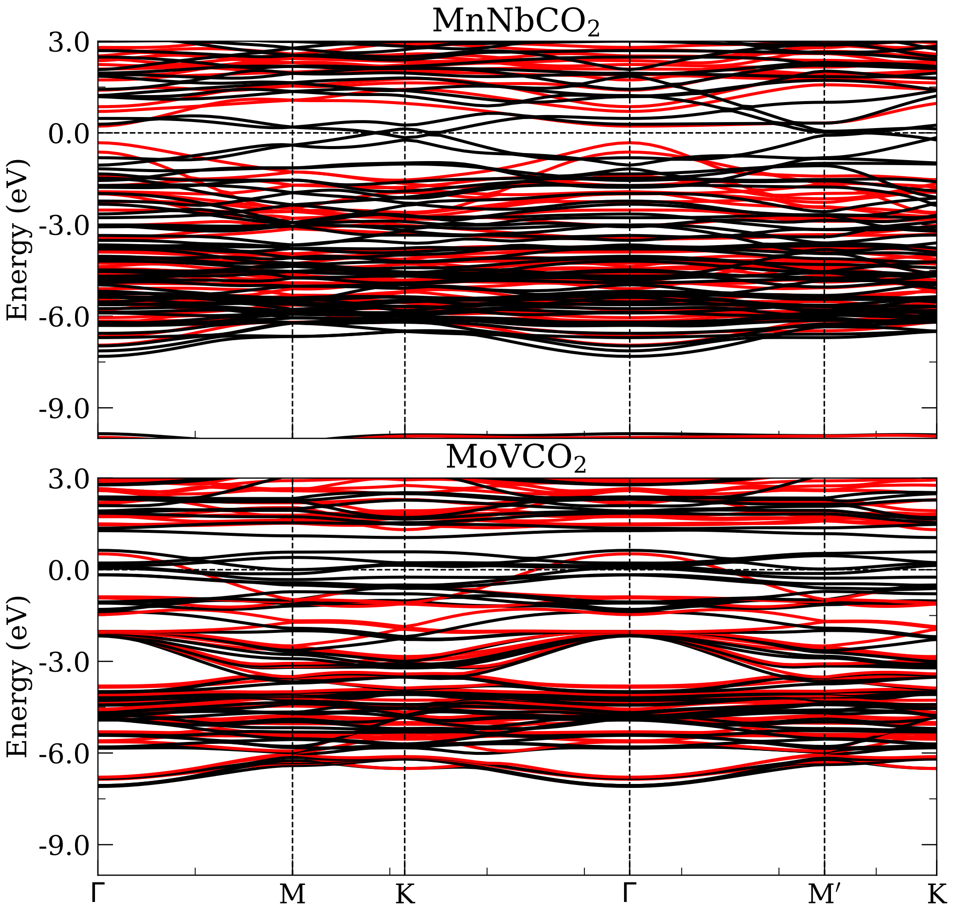

Figure S-22: Band structure for $MnNbCO_2$ and $MoVCO_2$. We considered the lowest energy structure for all compositions. The horizontal black dashed lines indicate the valence band maximum (VBM) for semiconductors or the Fermi level for metallic systems. For spin-polarized systems, the black and red lines indicate the spin up and down.

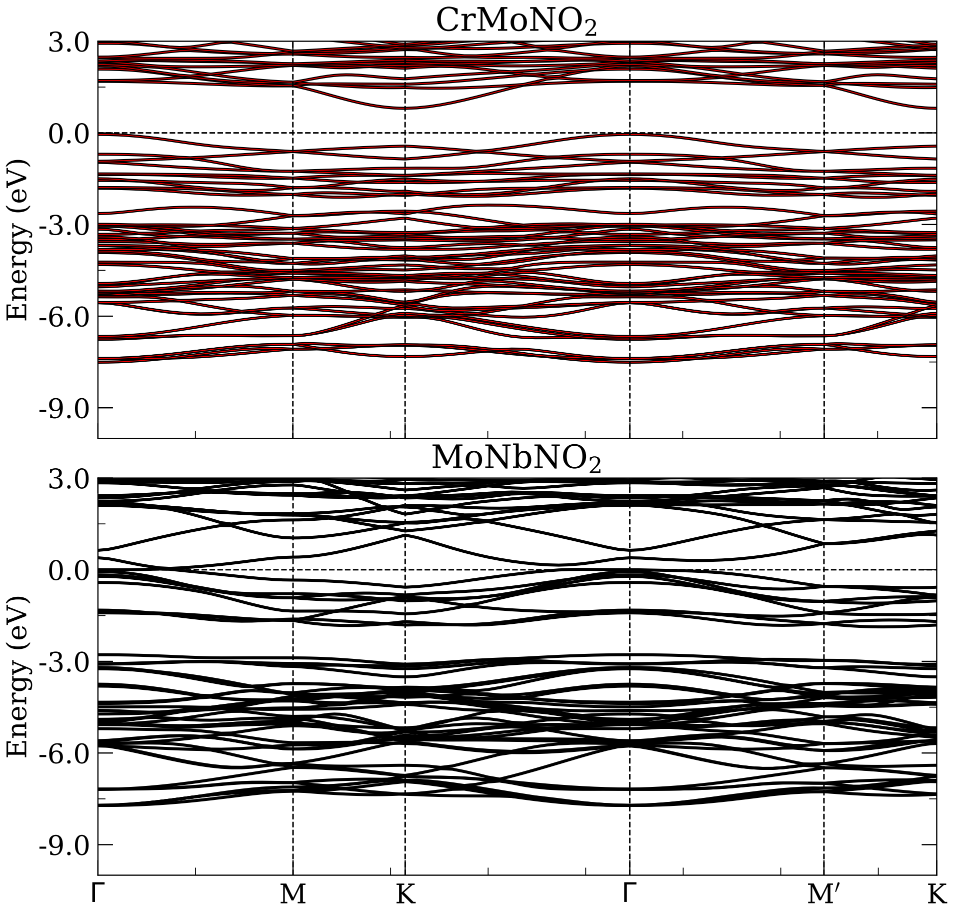

Figure S-23: Band structure for $CrMoNO_2$ and $MoNbNO_2$. We considered the lowest energy structure for all compositions. The horizontal black dashed lines indicate the valence band maximum (VBM) for semiconductors or the Fermi level for metallic systems. For spin-polarized systems, the black and red lines indicate the spin up and down.

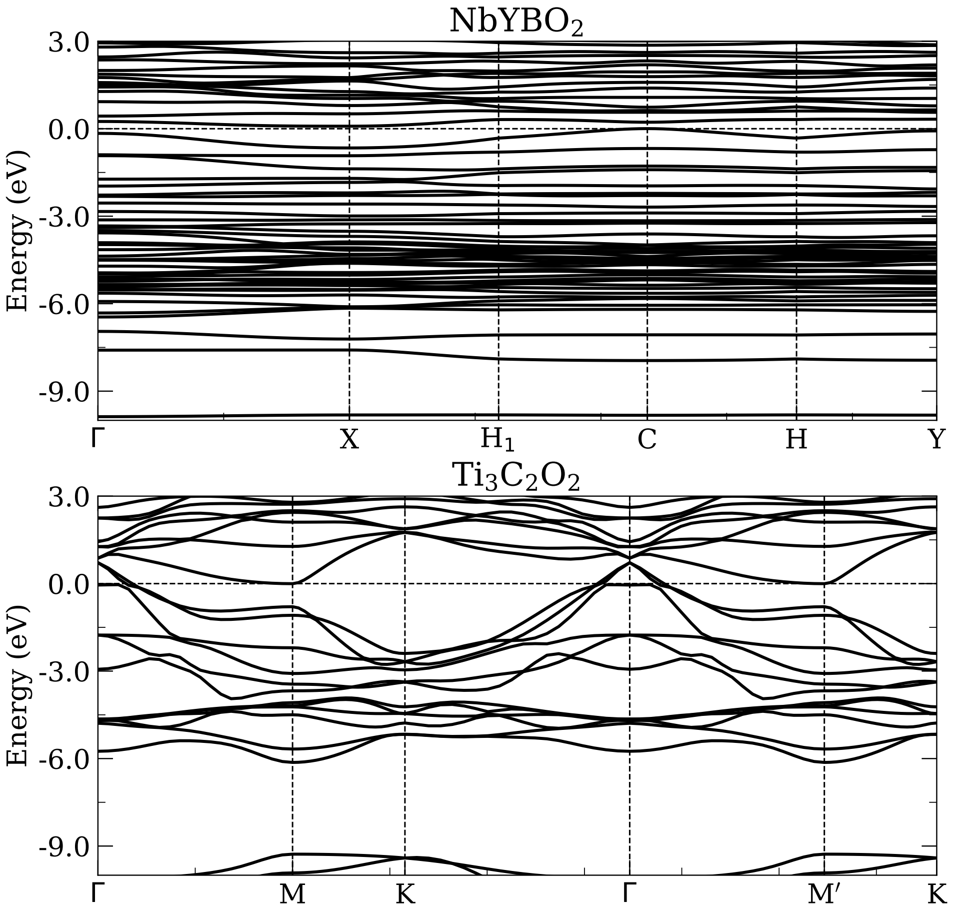

Figure S-24: Band structure for $NbYBO_2$ and $Ti_3C_2O_2$. We considered the lowest energy structure for all compositions. The horizontal black dashed lines indicate the valence band maximum (VBM) for semiconductors or the Fermi level for metallic systems.

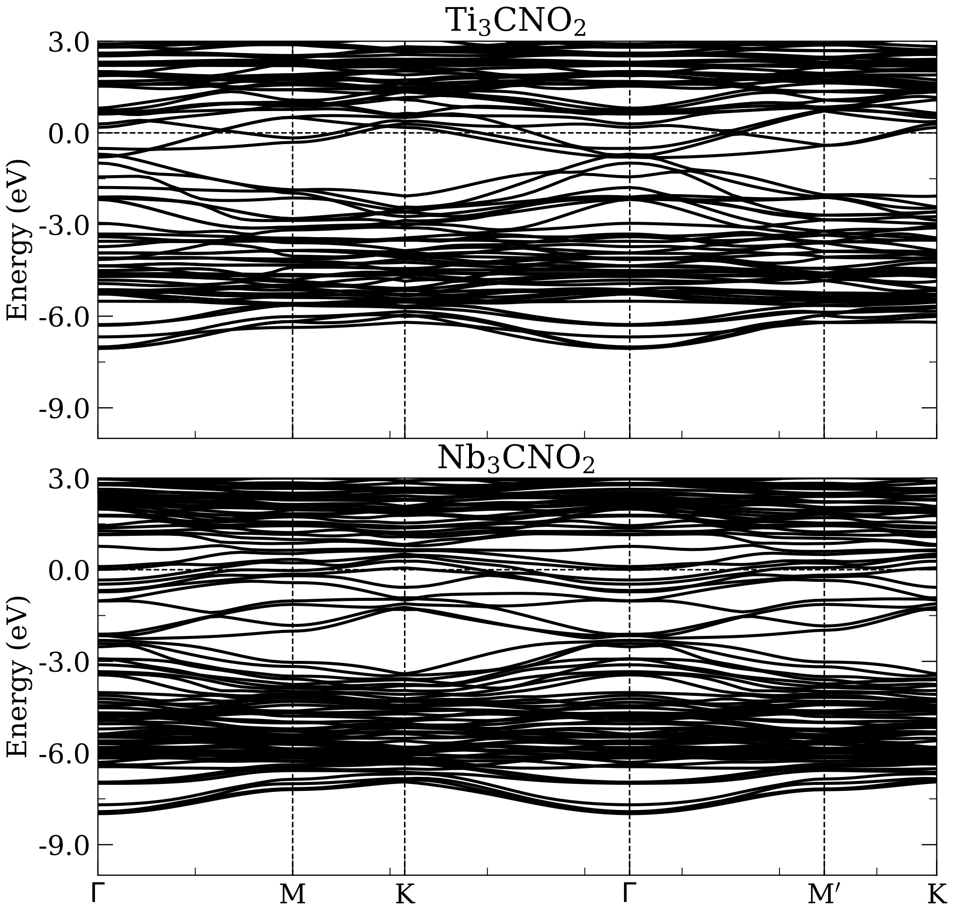

Figure S-25: Band structure for Ti_3CNO_2 and Nb_3CNO_2. We considered the lowest energy structure for all compositions. The horizontal black dashed lines indicate the valence band maximum (VBM) for semiconductors or the Fermi level for metallic systems.

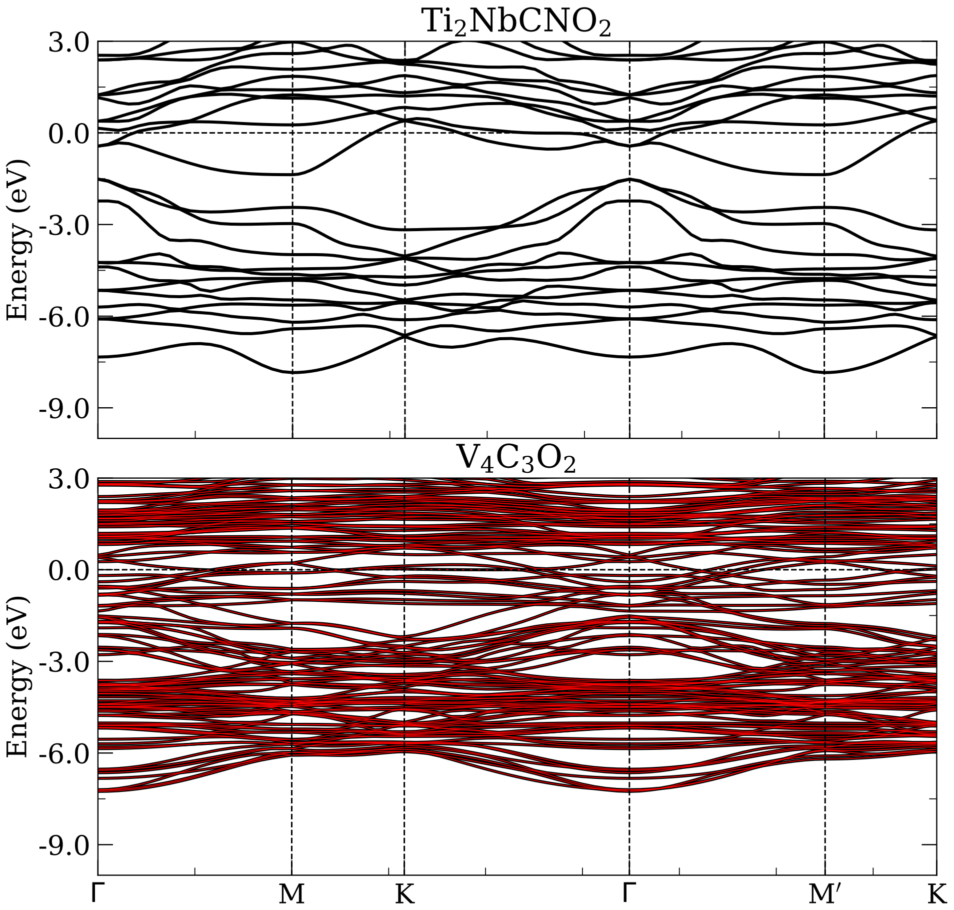

Figure S-26: Band structure for Ti_2NbCNO_2 and $V_4C_3O_2$. We considered the lowest energy structure for all compositions. The horizontal black dashed lines indicate the valence band maximum (VBM) for semiconductors or the Fermi level for metallic systems.

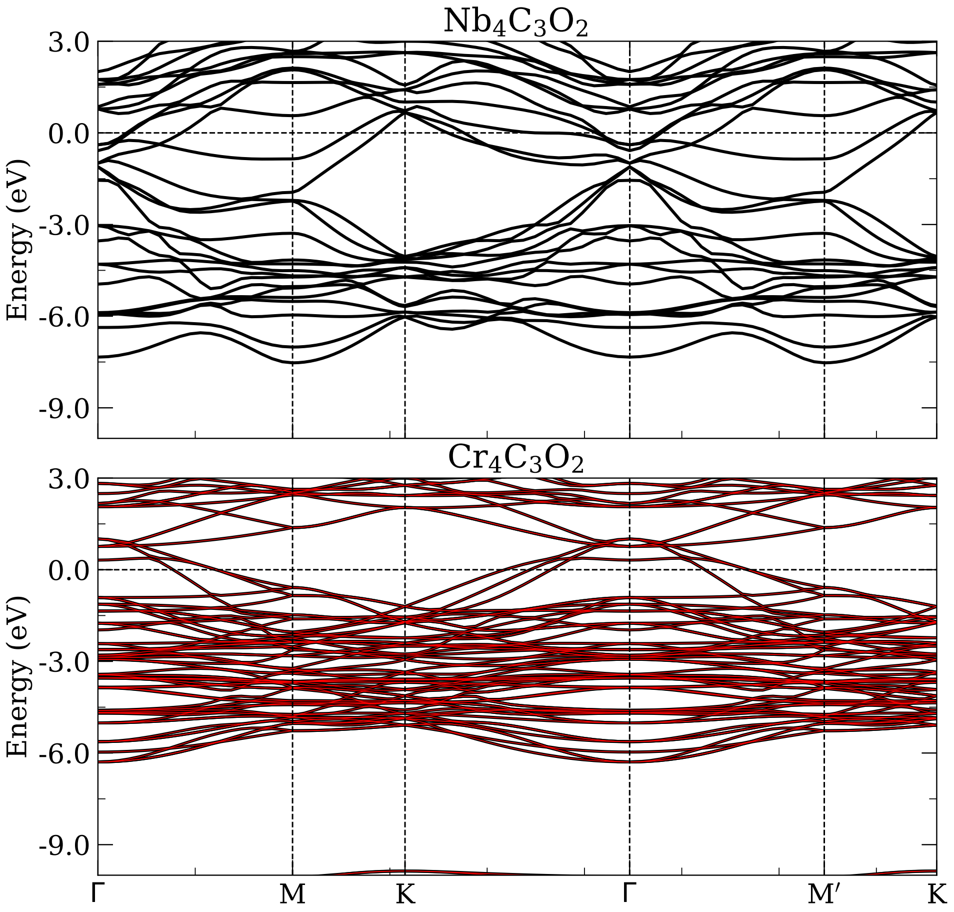

Figure S-27: Band structure for $Nb_4C_3O_2$ and $Cr_4C_3O_2$. We considered the lowest energy structure for all compositions. The horizontal black dashed lines indicate the valence band maximum (VBM) for semiconductors or the Fermi level for metallic systems.

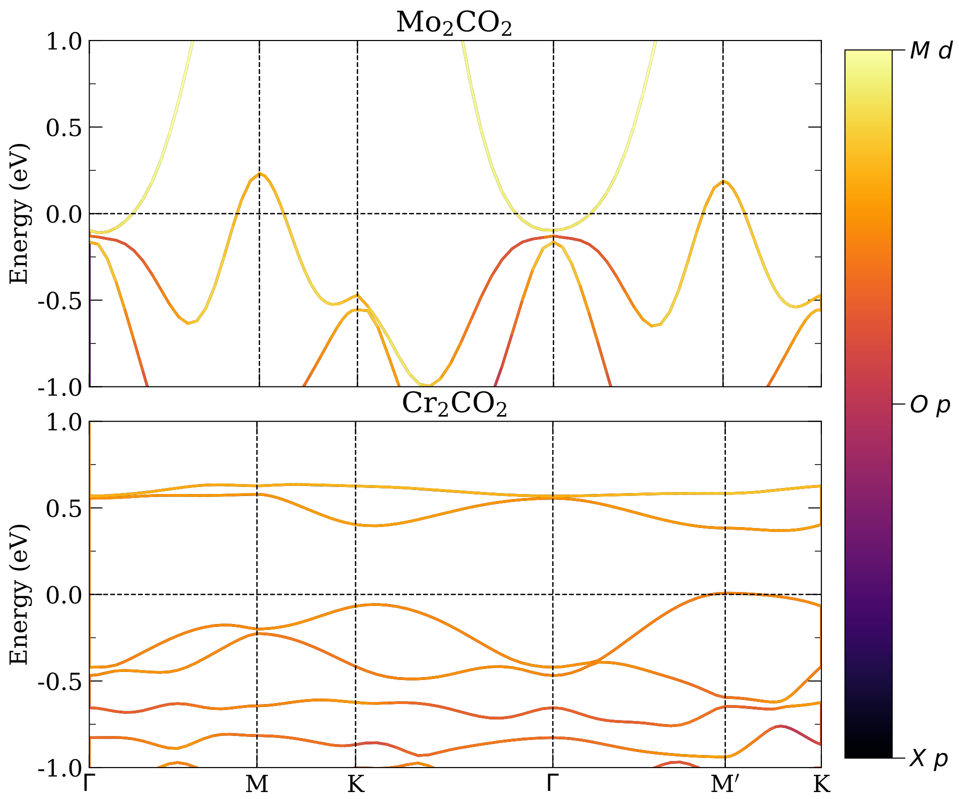

Figure S-28: Band structure for Mo$_2$CO$_2$ and Cr$_2$CO$_2$. We considered the lowest energy structure for all compositions. The horizontal black dashed lines indicate the valence band maximum (VBM) for semiconductors or the Fermi level for metallic systems. For the color coding, we considered the general formula $(M'M'')_{n+1}(X'X'')_n$O$_2$ and for MXenes with two different metals or X elements, we summed the contributions.

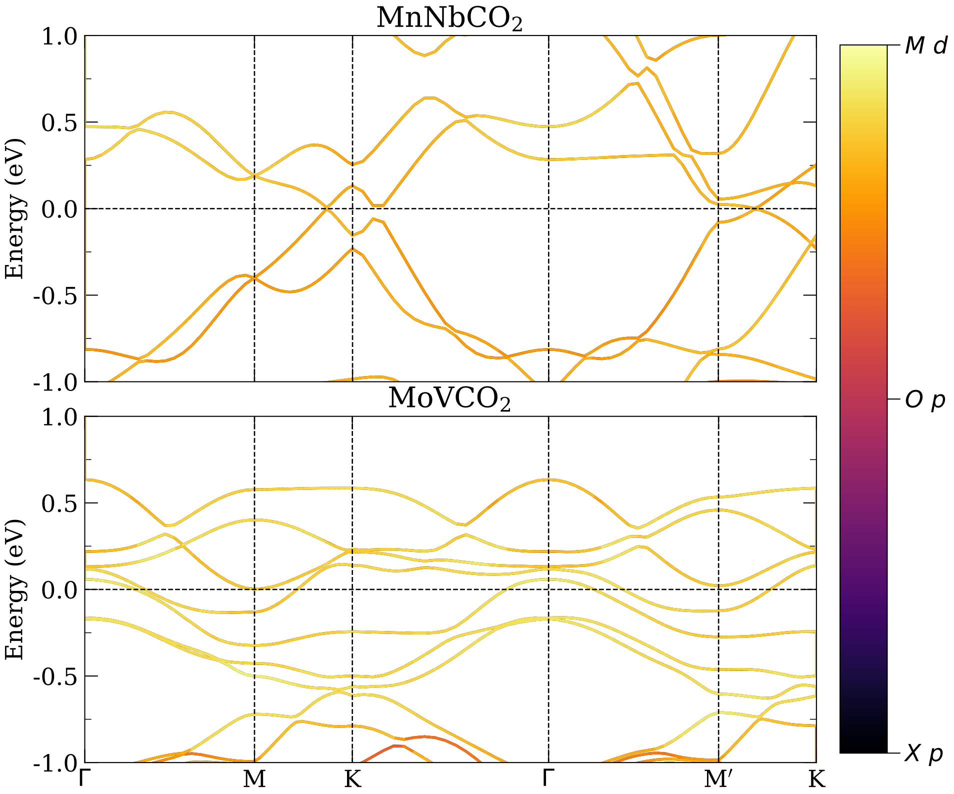

Figure S-29: Band structure for $MnNbCO_2$ and $MoVCO_2$. We considered the lowest energy structure for all compositions. The horizontal black dashed lines indicate the valence band maximum (VBM) for semiconductors or the Fermi level for metallic systems. The horizontal black dashed lines indicate the Fermi level. For the color coding, we considered the general formula $(M'M'')_{n+1}(X'X'')_nO_2$ and for MXenes with two different metals or X elements, we summed the contributions.

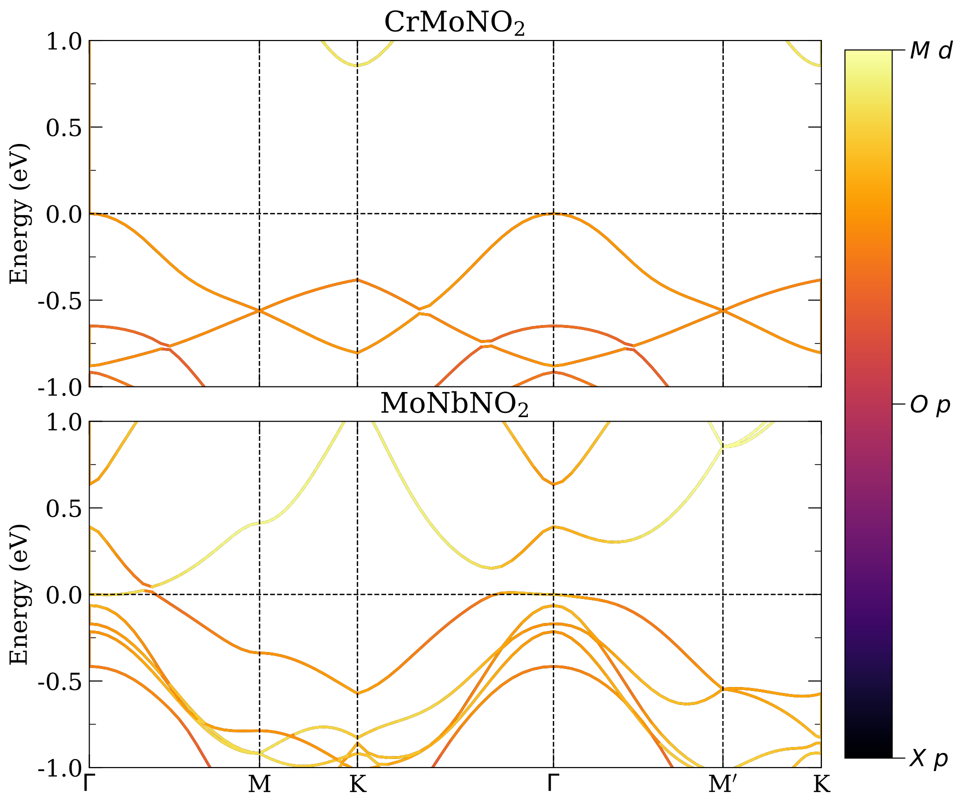

Figure S-30: Band structure for $CrMoNO_2$ and $MoNbNO_2$. We considered the lowest energy structure for all compositions. The horizontal black dashed lines indicate the valence band maximum (VBM) for semiconductors or the Fermi level for metallic systems. The horizontal black dashed lines indicate the Fermi level. For the color coding, we considered the general formula $(M'M'')_{n+1}(X'X'')_nO_2$ and for MXenes with two different metals or X elements, we summed the contributions.

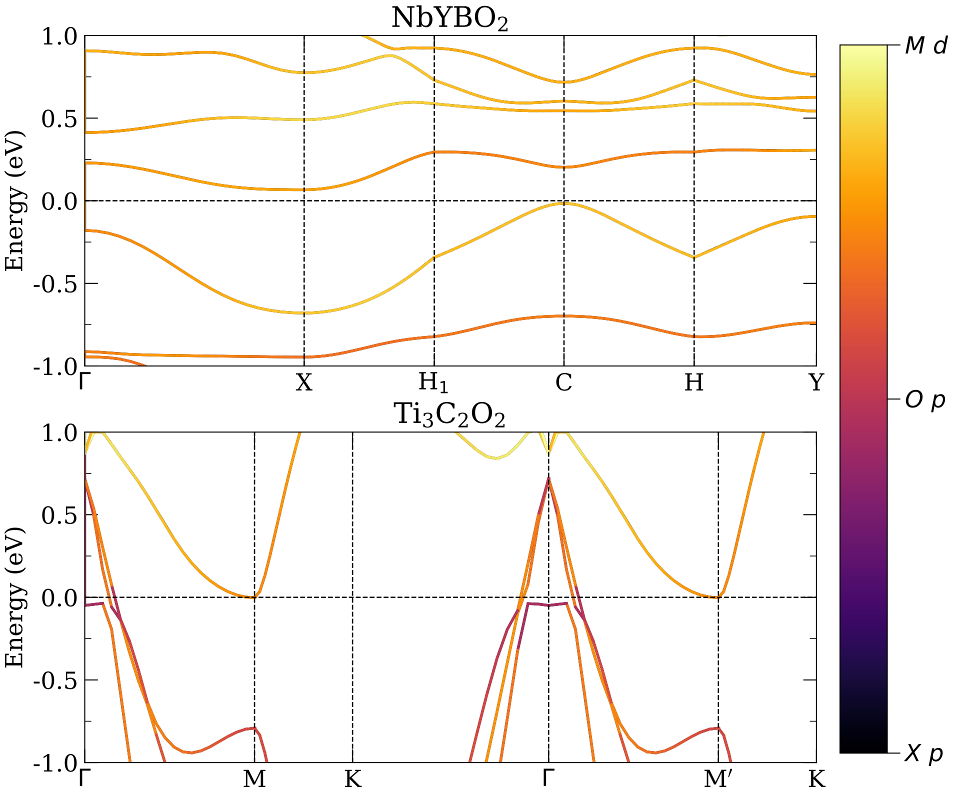

Figure S-31: Band structure for $NbYBO_2$ and $Ti_3C_2O_2$. We considered the lowest energy structure for all compositions. The horizontal black dashed lines indicate the valence band maximum (VBM) for semiconductors or the Fermi level for metallic systems. The horizontal black dashed lines indicate the Fermi level. For the color coding, we considered the general formula $(M'M'')_{n+1}(X'X'')_nO_2$ and for MXenes with two different metals or X elements, we summed the contributions.

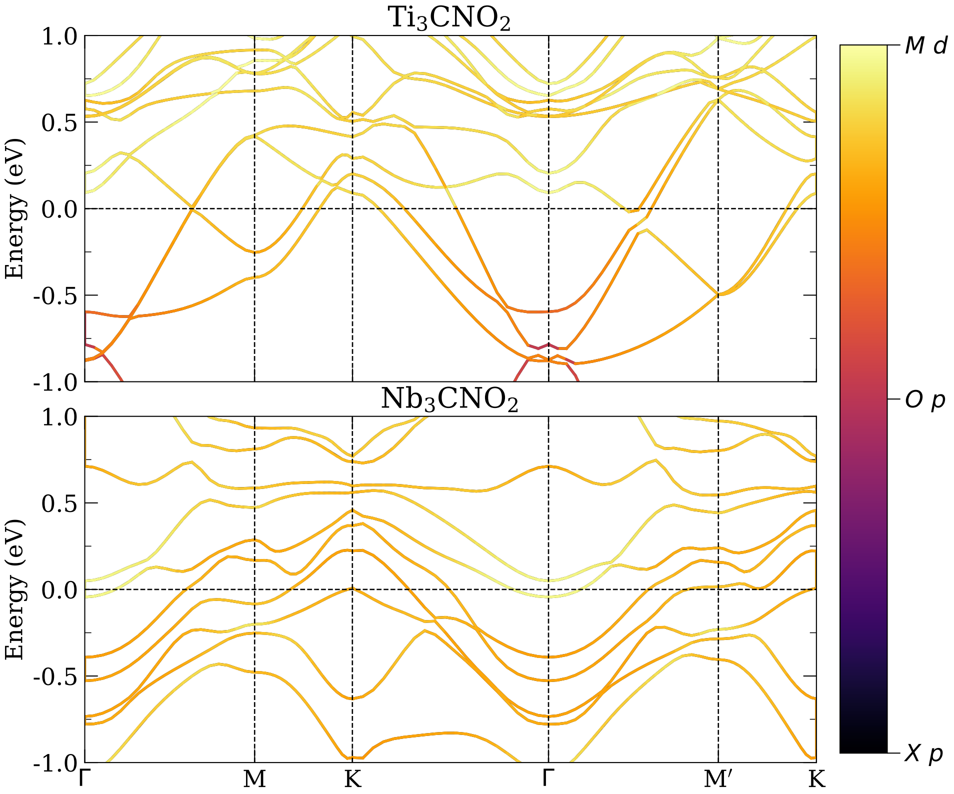

Figure S-32: Band structure for Ti_3CNO_2 and Nb_3CNO_2. We considered the lowest energy structure for all compositions. The horizontal black dashed lines indicate the valence band maximum (VBM) for semiconductors or the Fermi level for metallic systems. The horizontal black dashed lines indicate the Fermi level. For the color coding, we considered the general formula $(M'M'')_{n+1}(X'X'')_nO_2$ and for MXenes with two different metals or X elements, we summed the contributions.

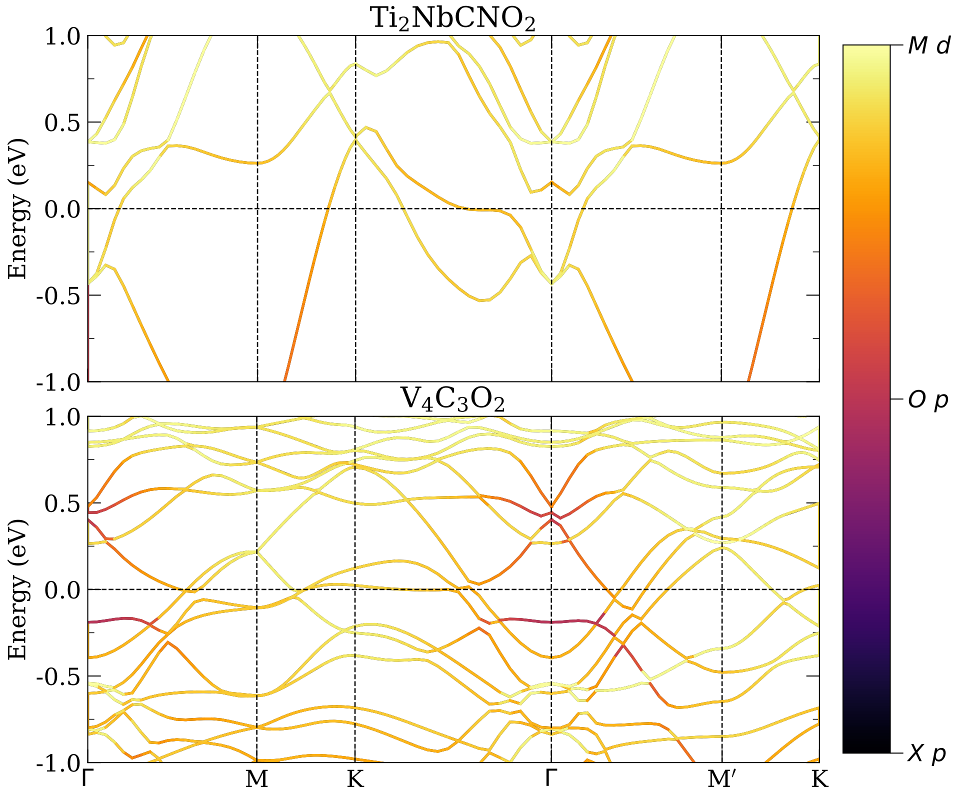

Figure S-33: Band structure for Ti_2NbCNO_2 and $V_4C_3O_2$. We considered the lowest energy structure for all compositions. The horizontal black dashed lines indicate the valence band maximum (VBM) for semiconductors or the Fermi level for metallic systems. The horizontal black dashed lines indicate the Fermi level. For the color coding, we considered the general formula $(M'M'')_{n+1}(X'X'')_nO_2$ and for MXenes with two different metals or X elements, we summed the contributions.

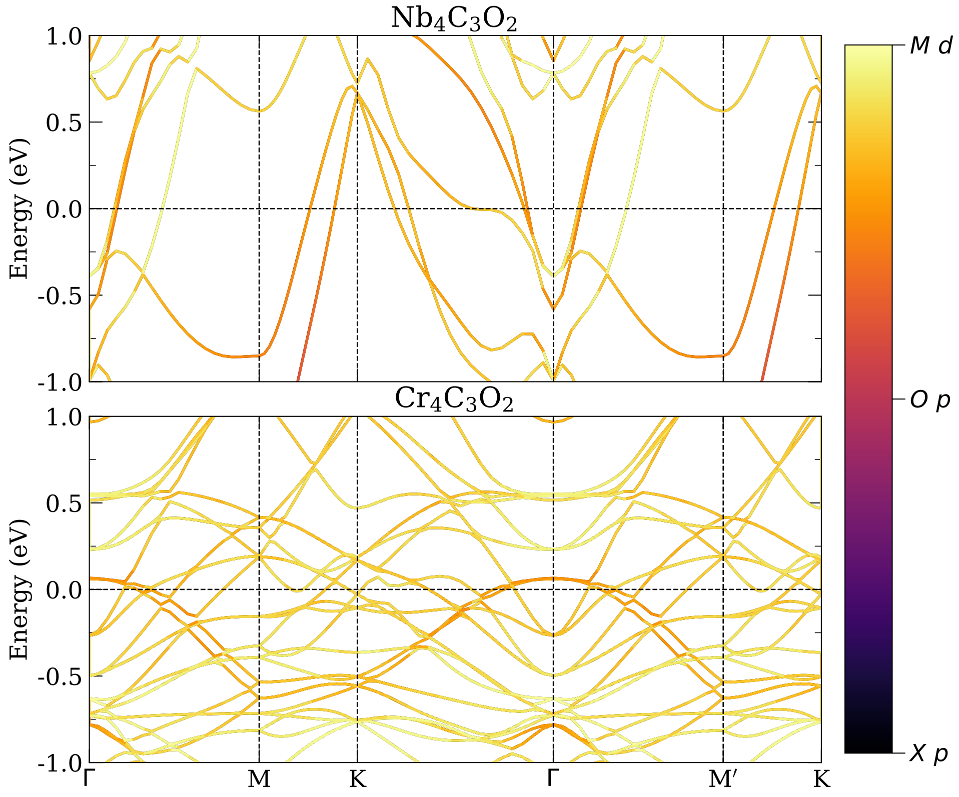

Figure S-34: Band structure for $Nb_4C_3O_2$ and $Cr_4C_3O_2$. We considered the lowest energy structure for all compositions. The horizontal black dashed lines indicate the valence band maximum (VBM) for semiconductors or the Fermi level for metallic systems. The horizontal black dashed lines indicate the Fermi level. For the color coding, we considered the general formula $(M'M'')_{n+1}(X'X'')_nO_2$ and for MXenes with two different metals or X elements, we summed the contributions.

Figures S-20–S-34 show the band structure results and orbital contributions to each band. We used 20 points to sample each path. We did not explored the z-axis because there is no electronic dispersion in this direction due to vacuum.[20,21] Most MXenes have parabolic dispersions, except for $Ti_3C_2O_2$, which has a linear dispersion near the Fermi level.

S-5.4 Work Function

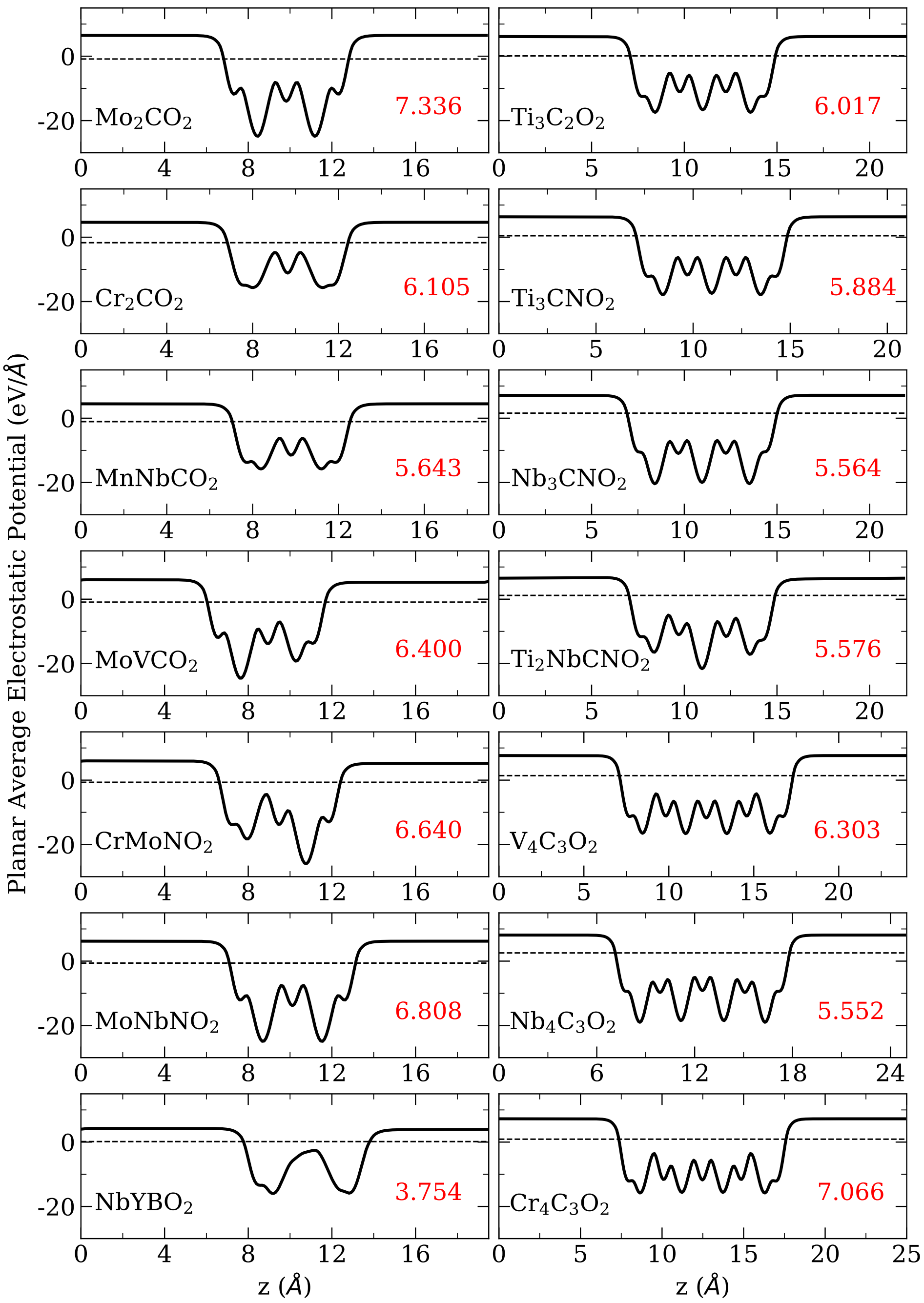

Figure S-35: Planar average electrostatic potential for the lowest energy structure for all compositions. The work function, in eV, is indicated by the red numbers. For asymmetric slabs, we show only the smaller value of the work function. The black dashed lines indicate the Fermi level.

Figure S-35 shows the planar average electrostatic potential along the direction perpendicular to the surface for all systems. This analysis is important to obtain quantitative insight into the morphology and symmetry. We observed that for single-metals and for i^M-MXenes and single

metal MXenes, the planar average potential is symmetric, that is, Mo_2CO_2, Cr_2CO_2, $MnNbCO_2$ and $MoNbNO_2$. For o^M-MXene, the curve is non-symmetric; e.g., for $MoVCO_2$ and $CrMoNO_2$, the potential has a greater negative value in the region of Mo atoms due to the larger number of electrons of Mo, that is, 42, 23 and 24 for Mo, V and Cr, respectively. Furthermore, the C or N distribution does not change the electrostatic potential due to a similar number of electrons. In the case of Cr_2CO_2 and $NbYBO_2$, the curve is notably different from the others due to the distorted structure.

S-5.5 Electron Density Analysis

The key concept of density derived electrostatic and chemical (DDEC6) charges is the use of vectorized charge partitioning:

$$\frac{\rho(\mathbf{r}_A)}{\rho(\mathbf{r})} = \frac{w_a(\mathbf{r}_A)}{W(\mathbf{r})}, \tag{1}$$

$$W(\mathbf{r}) = \sum_{A,L} w_a(\mathbf{r}_A), \tag{2}$$

where $\rho(\mathbf{r}_A)$ is the electronic density assigned to atom A, $\rho(\mathbf{r})$ is the total electronic density, $w_a(\mathbf{r}_A)$ is a spherically symmetric atomic weighting factor, and $W(\mathbf{r})$ is the sum over all atoms. Note that the larger $\rho(\mathbf{r}_A)$, the larger $w_a(\mathbf{r}_A)$, that is, $w_a(\mathbf{r}_A)$ must resemble the reference ion but also must account for the specific material in the study indicated by $\rho(\mathbf{r}_A)$.

In VASP, the DDEC6 analysis requires several steps:

1. A single point calculation is performed with the flags `LCHARG = .TRUE., LAECHG = .TRUE.` to write the charge densities.

2. One must set `ADDGRID = .FALSE.` and define the grid manually. A good recipe is to use the command "grep "dimension x,y,z NGXF" OUTCAR", then triplicate the value, e.g., for our MXenes, we set `NGXF = 288, NGYF = 288, NGZF = 864`.

3. Once the charge densities were written to the `CHGCAR` file, then we are ready to run the DDEC6 analysis. However, due to incompatibilities in the version between the DDEC6

and PAW files, sometimes it may be necessary to remove lines four to seven of the `POTCAR` files.

4. Note that to execute the DDEC6 analysis, every directory must contain a file specifying the periodicity of the system and the net charge. More information can be found in the documentation.[22]

S-5.6 Electron Localization Function

Figures S-36–S-37 show the results for the electron localization function in different planes. This analysis is sensitive to the choice of plane. In the manuscript, we chose the plane with the highest number of bonds, which allows for more accurate analyses. Overall, our results show that Cr_2CO_2, $MnNbCO_2$, $MoVCO_2$, $CrMoNO_2$ and $NbYBO_2$ have a larger ionic character than other MXenes, i.e., no localization between nuclei (ELF$\approx$ 0) and strong localization on nuclei (ELF$\approx$ 1). For this analysis, the contrast is important, e.g., the system $Cr_4C_3O_2$ does not have a large ionic character because there is no localization between and on the nuclei, i.e., the ELF is smaller than 0.5 in all regions of this system.

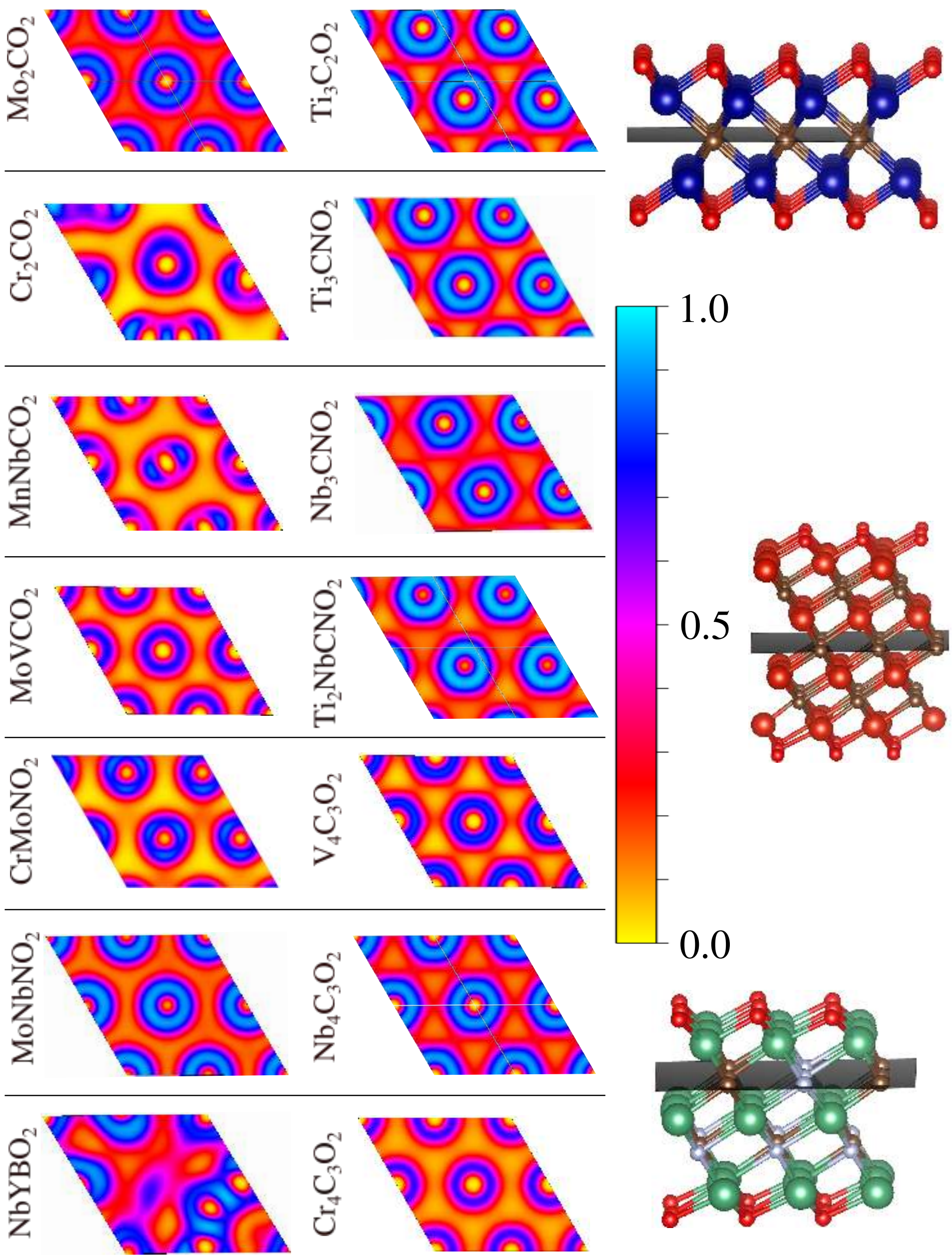

Figure S-36: Electron localization function (dimensionless) for the optimized lowest energy structures of all selected MXene compositions. The selected *X* plane is indicated.

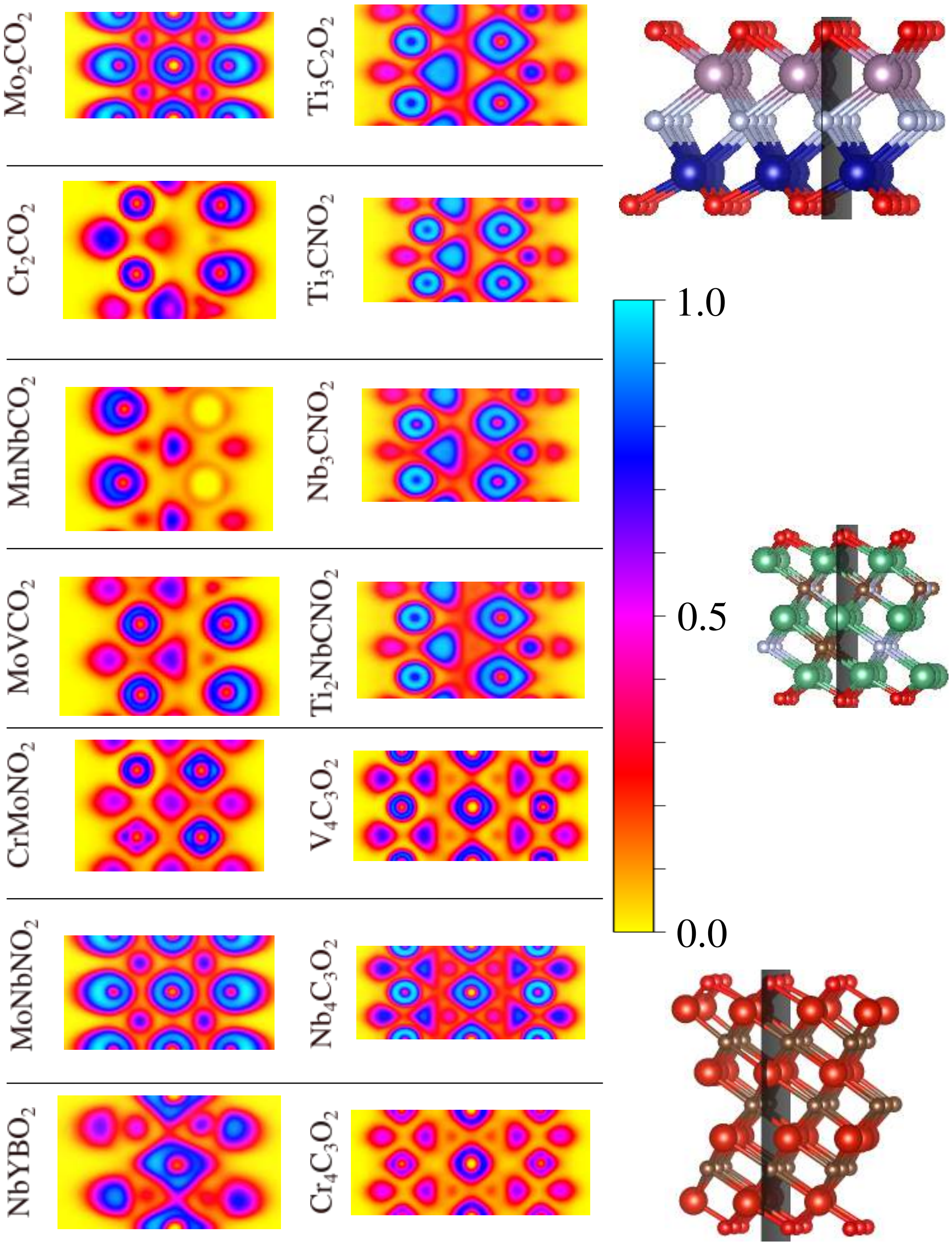

Figure S-37: Electron localization function (dimensionless) for lowest energy structures of all compositions. The (100) plane was considered.

References

1 VahidMohammadi, A.; Rosen, J.; Gogotsi, Y. The World of Two-dimensional Carbides and Nitrides (MXenes). *Science* **2021**, *372*, DOI: `10.1126/science.abf1581`.

2 Cheng, Y.; Wang, L.; Song, Y.; Zhang, Y. Deep Insights Into the Exfoliation Properties of

MAX to MXenes and the Hydrogen Evolution Performances of 2D MXenes. *J. Mater. Chem. A* **2019**, *7*, 15862–15870, DOI: 10.1039/C9TA03859K.

3 Wan, P.; Tang, Q. The Effect of Constant Potential on the Hydrogen Evolution Reaction Activity of M_2CO_2 and M_2NO_2 MXenes. *ChemPhysChem* **2023**, *24*, e202200871, DOI: 10.1002/cphc.202200871.

4 Zou, X.; Liu, H.; Xu, H.; Wu, X.; Han, X.; Kang, J.; Reddy, K. M. A Simple Approach to Synthesis Cr_2CT_x MXene for Efficient Hydrogen Evolution Reaction. *Mater. Today Energy* **2021**, *20*, 100668, DOI: 10.1016/j.mtener.2021.100668.

5 Abraham, B. M.; Sinha, P.; Halder, P.; Singh, J. K. Fusing a Machine Learning Strategy with Density Functional Theory to Hasten the Discovery of 2D MXene-based Catalysts for Hydrogen Generation. *J. Mater. Chem. A* **2023**, *11*, 8091–8100, DOI: 10.1039/D3TA00344B.

6 Jiang, Y.; Sun, T.; Xie, X.; Jiang, W.; Li, J.; Tian, B.; Su, C. Oxygen-Functionalized Ultrathin $Ti_3C_2T_x$ MXene for Enhanced Electrocatalytic Hydrogen Evolution. *ChemSusChem* **2019**, *12*, 1368–1373, DOI: 10.1002/cssc.201803032.

7 Cao, D.; Liu, H.; Sun, Q.; Wang, X.; Wang, J.; Liu, C.; Liu, C.; Hao, Q.; Li, Y. Tailoring the Electronic Structure by Transition-metal Single Atom on $Ti_3C_2O_2$ for Improving Hydrogen Evolution Activity. *Appl. Surf. Sci.* **2021**, *563*, 150210, DOI: 10.1016/j.apsusc.2021.150210.

8 Huang, B.; Zhou, N.; Chen, X.; Ong, W.-J.; Li, N. Insights into the Electrocatalytic Hydrogen Evolution Reaction Mechanism on Two-Dimensional Transition-Metal Carbonitrides (MXene). *Chem. Eur. J.* **2018**, *24*, 18479–18486, DOI: 10.1002/chem.201804686.

9 Zeng, Z.; Chen, X.; Weng, K.; Wu, Y.; Zhang, P.; Jiang, J.; Li, N. Computational Screening Study of Double Transition Metal Carbonitrides $M'_2M''CNO_2$-MXene as Catalysts for Hydrogen Evolution Reaction. *npj Comput. Mater.* **2021**, *7*, 1–7, DOI: 10.1038/s41524-021-00550-4.

10 Tran, M. H.; Schäfer, T.; Shahraei, A.; Dürrschnabel, M.; Molina-Luna, L.; Kramm, U. I.; Birkel, C. S. Adding a New Member to the MXene Family: Synthesis, Structure, and Electrocatalytic Activity for the Hydrogen Evolution Reaction of $V_4C_3T_x$. *ACS Appl. Energy Mater.* **2018**, *1*, 3908–3914, DOI: 10.1021/acsaem.8b00652.

11 Tan, Y.; Zhu, Z.; Zhang, X.; Zhang, J.; Zhou, Y.; Li, H.; Qin, H.; Bo, Y.; Pan, Z. $Nb_4C_3T_x$ (MXene) as a New Stable Catalyst for the Hydrogen Evolution Reaction. *Int. J. Hydrogen Energy* **2021**, *46*, 1955–1966, DOI: 10.1016/j.ijhydene.2020.10.046.

12 Šljivančanin, Ž. Optimizing Hydrogen Evolution Reaction: Computational Screening of Single Metal Atom Impurities in 2D MXene $Nb_4C_3O_2$. *Front. Phys.* **2024**, *19*, 1–8, DOI: 10.1007/s11467-024-1392-9.

13 Pandey, M.; Thygesen, K. S. Two-Dimensional MXenes as Catalysts for Electrochemical Hydrogen Evolution: A Computational Screening Study. *J. Phys. Chem. C* **2017**, *121*, 13593–13598, DOI: 10.1021/acs.jpcc.7b05270.

14 Blöchl, P. E. Projector Augmented-Wave Method. *Phys. Rev. B* **1994**, *50*, 17953–17979, DOI: 10.1103/physrevb.50.17953.

15 Tan, Z.; Fang, Z.; Li, B.; Yang, Y. First-Principles Study of the Ferromagnetic Properties of Cr_2CO_2 and Cr_2NO_2 MXenes. *ACS Omega* **2020**, *5*, 25848–25853, DOI: 10.1021/acsomega.0c03176.

16 Bera, S.; Kumar, H. Phase Stability of MXenes: Understanding the Role of Coordination Symmetries, Transition Metals, and Surface Terminations. *J. Phys. Chem. C* **2023**, *127*, 20734–20741, DOI: 10.1021/acs.jpcc.3c04183.

17 Huang, B.; Li, N.; Ong, W.-J.; Zhou, N. Single Atom-supported MXene: How Single-atomic-site Catalysts Tune the High Activity and Selectivity of Electrochemical Nitrogen Fixation. *J. Mater. Chem. A* **2019**, *7*, 27620–27631, DOI: 10.1039/C9TA09776G.

18 Li, L. Lattice Dynamics and Electronic Structures of $Ti_3C_2O_2$ and $Mo_2TiC_2O_2$ (MXenes): The Effect of Mo Substitution. *Comput. Mater. Sci.* **2016**, *124*, 8–14, DOI: 10.1016/j.commatsci.2016.07.008.

19 Wang, X.; Lin, S.; Tong, H.; Huang, Y.; Tong, P.; Zhao, B.; Dai, J.; Liang, C.; Wang, H.; Zhu, X. et al. Two-dimensional V_4C_3 MXene as High Performance Electrode Materials for Supercapacitors. *Electrochim. Acta* **2019**, *307*, 414–421, DOI: 10.1016/j.electacta.2019.03.205.

20 Hinuma, Y.; Pizzi, G.; Kumagai, Y.; Oba, F.; Tanaka, I. Band Structure Diagram Paths Based on Crystallography. *Comput. Mater. Sci.* **2017**, *128*, 140–184, DOI: 10.1016/j.commatsci.2016.10.015.

21 Setyawan, W.; Curtarolo, S. High-throughput Electronic Band Structure Calculations: Challenges and Tools. *Comput. Mater. Sci.* **2010**, *49*, 299–312, DOI: 10.1016/j.commatsci.2010.05.010.

22 Manz, T. A.; Limas, N. G. Introducing DDEC6 Atomic Population Analysis: Part 1. Charge Partitioning Theory and Methodology. *RSC Adv.* **2016**, *6*, 47771–47801, DOI: 10.1039/C6RA04656H.
